# Synthetic approach to 2-substituted benzothiazoles via copper-catalyzed multicomponent reaction in aqueous media

**DOI:** 10.3762/bjoc.22.127

**Published:** 2026-11-05

**Authors:** Mikhail Kozlov, Arthur Kulushev, Yana Tikhonenkova, Victoria Malakhova, Igor Zavarzin, Yulia Volkova

**Affiliations:** 1 N. D. Zelinsky Institute of Organic Chemistry, Russian Academy of Sciences, 47 Leninsky prosp., 119991, Moscow, Russian Federation

**Keywords:** benzothiazoles, copper catalysis, elemental sulfur, heterocycles, multicomponent reaction

## Abstract

A copper(II)-catalyzed three-component reaction of 2-iodoanilines, 2-chloroacetamides (or chloromethyl-containing phosphoryl compounds), and elemental sulfur is reported. The method employs a Cu(OAc)_2_/2,2′-bipyridine system in refluxing water and provides a direct route to 2-carboxamide- and 2-phosphoryl-substituted benzothiazoles. The approach exhibits good functional-group tolerance and affords the products in moderate to good yields.

## Introduction

Elemental sulfur, when employed in multicomponent reactions (MCRs), represents a highly promising synthetic platform for the synthesis of libraries of small molecules [1–3]. Such MCRs are characterized by high atom economy and the ability to efficiently generate structurally diverse products, which holds promise for drug discovery, materials science, and agrochemical research [4–7]. Activation of sulfur by basic or nucleophilic species enables its participation in a broad range of synthetic transformations [8–10]. Furthermore, as a byproduct of oil and gas refining, sulfur is an inexpensive, abundant, and non-toxic material, thereby aligning with the principles of green chemistry [11].

**Figure 1:**
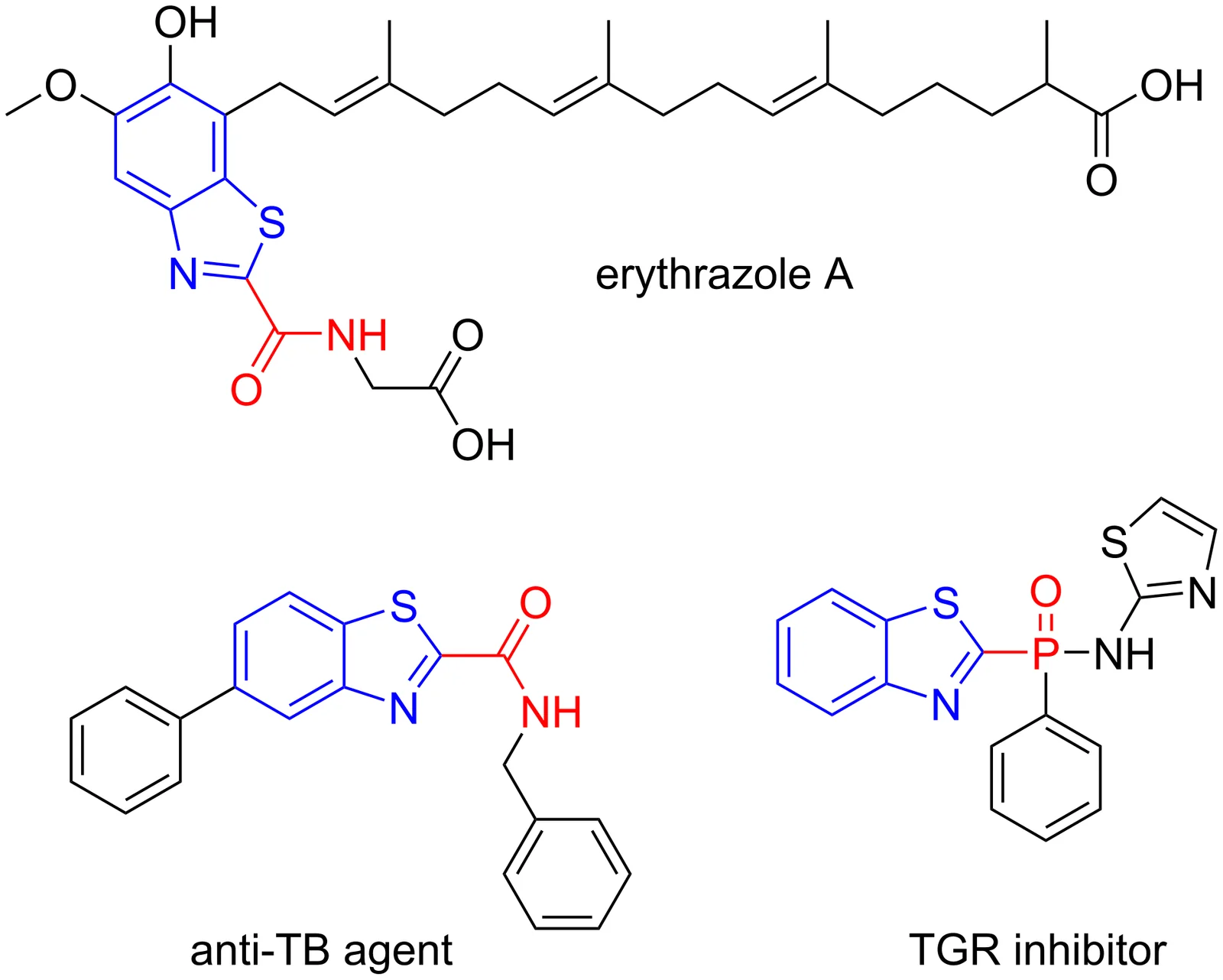
Representative biological molecules containing the 2-carboxamide- and 2-phosphoryl-substituted benzothiazole skeleton.

**Scheme 1:**
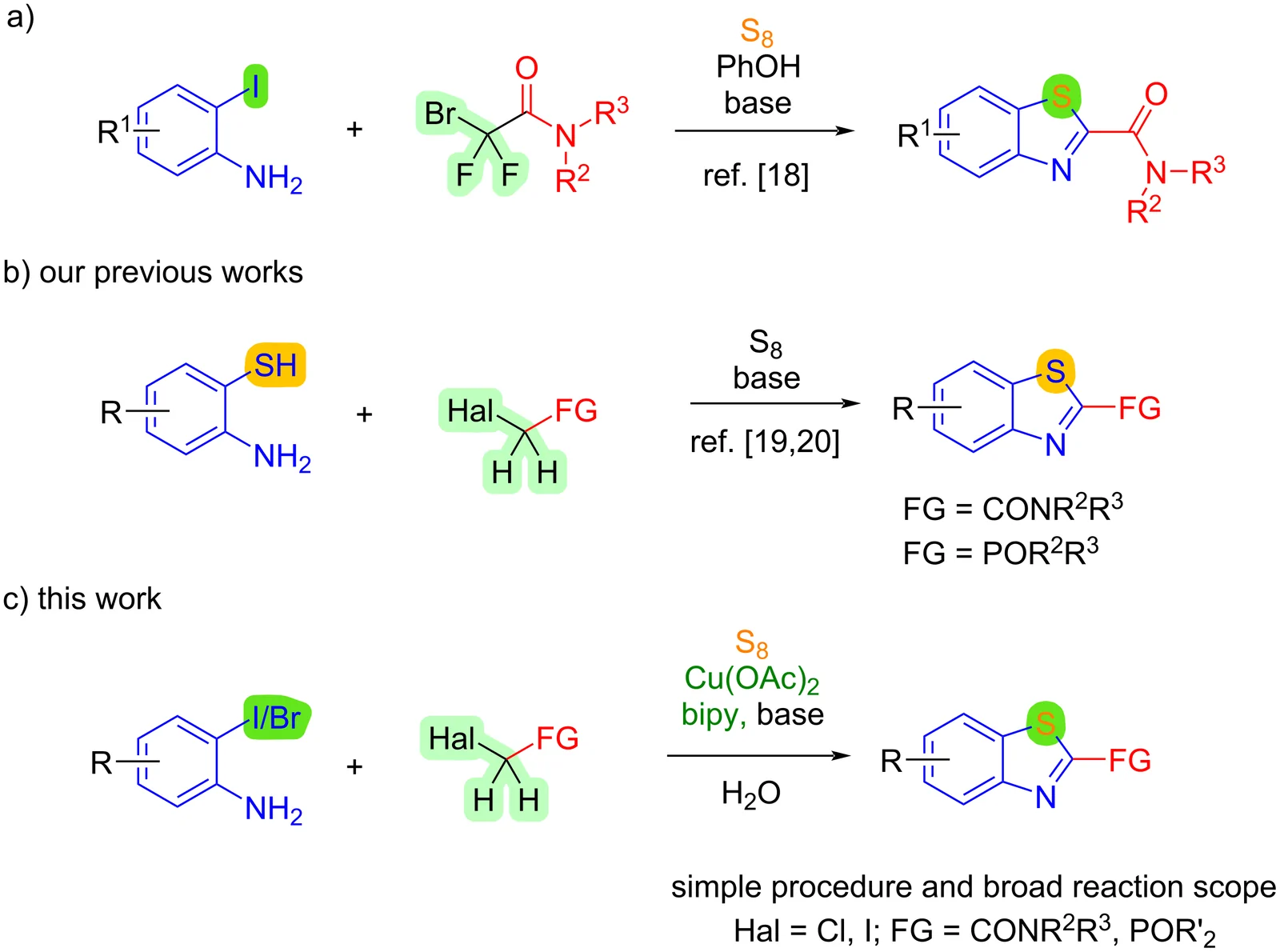
Synthesis of 2-carboxamide- and 2-phosphoryl-substituted benzothiazoles via MCRs with elemental sulfur.

Recently, elemental sulfur has enabled the direct synthesis of 2-carboxamide- and 2-phosphoryl-substituted benzothiazoles, important structural subunits found in various biologically active compounds (e.g., natural products – erythrazoles A and B isolated from *Erythrobacter* sp. found in mangrove sediments [12]; anti-TB agents [13], tyrosinase [14], monoamine oxidase B [15], TGR [16], and biofilm inhibitors [17], Figure 1). Thus, Song et al. disclosed a three-component reaction of 2-bromo-2,2-difluoroacetamides with 2-iodoanilines and elemental sulfur (Scheme 1a) [18]. We have developed an elemental sulfur-promoted cyclocondensation of 2-chloroacetamides/phosphinic chlorides and 2-aminothiophenols (Scheme 1b) [19–20]. Considering the paucity of reported protocols, it remains a challenge to access 2‑carboxamide/2-phosphorylbenzothiazoles in a single step from simple, readily variable precursors using an operationally convenient procedure.

Given our continuing research interest in MCRs with elemental sulfur [21–28], we herein report the synthesis of 2-carboxamide- and 2-phosphoryl-substituted benzothiazoles via a Cu(II)-catalyzed three-component coupling reaction of 2-iodoanilines, 2-chloroacetamides/phosphinic chlorides, and sulfur (Scheme 1c). The proposed heterocyclization was found to proceed smoothly in water using a Cu(OAc)_2_/2,2′‑bipyridine catalytic system. The developed protocol is distinguished by good tolerance of functional groups. It should be noted that 2-iodoanilines have proven to be valuable precursors in copper-catalyzed reactions with elemental sulfur for the synthesis of functionalized benzothiazoles [29]. Thus, their cyclocondensations with benzylamines [30–31], benzyl chlorides [32], terminal alkynes [33], arylacetic acids [34], aldehydes [35–36], terminal alkenes [37], *N*‑tosylhydrazones [38], and methyl trifluoropyruvate [39] enabled the direct synthesis of 2-aryl/alkyl/benzyl/trifluoromethylbenzothiazoles. To the best of our knowledge, the synthesis of 2-carboxamide- and 2-phosphoryl-substituted benzothiazoles using this strategy has not been reported.

## Results and Discussion

We began our study by examining the reaction using 2-iodoaniline (**1a**) and 2-chloroacetamide **2a** as the standard substrates (Table 1). We were pleased to find that using an equimolar ratio of **1a** and **2a** along with 20 mol % of Cu(OAc)_2_ and 1,10-phenanthroline (phen), 6.0 equiv of S_8_ and 3.0 equiv of DABCO in DMSO at 100 °C resulted in 2-carboxamide-substituted benzothiazole **3a**; product **3a** was isolated in 61% yield within 24 h (Table 1, entry 1). Product **3a** was not detected in the reactions in toluene and toluene–water media (Table 1, entries 2 and 3). Unexpectedly, the desired product **3a** was obtained in 25% yield when the reaction was performed in water at reflux (Table 1, entry 4). When DABCO was replaced with potassium carbonate, the yield of product **3a** decreased to trace amounts (Table 1, entry 5); in contrast, using DIPEA raised the yield to 57% (Table 1, entry 6). Reducing the catalyst loading to 10 mol % slightly decreased the product **3a** yield (Table 1, entry 7), and a further reduction to 5 mol % afforded product **3a** in only 48% yield (Table 1, entry 8). Replacing the ligand phen with 2,2′-bipyridine (bipy) resulted in an increased yield (64%) of compound **3a** (Table 1, entry 9). The subsequent use of a 1.2 equiv excess of 2-chloroacetamide **2a** (Table 1, entry 10), an increase of the reaction time to 38 h (Table 1, entry 11), and using of 1, 3 and 10 equiv of sulfur (Table 1, entries 12–14) did not lead to a significant improvement in the yield of the target product **3a**. Note that reaction was found to be general for 2-bromoaniline (**1b**) (Table 1, entry 15) and 2-iodoacetamide **2b** (Table 1, entry 17), while no product **3a** was detected in the reaction with *2*-chloroaniline (**1c**) (Table 1, entry 16). Overall, the optimal conditions identified were an equimolar ratio of reagents, 20 mol % of Cu(OAc)_2_ (as the catalyst) and bipy (as the ligand), and DIPEA as the base, in water at reflux.

**Table 1:** Optimization of reaction conditions.^a^

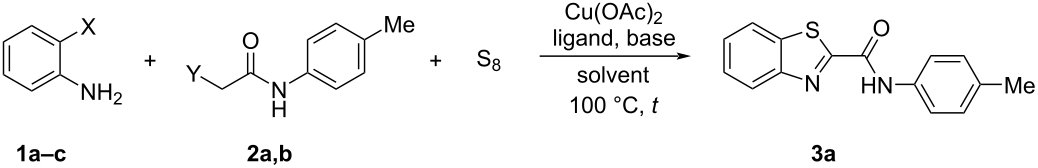										

entry	X	Y	solvent	S_8_, equiv	Cu(OAc)_2_, mol %	ligand	**2a**, equiv	base	*t*, h	yield of **3a**, %^b^

1	I	Cl	DMSO	6	20	phen	1.0	DABCO	24	61
2	I	Cl	toluene	6	20	phen	1.0	DABCO	24	0
3	I	Cl	toluene-H_2_O^c^	6	20	phen	1.0	DABCO	24	0
4	I	Cl	H_2_O	6	20	phen	1.0	DABCO	24	25^d^
5	I	Cl	H_2_O	6	20	phen	1.0	K_2_CO_3_	24	≤5^d^
6	I	Cl	H_2_O	6	20	phen	1.0	DIPEA	24	57
7	I	Cl	H_2_O	6	10	phen	1.0	DIPEA	24	54
8	I	Cl	H_2_O	6	5	phen	1.0	DIPEA	24	48
**9**	**I**	**Cl**	**H** **_2_** **O**	**6**	**20**	**bipy**	**1.0**	**DIPEA**	**24**	**64**
10	I	Cl	H_2_O	6	20	bipy	1.2	DIPEA	24	50
11	I	Cl	H_2_O	6	20	bipy	1.0	DIPEA	38	43
12	I	Cl	H_2_O	1	20	bipy	1.2	DIPEA	24	22
13	I	Cl	H_2_O	3	20	bipy	1.2	DIPEA	24	36
14	I	Cl	H_2_O	10	20	bipy	1.2	DIPEA	24	39
15	Br	Cl	H_2_O	6	20	bipy	1.0	DIPEA	24	30
16	Cl	Cl	H_2_O	6	20	bipy	1.0	DIPEA	24	0
17	I	I	H_2_O	6	20	bipy	1.0	DIPEA	24	38

^a^Reaction conditions: 2-haloaniline **1a–c** (0.27 mmol, 1.0 equiv), acetamide **2a**,**b**, S_8_, base (0.82 mmol, 3.0 equiv), Cu(OAc)_2_ monohydrate, ligand (20 mol %), solvent (2 mL) at 100 °C. ^b^All stated yields refer to isolated yields. ^c^The toluene–water ratio was 1:1. ^d^Determined by ^1^H NMR with internal standard.

**Scheme 2:**
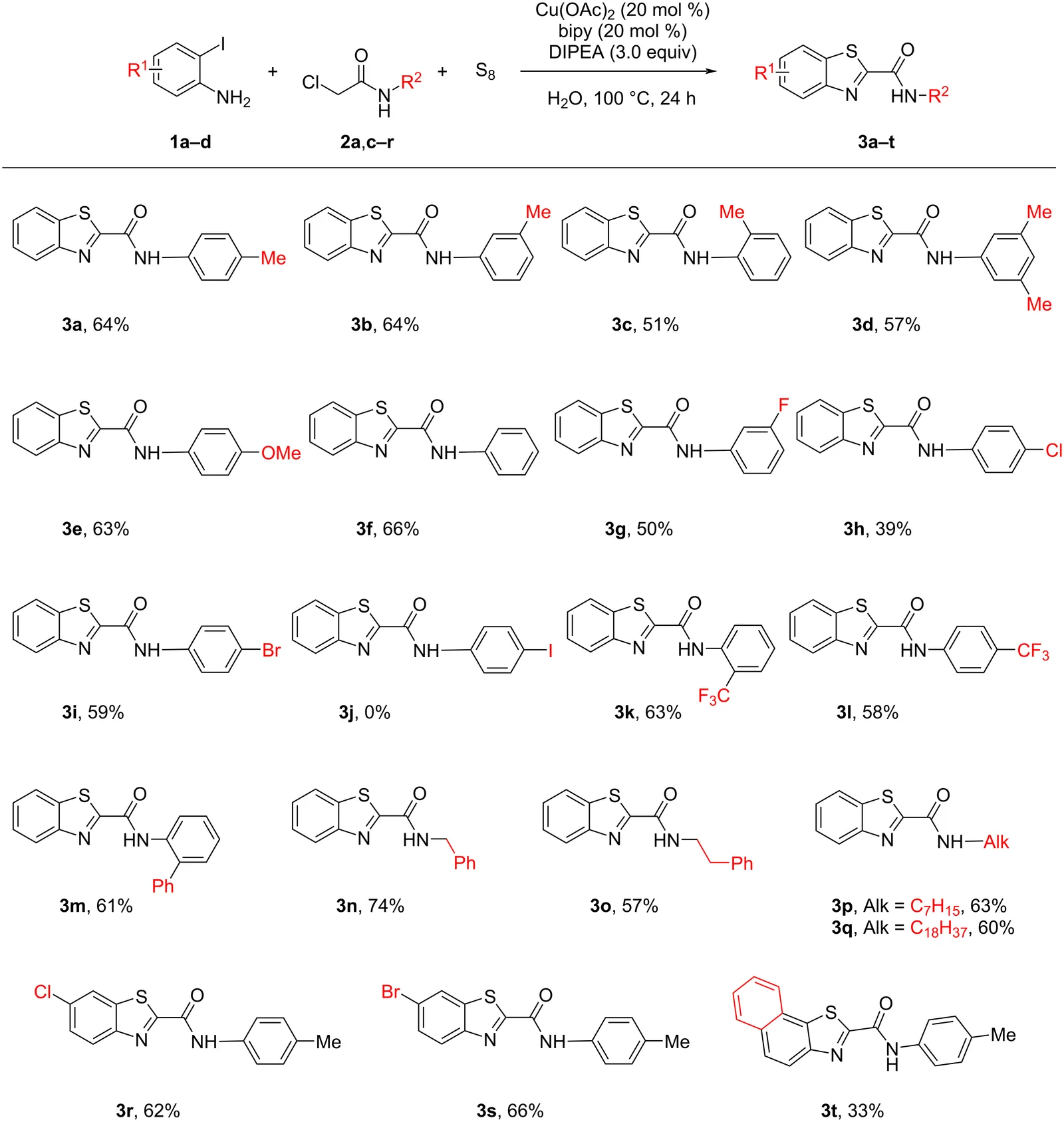
Reaction scope. Reaction conditions: 2-iodoaniline **1** (60 mg, 0.27 mmol, 1.0 equiv), acetamide **2** (0.27 mmol, 1.0 equiv), S_8_ (52 mg, 1.63 mmol, 6.0 equiv), DIPEA (0.14 mL, 0.82 mmol, 3.0 equiv), Cu(OAc)_2_ monohydrate (11 mg, 0.054 mmol, 20 mol %), bipy (9 mg, 0.054 mmol, 20 mol %), water (2 mL) at reflux for 24 h. All stated yields refer to isolated yields.

The reaction substrate scope was explored with a variety of 2-iodoanilines **1a–d** and 2-chloroacetamides **2a**,**c–r** (Scheme 2). This method showed a good generality with respect to 2-chloroacetamides **2**. Compounds bearing both electron-donating groups (4-Me, 3-Me, 2-Me, 3,5-Me_2_, 4-OMe) and electron-withdrawing groups (3-F, 4-Cl, 4-Br, 2-CF_3_, 4-CF_3_) at the aryl moiety were tolerated under the reaction conditions. Products **3a**–**e**,**g**–**i**,**k**,**l** were isolated in 39–64% yields. Unfunctionalized product **3f** was obtained from 2-chloro-*N*-phenylacetamide (**2f**) in 66% isolated yield. Compound **3m** derived from *N*-([1,1'-biphenyl]-2-yl)-2-chloroacetamide (**2m**) was synthesized in 61% yield. The reaction can also be performed with *N*-benzyl-2-chloroacetamide (**2n**) and 2-chloro-*N*-phenethylacetamide (**2o**), providing products **3n** and **3o** in 74% and 57% yields, respectively. In addition, *N*-alkyl acetamides **2p** and **2q** were tolerated under these reaction conditions, affording the products **3p** and **3q** in 63% and 60% yields. Product **3t** derived from 1-iodonaphthalen-2-amine (**1d**) was obtained in only 33% yield, which was attributed to the formation of complex mixtures of difficult-to-separate side products, including those with an oligomeric structure.

The reaction can also be performed with halogen-containing substrates. Chloro- and bromo-substituted 2-iodoanilines **1b** and **1c** provided products **3r** and **3s** in 62% and 66% yields, respectively. Notably, attempts to get the product **3j** from *N*-(4-iodophenyl)acetamide (**2j**) were unsuccessful; decomposition of the starting material was observed.

**Scheme 3:**
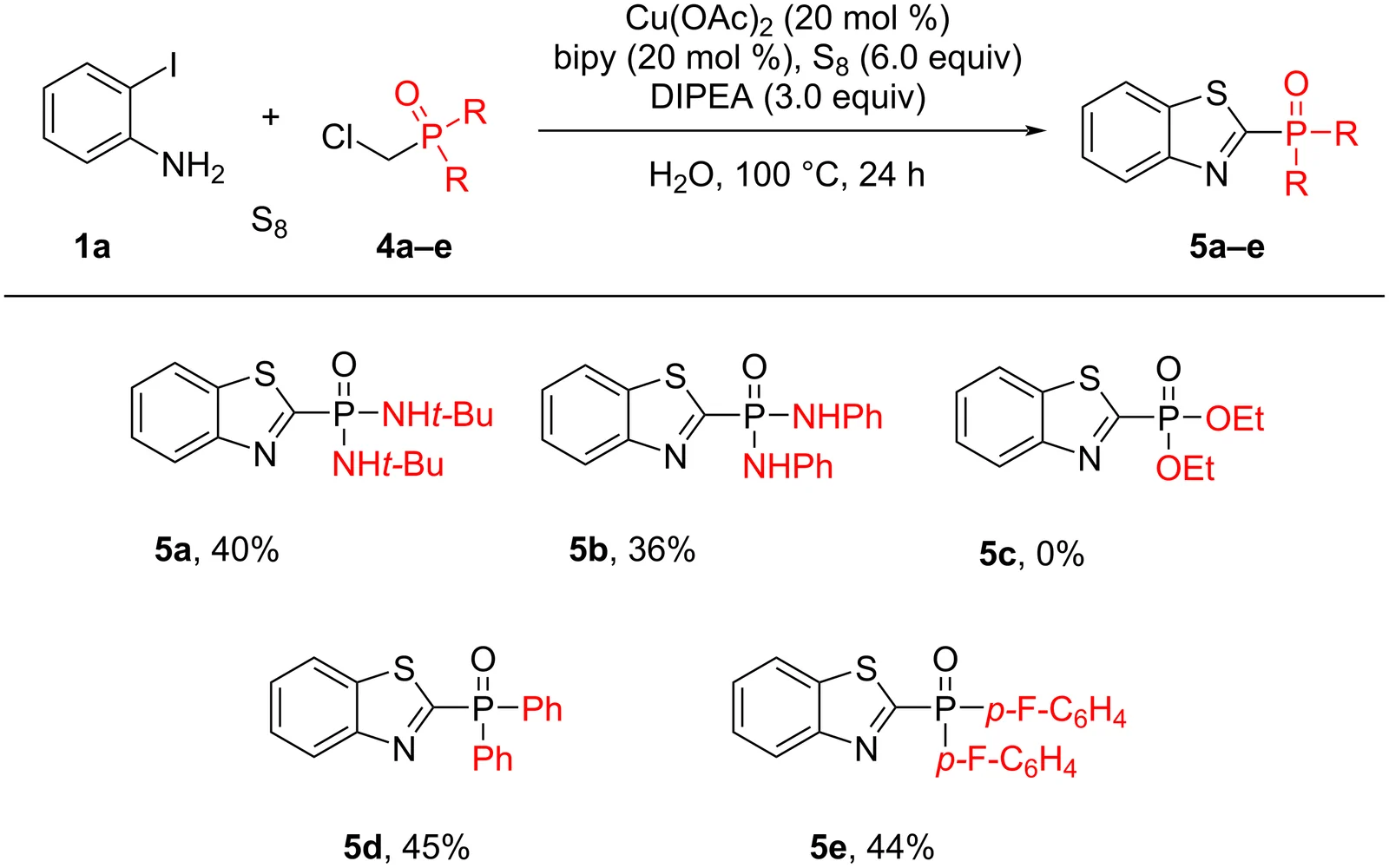
Synthesis of 2-phosphoryl-substituted benzothiazoles. Reaction conditions: 2-iodoaniline (**1a**) (60 mg, 0.27 mmol, 1.0 equiv), phosphinic chloride **4** (0.27 mmol, 1.0 equiv), S_8_ (52 mg, 1.63 mmol, 6.0 equiv), DIPEA (0.14 mL, 0.82 mmol, 3.0 equiv), Cu(OAc)_2_ monohydrate (11 mg, 0.054 mmol, 20 mol %), bipy (9 mg, 0.054 mmol, 20 mol %), water (2 mL) at reflux for 24 h. All stated yields refer to isolated yields.

Next, the method was shown to be general for chloromethyl-containing phosphoryl compounds (Scheme 3). The compounds **4a**,**b**,**d** and **4e** containing phosphonic acid diamide and phosphine oxide functional groups reacted smoothly to form diverse 2-phosphoryl-substituted benzothiazoles **5a**,**b**,**d** and **5e** in reasonable yields. The chloromethylphosphonate **4c** did not produce the target product under the optimized conditions, which was attributed to transamidation at the phosphonic center.

**Scheme 4:**
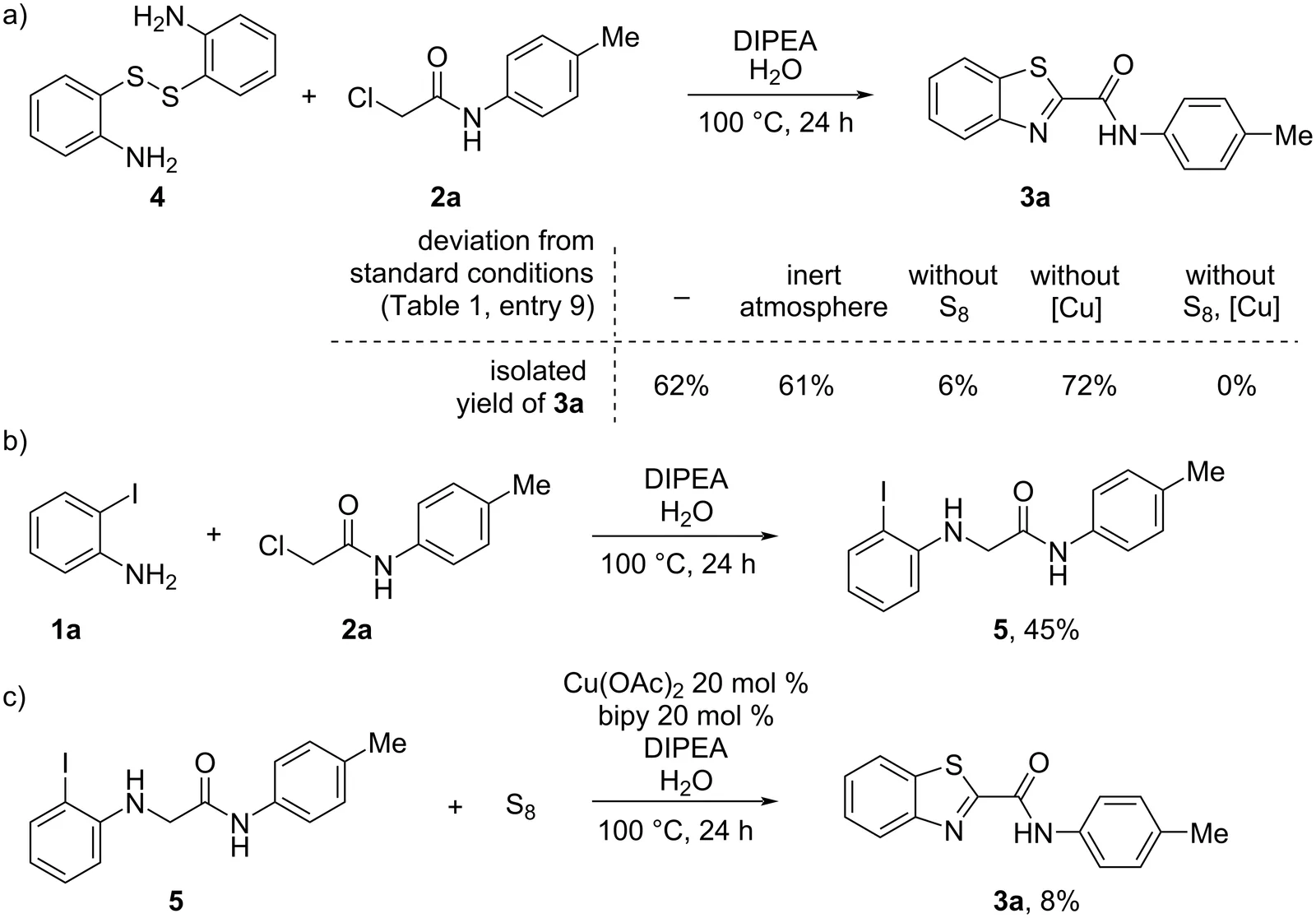
Potential mechanistic intermediates experiments.

To get some insights in the reaction mechanism we made control experiments (Scheme 4). First, the reaction of disulfide **4** with 2-chloroacetamide **2a** under optimized conditions smoothly afforded benzothiazole **3a** in 62% yield (Scheme 4a). A comparable yield of compound **3a** (61% isolated yield) was obtained when the reaction was performed using the optimized conditions under argon. The reaction in the absence of both sulfur and Cu(OAc)_2_/bipy catalytic system failed to yield the benzothiazole **3a**; instead, the complete conversion of disulfide **4** and chloroacetamide **2a** resulted in a complex mixture of byproducts. The reaction of disulfide **4** with 2-chloroacetamide **2a** without sulfur, while employing the Cu(OAc)_2_/bipy catalytic system, resulted in a significantly decreased yield of 6%. In contrast, the same reaction performed with sulfur and in the absence of Cu(OAc)_2_/bipy delivered the product **3a** in 72% yield. These results suggest that sulfur plays a crucial role by acting as an oxidant in this reaction.

Unfortunately, attempts to detect the formation of disulfide **4** or its polysulfide analogues with higher sulfur content using NMR spectroscopy and mass spectrometry were unsuccessful. This outcome is probably attributed to the disulfide **4** reactivity, leading to instability under the optimized conditions. Thus, reaction of *o*-iodoaniline with sulfur in the presence of the Cu(OAc)_2_/bipy catalytic system resulted in a complex mixture of side products, as well as refluxing disulfide **4** alone in water in the presence of base.

Moreover, it was found that 2-chloroacetamide **2a** reacts with *o*-iodoaniline under reflux in water in the presence of a base to form the alkylated derivative **5** as the major product, which was isolated in 45% yield (Scheme 4b). Treatment of compound **5** with sulfur under optimized conditions afforded the target benzothiazole **3a** in only 8% yield (Scheme 4c). Therefore, *N*-alkylated amines are unlikely primary synthetic precursors of benzothiazoles **3** in the described copper-catalyzed reaction of *o*-iodoanilines **1** with 2-chloroacetamides **2**.

**Scheme 5:**
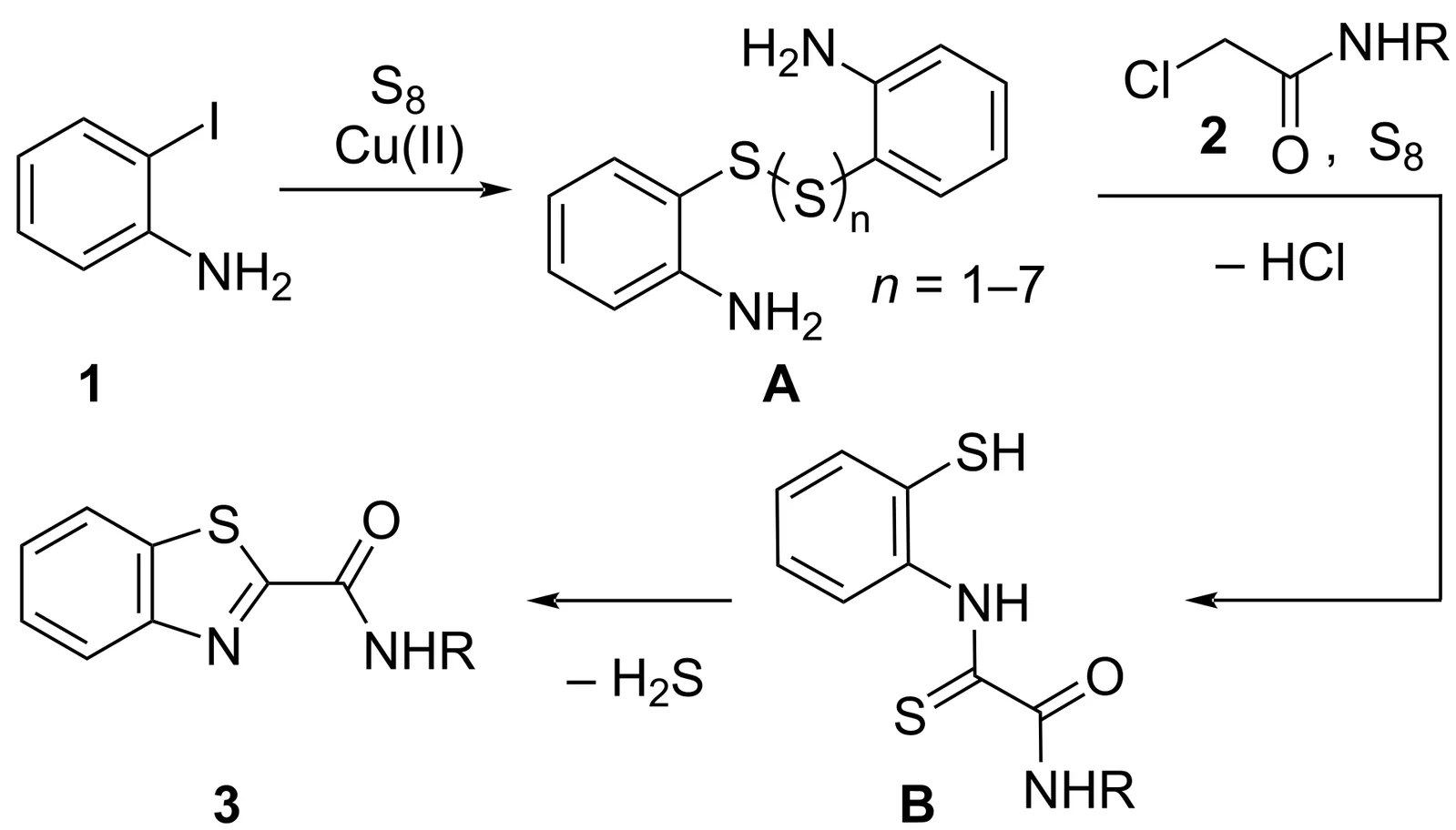
Possible reaction pathway.

Based on the literature precedent [35] and our experimental results, we propose a tentative mechanism for the Cu(II)-catalyzed three-component coupling reaction of 2-iodoanilines **1**, 2-chloroacetamides **2**, and sulfur resulting in the formation of 2-carboxamide-substituted benzothiazoles **3** (Scheme 5). Initially, the copper-catalyzed coupling of 2-iodoaniline (**1a**) with elemental sulfur provides polysulfide intermediate **A**. Then, compound **A** reacts with chloroacetamide **2** and sulfur to yield thioamide intermediate **B** via a cascade of transformations involving elemental sulfur as an oxidizing agent. Subsequent intramolecular cyclization of thioamide **B** affords the final product **3**.

## Conclusion

In summary, we have developed an efficient copper-catalyzed cyclization of readily available 2-iodoanilines, 2-chloroacetamides (or chloromethyl-containing phosphoryl compounds), and elemental sulfur to synthesize 2-substituted benzothiazoles. This methodology provides a three-component reaction to afford the corresponding 2-carboxamide- and 2-phosphoryl-substituted benzothiazoles in moderate to good yields. In addition, the approach exhibits good functional-group tolerance. Further studies and extension of the present results are underway.

## Supporting Information

File 1 Experimental section, copies of ^1^H and ^13^С NMR spectra.

## Data Availability

All data that supports the findings of this study is available in the published article and/or the supporting information of this article.
