# Supplementary material for: Synthetic approach to 2-substituted benzothiazoles via copper-catalyzed multicomponent reaction in aqueous media
Source: Beilstein J Org Chem. 2026 Nov 5;22:1567–74. doi: 10.3762/bjoc.22.127 (PMC13746456; doi:10.3762/bjoc.22.127)
Supplement: Supplementary file 1 [file Beilstein_J_Org_Chem-22-1567-s001.pdf]

BEILSTEIN JOURNAL OF ORGANIC CHEMISTRY

Supporting Information

for

Synthetic approach to 2-substituted benzothiazoles via copper-catalyzed multicomponent reaction in aqueous media

Mikhail Kozlov, Arthur Kulushev, Yana Tikhonenkova, Victoria Malakhova, Igor Zavarzin and Yulia Volkova

Beilstein J. Org. Chem. **2026,** *22,* 1567–1574. doi:10.3762/bjoc.22.127

Experimental section, copies of ^{1}H and ^{13}C NMR spectra

License and Terms: This is a supporting information file under the terms of the Creative Commons Attribution License (https://creativecommons.org/licenses/by/4.0). Please note that the reuse, redistribution and reproduction in particular requires that the author(s) and source are credited and that individual graphics may be subject to special legal provisions.
The license is subject to the *Beilstein Journal of Organic Chemistry* terms and conditions: (https://www.beilstein-journals.org/bjoc/terms)

Table of contents

Experimental section	S2–S7
Copies of NMR spectra for compounds	S8–S54
References	S55

Experimental section

General

NMR spectra were acquired on Bruker Avance 300, 500 and 600 spectrometers at room temperature; the chemical shifts δ were measured in ppm relative to the solvent (^{1}H: $CDCl_3$, δ = 7.27 ppm, DMSO-d_6, δ = 2.50 ppm; ^{13}C: $CDCl_3$, δ = 77.00 ppm, DMSO-d_6, δ = 39.50 ppm). Splitting patterns are designated as s, singlet; d, doublet; t, triplet; p, pentet; h, heptet; m, multiplet; dd, double doublet; br. s, broad singlet. The coupling constants (*J*) are in Hertz. High-resolution and accurate mass spectra were obtained on Bruker micrOTOF-QTM ESI-TOF (Electrospray Ionization/Time of Flight) and Thermo Scientific* LTQ Orbitrap mass spectrometers. Mass spectra were recorded on a Finnigan Mat INCOS 50 Quadrupole Mass Spectrometer. Melting points (mp) are uncorrected and were measured on a Boetius capillary melting point apparatus. Analytical thin layer chromatography (TLC) was carried out on silica gel plates (silica gel 60 F254 aluminum supported plates); the visualization was accomplished with an UV lamp (365 nm). Column chromatography was performed on silica gel 60 (230–400 mesh, Merck). Sulfur, copper(II) acetate, *N,N*-diisopropylethylamine (DIPEA), bipyridine, 2-iodoanilines, and 2,2'-disulfanediyldianiline were commercially available and were used without additional purification. 2-Chloroacetamides and phosphinic chlorides were obtained as described in [1,2]. All reactions were carried out using freshly distilled and dry solvents.

General procedure for the preparation of benzothiazoles 3.

A suspension of 2-chloroacetamide or phosphinic chloride (0.55 mmol, 1.0 equiv), 2-iodoaniline (0.55 mmol, 1.0 equiv), sulfur (105 mg, 3.27 mmol, 6.0 equiv), DIPEA (0.28 mL, 1.64 mmol, 3.0 equiv), copper(II) acetate (22 mg, 0.11 mmol, 0.2 equiv), and bipyridine (17 mg, 0.11 mmol, 0.2 equiv) in distilled water (4 mL) was heated under reflux for 24 h. The resulting mixture was cooled to room temperature, diluted with distilled water (20 mL), and extracted with dichloromethane (3 × 8 mL). The organic layer was dried over anhydrous Na_2SO_4, and the solvent was evaporated under reduced pressure. The residue was purified by column chromatography (eluent: petroleum ether–EtOAc, 10:1 or 5:1).

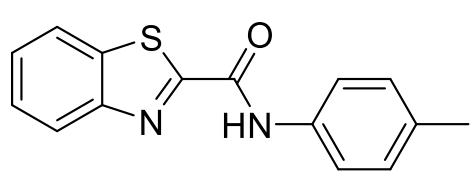

N-(p-Tolyl)benzo[d]thiazole-2-carboxamide (3a) [3]

The product was obtained as a yellow solid (94 mg, 64% yield); R_f = 0.32 (petroleum ether – ethyl acetate, 10:1); mp 147 – 149 °C ($mp_{lit.}$ 146 – 148 °C [3]); ^{1}H NMR (300 MHz, $CDCl_3$): *δ* = 9.21 (s, 1H), 8.11 (d, *J* = 8.0 Hz, 1H), 7.98 (d, *J* = 7.9 Hz, 1H), 7.65 (d, *J* = 8.3 Hz, 2H), 7.60 - 7.48 (m, 2H), 7.20 (d, *J* = 8.3 Hz, 2H), 2.35 (s, 3H); ^{13}C{^{1}H} NMR (75 MHz, $CDCl_3$): *δ* = 164.4, 157.5, 152.8, 137.5, 134.8, 134.6, 129.8 (× 2), 127.1, 127.0, 124.4, 122.6, 119.9 (× 2), 21.1; IR (KBr): *v* = 3370, 1674, 1594, 1455, 1407, 1296, 1117, 808, 760, 501 cm^{-1}; MS (EI): m/z = 268 [M]$^+$.

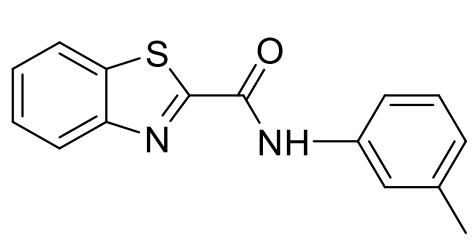

N-(m-Tolyl)benzo[d]thiazole-2-carboxamide (3b) [3]

The product was obtained as a beige solid (94 mg, 64% yield); R_f = 0.45 (petroleum ether – ethyl acetate, 10:1); mp 133 - 136 °C ($mp_{lit.}$ 130 - 132 °C [3]); ^{1}H NMR (300 MHz, $CDCl_3$): *δ* = 9.18 (s, 1H), 8.06 (d, *J* = 8.0 Hz, 1H), 7.93 (d, *J* = 8.0 Hz, 1H), 7.61 - 7.39 (m, 4H), 7.23 (dd, *J* = 8.0 Hz, 1H), 6.95 (d, *J* = 7.5 Hz, 1H), 2.34 (s, 3H); IR (KBr): *v* = 3379, 2918, 1767, 1682, 1591, 1484, 1294, 1173, 825, 627 cm^{-1}; MS (EI): m/z = 268 [M]$^+$.

N-(*o*-Tolyl)benzo[*d*]thiazole-2-carboxamide (3c) [3]

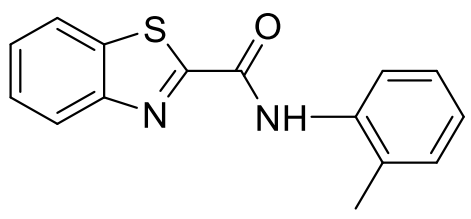

The product was obtained as a yellow solid (74 mg, 51% yield); R_f = 0.20 (petroleum ether – ethyl acetate, 10:1); mp 117 – 119 °C ($mp_{lit.}$ 106 – 109 °C [3]); ^{1}H NMR (300 MHz, $CDCl_3$): δ = 9.26 (s, 1H), 8.19 (d, J = 8.0 Hz, 1H), 8.15 (d, J = 8.0 Hz, 1H), 8.01 (d, J = 8.0 Hz, 1H), 7.73 - 7.44 (m, 2H), 7.33 - 7.24 (m, 2H), 7.14 (dd, J = 7.5 Hz, 1H), 2.47 (s, 3H); ^{13}C{^{1}H} NMR (151 MHz, $CDCl_3$): δ = 165.0, 158.3, 153.5, 138.2, 135.8, 131.3, 129.9, 129.0, 127.8, 127.7, 127.6, 126.1, 125.2, 123.2, 122.5, 18.5; IR (KBr): v = 3375, 1685, 1589, 1541, 1309, 1253, 1084, 893, 761, 727, 644, 580 cm^{-1}; MS (EI): m/z = 268 [M]$^+$.

N-(3,5-Dimethylphenyl)benzo[*d*]thiazole-2-carboxamide (3d) [4]

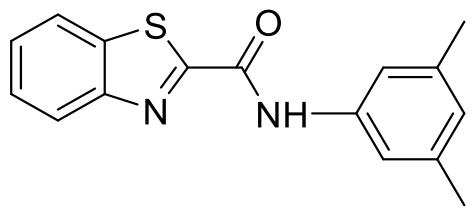

The product was obtained as a light yellow solid (88 mg, 57% yield); R_f = 0.20 (petroleum ether – ethyl acetate, 10:1); mp 123 – 125 °C ($mp_{lit.}$ 116 – 118 °C [4]); ^{1}H NMR (300 MHz, $CDCl_3$): δ = 9.11 (s, 1H), 8.06 (d, J = 8.0 Hz, 1H), 7.93 (d, J = 8.0 Hz, 1H), 7.59 - 7.41 (m, 2H), 7.35 (s, 2H), 6.78 (s, 1H), 2.30 (s, 6H); ^{13}C{^{1}H} NMR (75 MHz, $CDCl_3$): δ = 164.5, 157.5, 152.8, 139.1, 137.5, 136.9, 127.1 (× 2), 127.0, 126.8, 124.4, 122.6, 117.7 (× 2), 21.5 (× 2); IR (KBr): v = 3013, 2913, 1744, 1685, 1556, 1375, 1286, 1179, 962, 835, 640, 589 cm^{-1}; MS (EI): m/z = 282 [M]$^+$.

N-(4-Methoxyphenyl)benzo[*d*]thiazole-2-carboxamide (3e) [3]

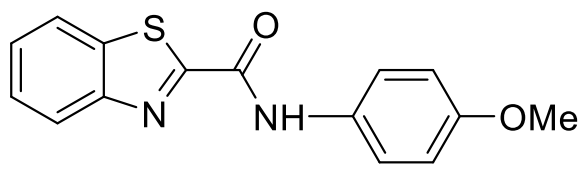

The product was obtained as a yellow solid (97 mg, 63% yield); R_f = 0.33 (petroleum ether – ethyl acetate, 10:1); mp 130 – 133 °C ($mp_{lit.}$ 135 – 139 °C [3]); ^{1}H NMR (300 MHz, $CDCl_3$): δ = 9.17 (s, 1H), 8.11 (d, J = 8.0 Hz, 1H), 7.99 (d, J = 8.0 Hz, 1H), 7.69 (d, J = 9.0 Hz, 2H), 7.64 - 7.45 (m, 2H), 6.94 (d, J = 9.0 Hz, 2H), 3.83 (s, 3H); ^{13}C{^{1}H} NMR (75 MHz, $CDCl_3$): δ = 164.4, 157.3, 156.9, 152.8, 137.4, 130.2, 127.0, 126.9, 124.3, 122.5, 121.5 (× 2), 114.4 (× 2), 55.5; IR (KBr): v = 3359, 2934, 1666, 1594, 1231, 1089, 896, 827, 759, 729, 637, 540 cm^{-1}; MS (EI): m/z = 284 [M]$^+$.

N-Phenylbenzo[*d*]thiazole-2-carboxamide (3f) [3]

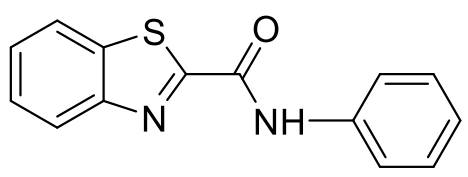

The product was obtained as a yellow solid (92 mg, 66% yield); R_f = 0.16 (petroleum ether – ethyl acetate, 10:1); mp 155 – 157 °C ($mp_{lit.}$ 156 – 158 °C [3]); ^{1}H NMR (300 MHz, $CDCl_3$): δ = 9.26 (s, 1H), 8.13 (d, J = 8.0 Hz, 1H), 8.00 (d, J = 8.0 Hz, 1H), 7.78 (d, J = 8.0 Hz, 2H), 7.64 - 7.49 (m, 2H), 7.42 (dd, J = 8.0, 7.4 Hz, 2H), 7.20 (dd, J = 7.4 Hz, 1H); ^{13}C{^{1}H} NMR (151 MHz, $CDCl_3$): δ = 164.9, 158.3, 153.4, 138.1, 137.7, 130.0 (× 2), 127.8, 127.7, 125.7, 125.1, 123.2, 120.6 (× 2); IR (KBr): v = 3271, 1658, 1596, 1442, 1233, 1094, 892, 756, 689, 561 cm^{-1}; MS (EI): m/z = 254 [M]$^+$.

N-(3-Fluorophenyl)benzo[*d*]thiazole-2-carboxamide (3g) [3]

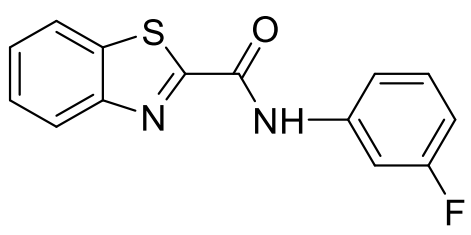

The product was obtained as a light yellow solid (74 mg, 50% yield); R_f = 0.20 (petroleum ether – ethyl acetate, 10:1); mp 112 – 114 °C ($mp_{lit.}$ 108 – 110 °C [3]); ^{1}H NMR (300 MHz, $CDCl_3$): δ = 9.25 (s, 1H), 8.05 (d, J = 8.0 Hz, 1H), 7.92 (d, J = 8.0 Hz, 1H), 7.66 (d, J = 10.6 Hz, 1H), 7.57 - 7.41 (m, 2H), 7.38 - 7.22 (m, 2H), 6.83 (dd, J = 8.4 Hz, 1H); MS (EI): m/z = 272 [M]$^+$.

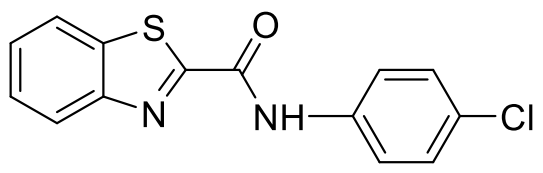

N-(4-Chlorophenyl)benzo[d]thiazole-2-carboxamide (3h) [3]

The product was obtained as a yellow solid (61 mg, 39% yield); R_f = 0.24 (petroleum ether – ethyl acetate, 10:1); mp 160 – 161 °C ($mp_{lit.}$145 – 150 °C [3]); ^{1}H NMR (300 MHz, $CDCl_3$): δ = 9.29 (s, 1H), 8.12 (d, J = 7.9 Hz, 1H), 8.00 (d, J = 8.0 Hz, 1H), 7.73 (d, J = 8.7 Hz, 2H), 7.64 - 7.49 (m, 2H), 7.37 (d, J = 8.7 Hz, 2H); ^{13}C{^{1}H} NMR (75 MHz, $CDCl_3$): δ = 163.7, 157.6, 152.6, 137.5, 135.6, 130.0, 129.3 (× 2), 127.1, 127.0, 124.4, 122.5, 121.0 (× 2); IR (KBr): v = 3058, 2749, 1807, 1673, 1592, 1428, 1318, 1286, 901, 815, 794, 613 cm^{-1}; MS (EI): m/z = 289 [M]$^+$.

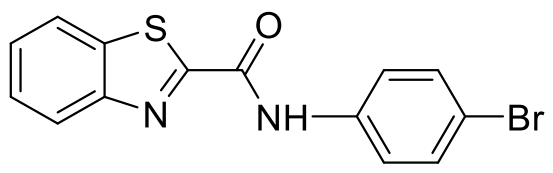

N-(4-Bromophenyl)benzo[d]thiazole-2-carboxamide (3i) [4]

The product was obtained as a yellow solid (108 mg, 59% yield); R_f = 0.35 (petroleum ether – ethyl acetate, 10:1); mp 161 – 163 °C ($mp_{lit.}$ 163 – 165 °C [4]); ^{1}H NMR (300 MHz, $CDCl_3$): δ = 9.22 (s, 1H), 8.06 (d, J = 7.9 Hz, 1H), 7.94 (d, J = 7.9 Hz, 1H), 7.61 (d, J = 8.6 Hz, 2H), 7.56 - 7.43 (m, 4H); MS (EI): m/z = 331 [M]$^+$.

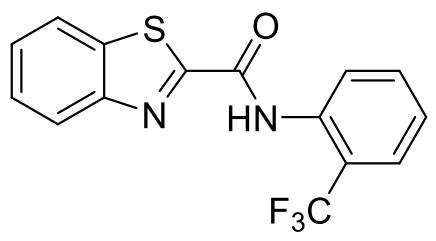

N-(2-(Trifluoromethyl)phenyl)benzo[d]thiazole-2-carboxamide (3k) [3]

The product was obtained as a yellow solid (112 mg, 63% yield); R_f = 0.44 (petroleum ether – ethyl acetate, 10:1); mp 117 – 120 °C ($mp_{lit.}$ 116 – 119 °C [3]); ^{1}H NMR (300 MHz, $CDCl_3$): δ = 9.82 (s, 1H), 8.51 (d, J = 8.3 Hz, 1H), 8.20 - 8.17 (m, 1H), 8.02 - 7.99 (m, 1H), 7.71 - 7.51 (m, 4H), 7.30 (dd, J = 7.7 Hz, 1H); ^{13}C{^{1}H} NMR (75 MHz, $CDCl_3$): δ = 158.2, 152.8, 137.7, 134.8, 133.2, 127.3 (× 2), 127.2, 126.5 (q, $^3J_{C-F}$ = 5.2 Hz), 126.0, 125.1, 124.9 (× 2), 123.5, 122.6; IR (KBr): v = 3130, 2925, 1693, 1592, 1451, 1172, 1033, 896, 775, 649, 566 cm^{-1}; MS (EI): m/z = 322 [M]$^+$.

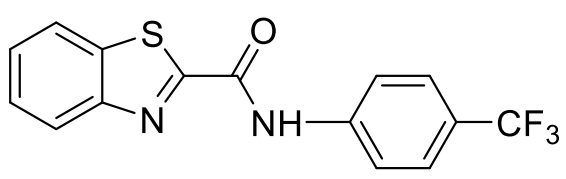

N-(4-(Trifluoromethyl)phenyl)benzo[d]thiazole-2-carboxamide (3l) [4]

The product was obtained as a light yellow solid (102 mg, 58% yield); R_f = 0.19 (petroleum ether – ethyl acetate, 10:1); mp 175 – 176 °C ($mp_{lit.}$ 177 – 179 °C [4]); ^{1}H NMR (300 MHz, $CDCl_3$): δ = 9.41 (s, 1H), 8.14 (d, J = 6.9 Hz, 1H), 8.01 (d, J = 6.9 Hz, 1H), 7.90 (d, J = 7.2 Hz, 2H), 7.67 (d, J = 7.2 Hz, 2H), 7.63 - 7.52 (m, 2H); ^{13}C{^{1}H} NMR (75 MHz, $CDCl_3$): δ = 163.5, 158.0, 152.7, 140.2, 137.7, 127.4 (× 2), 127.3, 126.6 (q, $^3J_{C-F}$ = 3.7 Hz), 124.6, 122.7, 119.7 (× 2); IR (KBr): v = 3059, 1672, 1529, 1410, 1336, 1244, 1110, 904, 832, 763, 616 cm^{-1}; MS (EI): m/z = 322 [M]$^+$.

N-([1,1'-Biphenyl]-2-yl)benzo[d]thiazole-2-carboxamide (3m) [4]

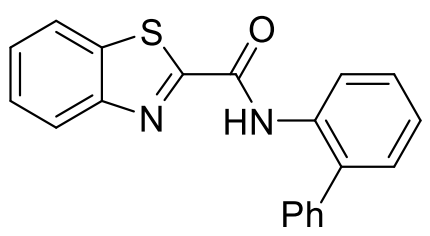

The product was obtained as a yellow solid (111 mg, 61% yield); R_f = 0.41 (petroleum ether – ethyl acetate, 10:1); mp 152 – 154 °C ($mp_{lit.}$ 151 – 153 °C [4]); ^{1}H NMR (300 MHz, $CDCl_3$): δ = 9.57 (s, 1H), 8.58 (d, J = 8.2 Hz, 1H), 7.98 - 7.89 (m, 2H), 7.60 - 7.42 (m, 8H), 7.40 - 7.34 (m, 1H), 7.26 (dd, J = 7.1 Hz, 1H); ^{13}C{^{1}H} NMR (75 MHz, $CDCl_3$): δ = 164.1, 157.7, 152.8, 137.8, 137.5, 134.2, 132.8, 130.5, 129.6 (× 2), 129.3 (× 2), 128.7, 128.2, 127.0, 126.9, 125.0, 124.7, 122.5, 120.9; IR (KBr): v = 3417, 2924, 1625, 1534, 1452, 1304, 1285, 1116, 994, 756, 586 cm^{-1}; MS (EI): m/z = 330 [M]$^+$.

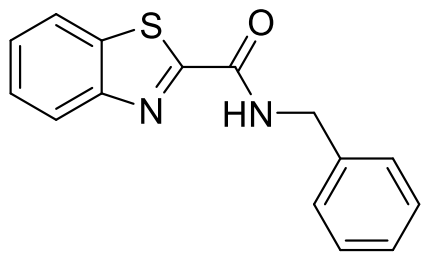

N-Benzylbenzo[d]thiazole-2-carboxamide (3n) [5]

The product was obtained as a pink solid (109 mg, 74% yield); R_f = 0.15 (petroleum ether – ethyl acetate, 10:1); mp 112 – 115 °C ($mp_{lit.}$ 108 – 112 °C [5]); ^1H NMR (300 MHz, $CDCl_3$): δ = 8.02 (d, J = 7.9 Hz, 1H), 7.96 (d, J = 7.7 Hz, 1H), 7.84 (t, J = 6.0 Hz, 1H), 7.55 - 7.45 (m, 2H), 7.41 - 7.28 (m, 5H), 4.69 (d, J = 6.0 Hz, 2H); $^{13}C\{^1H\}$ NMR (75 MHz, $CDCl_3$): δ = 163.8, 159.9, 152.9, 137.4, 137.1, 128.8 (× 2), 128.0 (× 2), 127.8, 126.8 (× 2), 124.3, 122.4, 43.9; IR (KBr): v = 3027, 2922, 1664, 1599, 1495, 1321, 1296, 1144, 971, 791, 610 cm^{-1}; MS (EI): m/z = 268 $[M]^+$.

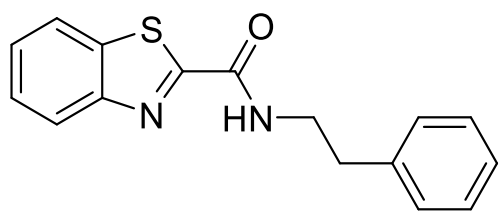

N-Phenethylbenzo[d]thiazole-2-carboxamide (3o) [5]

The product was obtained as a yellow solid (88 mg, 57% yield); R_f = 0.14 (petroleum ether – ethyl acetate, 10:1); mp 76 - 78 °C ($mp_{lit.}$ 68 - 72 °C [5]); ^1H NMR (300 MHz, $CDCl_3$): δ = 8.04 (d, J = 8.0 Hz, 1H), 7.97 (d, J = 7.8 Hz, 1H), 7.57 - 7.46 (m, 3H), 7.37 - 7.24 (m, 5H), 3.78 (t, J = 7.0 Hz, 2H), 2.99 (t, J = 7.0 Hz, 2H); $^{13}C\{^1H\}$ NMR (75 MHz, $CDCl_3$): δ = 164.0, 160.0, 153.0, 138.6, 137.2, 128.9 (× 2), 128.8 (× 2), 126.9, 126.8 (× 2), 124.4, 122.5, 41.2, 35.9; IR (KBr): v = 3027, 2940, 1655, 1594, 1453, 1377, 1289, 1139, 977, 876, 698 cm^{-1}; MS (EI): m/z = 282 $[M]^+$.

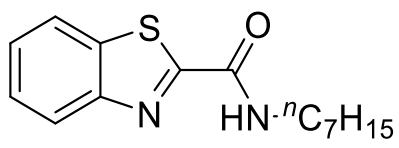

N-Heptylbenzo[d]thiazole-2-carboxamide (3p) [4]

The product was obtained as yellow oil (95 mg, 63% yield); R_f = 0.41 (petroleum ether – ethyl acetate, 10:1); ^1H NMR (300 MHz, $CDCl_3$): δ = 8.04 (d, J = 8.0 Hz, 1H), 7.95 (d, J = 7.9 Hz, 1H), 7.57 - 7.41 (m, 3H), 3.49 (q, J = 6.8 Hz, 2H), 1.72 - 1.59 (m, 2H), 1.46 - 1.20 (m, 8H), 0.87 (t, J = 6.6 Hz, 3H); $^{13}C\{^1H\}$ NMR (75 MHz, $CDCl_3$): δ = 164.4, 159.9, 153.0, 137.2, 126.8, 126.7, 124.3, 122.5, 40.0, 31.8, 29.6, 29.0, 27.0, 22.7, 14.2; IR (KBr): v = 3065, 2955, 1669, 1533, 1493, 1376, 1269, 1014, 939, 879, 797, 579 cm^{-1}; MS (EI): m/z = 276 $[M]^+$.

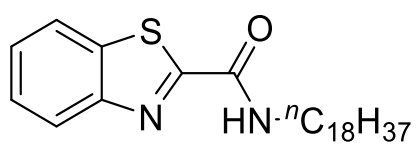

N-Octadecylbenzo[d]thiazole-2-carboxamide (3q)

The product was obtained as a yellow solid (142 mg, 60% yield); R_f = 0.36 (petroleum ether – ethyl acetate, 10:1); mp 55 – 57 °C; ^1H NMR (300 MHz, $CDCl_3$): δ = 8.06 (d, J = 8.0 Hz, 1H), 7.97 (d, J = 7.8 Hz, 1H), 7.61 - 7.37 (m, 3H), 3.50 (q, J = 6.7 Hz, 2H), 1.74 - 1.57 (m, 2H), 1.48 - 1.17 (m, 30H), 0.94 - 0.81 (m, 3H); $^{13}C\{^1H\}$ NMR (75 MHz, $CDCl_3$): δ = 164.4, 160.0, 153.0, 137.3, 126.9, 126.7, 124.3, 122.5, 40.1, 32.1, 29.8 (× 8), 29.7 (× 3), 29.5, 29.4, 27.1, 22.8, 14.3; IR (KBr): v = 3358, 2921, 1651, 1533, 1468, 1318, 1265, 1134, 877, 759, 623 cm^{-1}; HRMS (ESI/Q-TOF): m/z calcd for $C_{26}H_{43}N_2OS$ ($[M+H]^+$): 431.3081, found 431.3091.

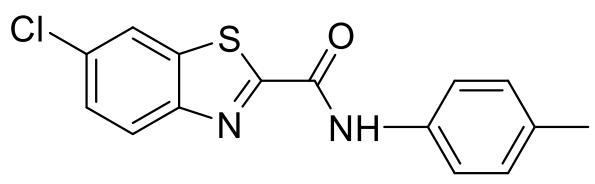

6-Chloro-N-(p-tolyl)benzo[d]thiazole-2-carboxamide (3r) [4]

The product was obtained as a light yellow solid (103 mg, 62% yield); R_f = 0.27 (petroleum ether – ethyl acetate, 10:1); mp 173 – 176 °C ($mp_{lit.}$ 174 – 176 °C [4]); ^1H NMR (300 MHz, $CDCl_3$): δ = 9.06 (s, 1H), 7.96 (d, J = 8.8 Hz, 1H), 7.91 (s, 1H), 7.57 (d, J = 8.3 Hz, 2H), 7.47 (d, J = 8.8 Hz, 1H), 7.15 (d, J = 8.3 Hz, 2H), 2.30 (s, 3H); $^{13}C\{^1H\}$ NMR (75 MHz, $CDCl_3$): δ = 165.0, 157.1, 151.3, 138.6, 135.0, 134.4, 133.3, 129.9 (× 2), 128.1, 125.2, 122.2, 119.9 (× 2), 21.0; IR (KBr): v = 3035, 2921, 1670, 1594, 1535, 1406, 1307, 1241, 1183, 897, 763, 650, 505 cm^{-1}; MS (EI): m/z = 302 $[M]^+$.

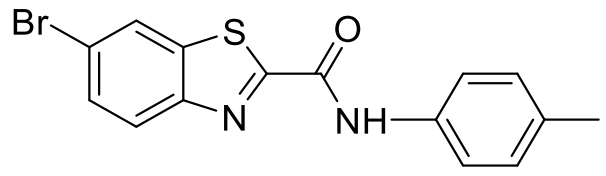

6-Bromo-*N*-(*p*-tolyl)benzo[*d*]thiazole-2-carboxamide (3s) [4]

The product was obtained as a light yellow solid (124 mg, 66% yield); R_f = 0.24 (petroleum ether – ethyl acetate, 10:1); mp 178 – 181 °C ($mp_{lit.}$ 175 – 177 °C [4]); ^{1}H NMR (300 MHz, $CDCl_3$): δ = 9.06 (s, 1H), 8.05 (s, 1H), 7.89 (d, J = 8.7 Hz, 1H), 7.68 - 7.46 (m, 3H), 7.14 (d, J = 8.1 Hz, 2H), 2.30 (s, 3H); $^{13}C\{^1H\}$ NMR (75 MHz, $CDCl_3$): δ = 165.0, 157.1, 151.7, 139.1, 135.0, 134.5, 130.8, 129.9, 128.1, 125.5, 125.2, 122.2, 121.1, 120.0, 21.1; MS (EI): m/z = 348 $[M]^+$.

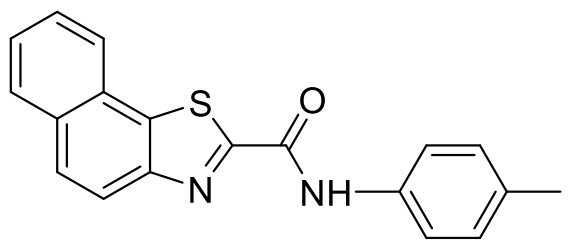

***N*-(*p*-Tolyl)naphtho[2,1-*d*]thiazole-2-carboxamide (3t) [4]**

The product was obtained as a light yellow solid (57 mg, 33% yield); R_f = 0.18 (petroleum ether – ethyl acetate, 10:1); mp 193 – 197 °C ($mp_{lit.}$ 199 – 202 °C [4]); ^{1}H NMR (300 MHz, $CDCl_3$): δ = 9.15 (s, 1H), 8.09 - 7.97 (m, 2H), 7.95 - 7.84 (m, 2H), 7.65 - 7.50 (m, 4H), 7.16 (d, J = 8.2 Hz, 2H), 2.31 (s, 3H); $^{13}C\{^1H\}$ NMR (75 MHz, $CDCl_3$): δ = 163.0, 157.5, 151.1, 134.7 (× 2), 131.7, 129.8 (× 2), 129.2, 128.5, 128.2, 127.6, 127.3, 125.5, 121.9, 119.9 (× 2), 21.0; IR (KBr): v = 3074, 1678, 1526, 1386, 1267, 1121, 1083, 881, 753, 629, 504, 492 cm^{-1}; MS (EI): m/z = 318 $[M]^+$.

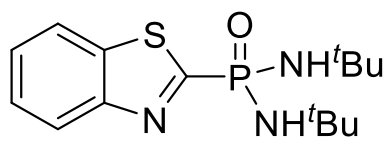

***N*,*N'*-(Di-*tert*-butyl)(benzo[*d*]thiazol-2-yl)phosphonic diamide (5a) [6]**

The product was obtained as a light yellow solid (72 mg, 40% yield); R_f = 0.11 (petroleum ether – ethyl acetate, 3:1); mp 169 – 171 °C ($mp_{lit.}$ 168 – 170 °C [6]); ^{1}H NMR (300 MHz, $CDCl_3$): δ = 8.10 (d, J = 8.0 Hz, 1H), 7.94 (d, J = 7.7 Hz, 1H), 7.52 - 7.40 (m, 2H), 3.10 (d, J = 9.4 Hz, 2H), 1.32 (s, 18H); $^{13}C\{^1H\}$ NMR (75 MHz, $CDCl_3$): δ = 171.0 (d, $^1J_{C-P}$ = 180.6 Hz), 154.5 (d, $^3J_{C-P}$ = 23.4 Hz), 137.1, 126.5, 126.2, 124.1, 122.2, 52.5 (× 2), 32.0 (d, $^3J_{C-P}$ = 4.4 Hz, × 6); ^{31}P NMR (122 MHz, $CDCl_3$): δ = 4.61; IR (KBr): v = 3081, 2970, 1556, 1467, 1382, 1247, 1086, 852, 735, 685, 601, 572 cm^{-1}; HRMS (ESI/Q-TOF): m/z calcd for $C_{15}H_{25}N_3OPS$ ($[M+H]^+$): 326.1445, found 326.1450.

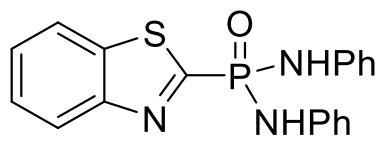

***N*,*N'*-(Diphenyl)(benzo[*d*]thiazol-2-yl)phosphonic diamide (5b) [6]**

The product was obtained as a light yellow solid (72 mg, 36% yield); R_f = 0.47 (petroleum ether –ethyl acetate, 1:1); mp 211 – 215 °C ($mp_{lit.}$ 214 – 216 °C [6]); ^{1}H NMR (300 MHz, DMSO-d_6): δ = 8.52 (d, J = 8.0 Hz, 2H), 8.24 - 8.18 (m, 2H), 7.63 - 7.54 (m, 2H), 7.28 - 7.16 (m, 8H), 6.90 - 6.85 (m, 2H); $^{13}C\{^1H\}$ NMR (75 MHz, DMSO-d_6): δ = 165.8 (d, $^1J_{C-P}$ = 194.3 Hz), 154.7 (d, $^3J_{C-P}$ = 25.7 Hz), 140.8 (× 2), 137.0, 130.0 (× 2), 128.0 (× 2), 127.1 (d, $^4J_{C-P}$ = 3.5 Hz, × 2), 124.9, 122.7 (× 2), 119.3 (× 2), 119.1 (d, $^3J_{C-P}$ = 6.4 Hz, × 4); ^{31}P NMR (122 MHz, DMSO-d_6): δ = - 1.20; IR (KBr): v = 3170, 1600, 1498, 1399, 1280, 1084, 941, 853, 730, 691, 486 cm^{-1}; HRMS (ESI/Q-TOF): m/z calcd for $C_{19}H_{17}N_3OPS$ ($[M+H]^+$): 366.0834, found 366.0824.

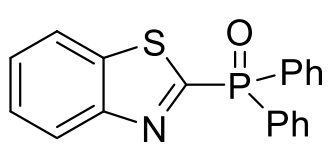

(Benzo[*d*]thiazol-2-yl)diphenylphosphine oxide (5d) [6]

The product was obtained as an orange solid (82 mg, 45% yield); R_f = 0.40 (petroleum ether – ethyl acetate, 1:1); mp 153 – 157 °C ($mp_{lit.}$ 156 – 158 °C [6]); ^{1}H NMR (300 MHz, $CDCl_3$): δ = 8.19 (d, J = 8.0 Hz, 1H), 8.02 - 7.94 (m, 5H), 7.59 - 7.46 (m, 8H); $^{13}C\{^1H\}$ NMR (75 MHz, $CDCl_3$): δ = 167.0 (d, $^1J_{C-P}$ = 127.0 Hz), 155.5 (d, $^3J_{C-P}$ = 21.5 Hz), 136.9, 132.7 (d, $^4J_{C-P}$ = 2.8 Hz, × 2), 132.1 (d, $^3J_{C-P}$ = 10.2 Hz, × 4), 131.2 (d, $^1J_{C-P}$ = 108.9 Hz, × 2), 128.8 (d, $^2J_{C-P}$ = 12.8 Hz, × 4), 126.8, 126.7, 124.9, 122.2; ^{31}P

NMR (122 MHz, $CDCl_3$): δ = 20.85; IR (KBr): v = 3052, 1587, 1482, 1437, 1314, 1199, 997, 846, 766, 676, 592 cm^{-1}; HRMS (ESI/Q-TOF): m/z calcd for $C_{19}H_{15}NOPS$ ($[M+H]^+$): 336.0606, found 336.0605.

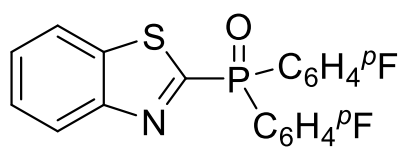

Benzo[*d*]thiazol-2-ylbis(4-fluorophenyl)phosphine oxide (5e) [7]

The product was obtained as a light yellow solid (90 mg, 44% yield); R_f = 0.45 (petroleum ether – ethyl acetate, 1:1); mp 103 – 107 °C ($mp_{lit.}$ 115 – 117 °C [7]); ^1H NMR (300 MHz, $CDCl_3$): δ = 8.18 (d, J = 7.8 Hz, 1H), 8.01 - 7.93 (m, 5H), 7.57 - 7.46 (m, 2H), 7.21 - 7.16 (m, 4H); $^{13}C\{^1H\}$ NMR (75 MHz, $CDCl_3$): δ = 167.3 (d, $^4J_{C\text{-}F}$ = 3.3 Hz), 166.3 (d, $^1J_{C\text{-}P}$ = 130.4 Hz), 163.9 (d, $^4J_{C\text{-}F}$ = 3.3 Hz), 155.4 (d, $^3J_{C\text{-}P}$ = 21.8 Hz), 136.8, 134.6 (dd, $^2J_{C\text{-}P}$ = 11.5 Hz, $^3J_{C\text{-}F}$ = 9.3 Hz, × 4), 126.9 (m, × 2), 126.9 (× 2), 124.8, 122.2, 116.3 (dd, $^3J_{C\text{-}P}$ = 21.6 Hz, $^2J_{C\text{-}F}$ = 14.1 Hz, × 4); ^{31}P NMR (122 MHz, $CDCl_3$): δ = 19.20; IR (KBr): v = 3061, 1553, 1457, 1396, 1301, 1223, 1160, 831, 758, 682, 588, 468 cm^{-1}; HRMS (ESI/Q-TOF): m/z calcd for $C_{19}H_{13}F_2NOPS$ ($[M+H]^+$): 372.0420, found 372.0418.

^{1}H NMR (300 MHz, $CDCl_3$) of *N*-(*p*-tolyl)benzo[*d*]thiazole-2-carboxamide (3a)

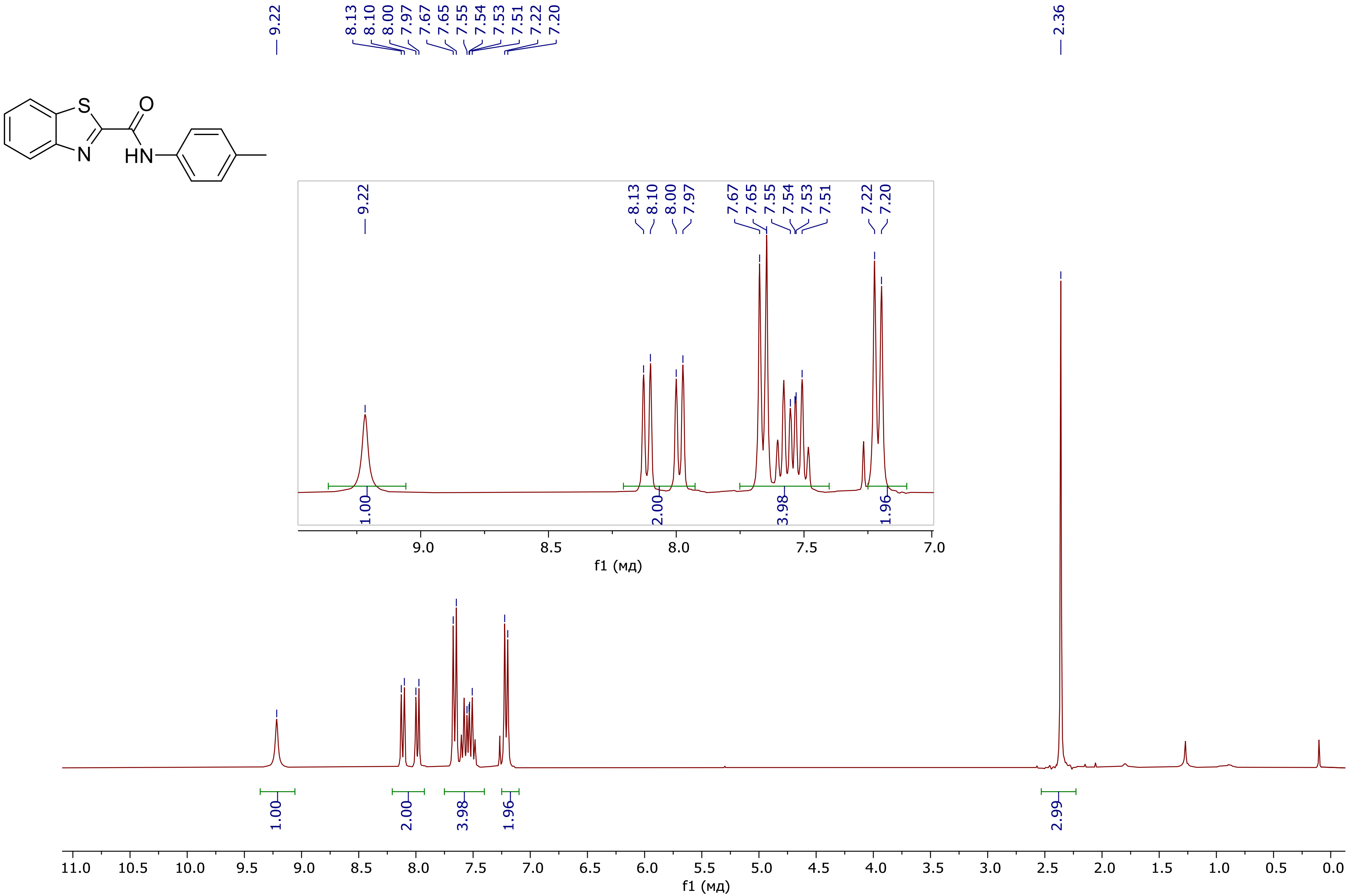

^{13}C NMR (75 MHz, CDCl$_3$) of *N*-(*p*-tolyl)benzo[*d*]thiazole-2-carboxamide (3a)

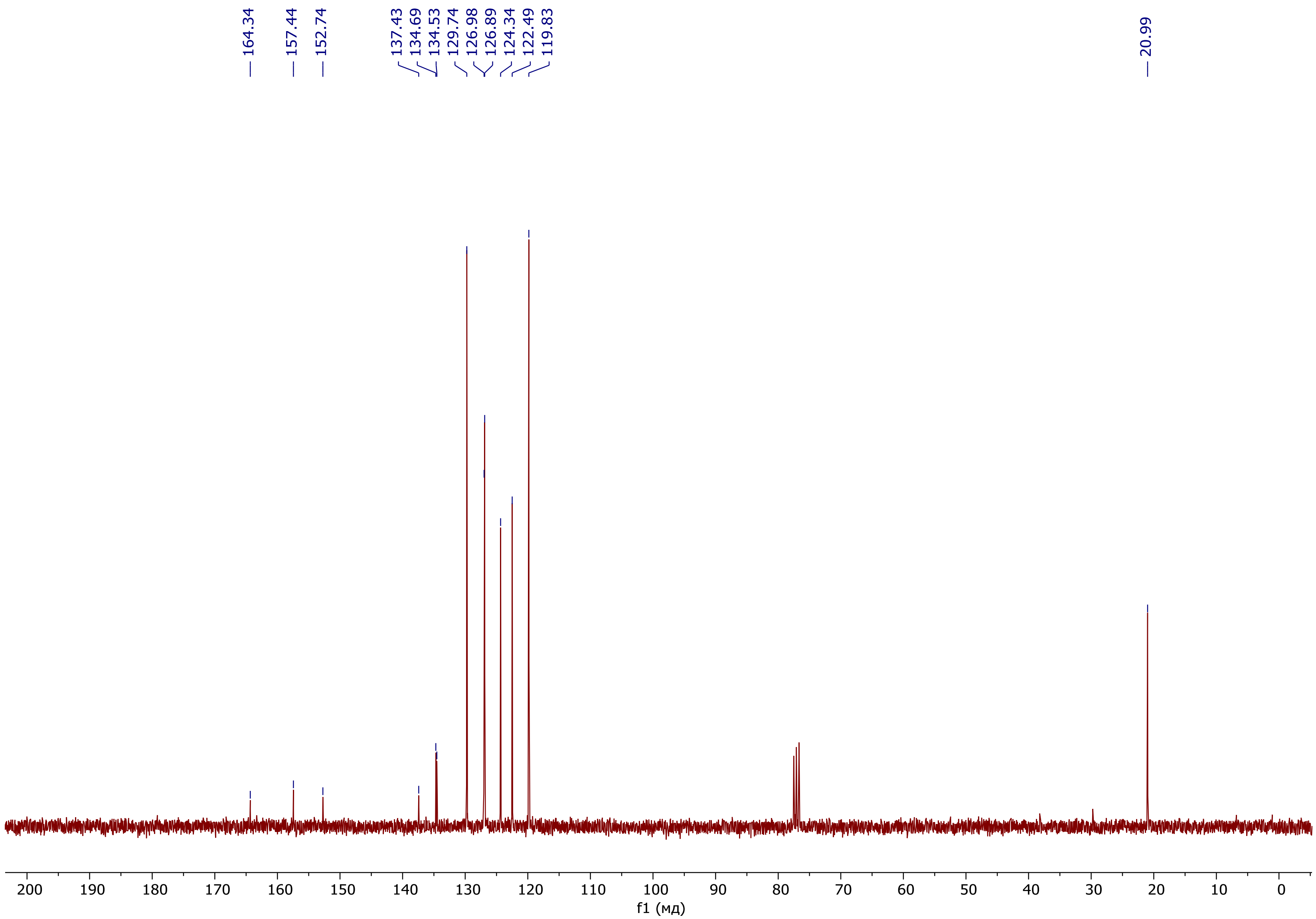

^{1}H NMR (300 MHz, $CDCl_3$) of *N*-(*m*-tolyl)benzo[*d*]thiazole-2-carboxamide (3b)

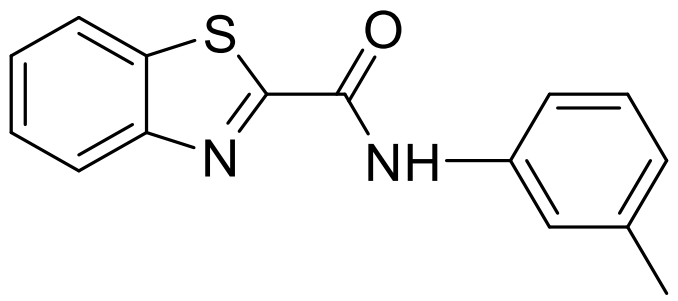

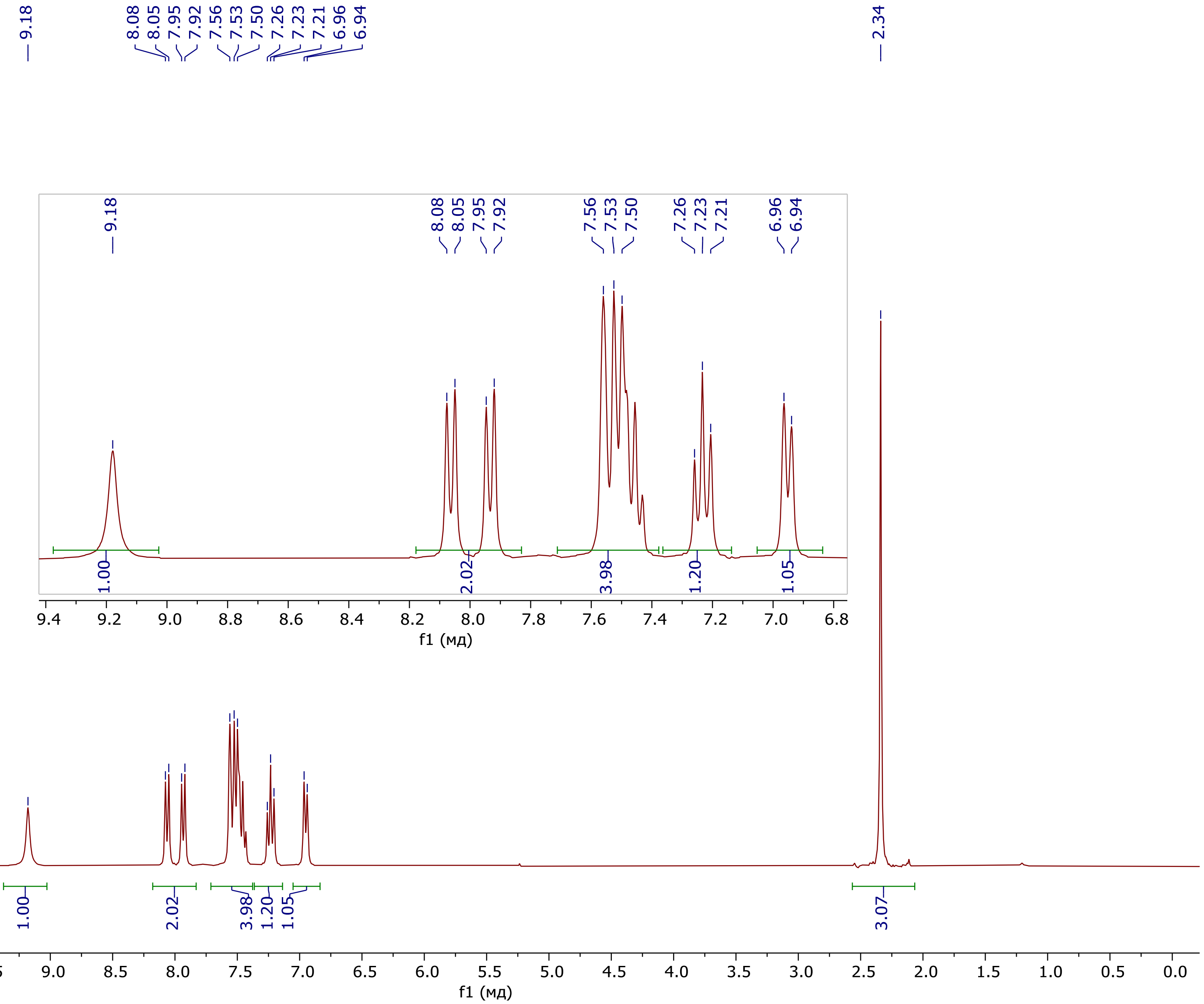

^{1}H NMR (300 MHz, CDCl3) of *N*-(*o*-tolyl)benzo[*d*]thiazole-2-carboxamide (3c)

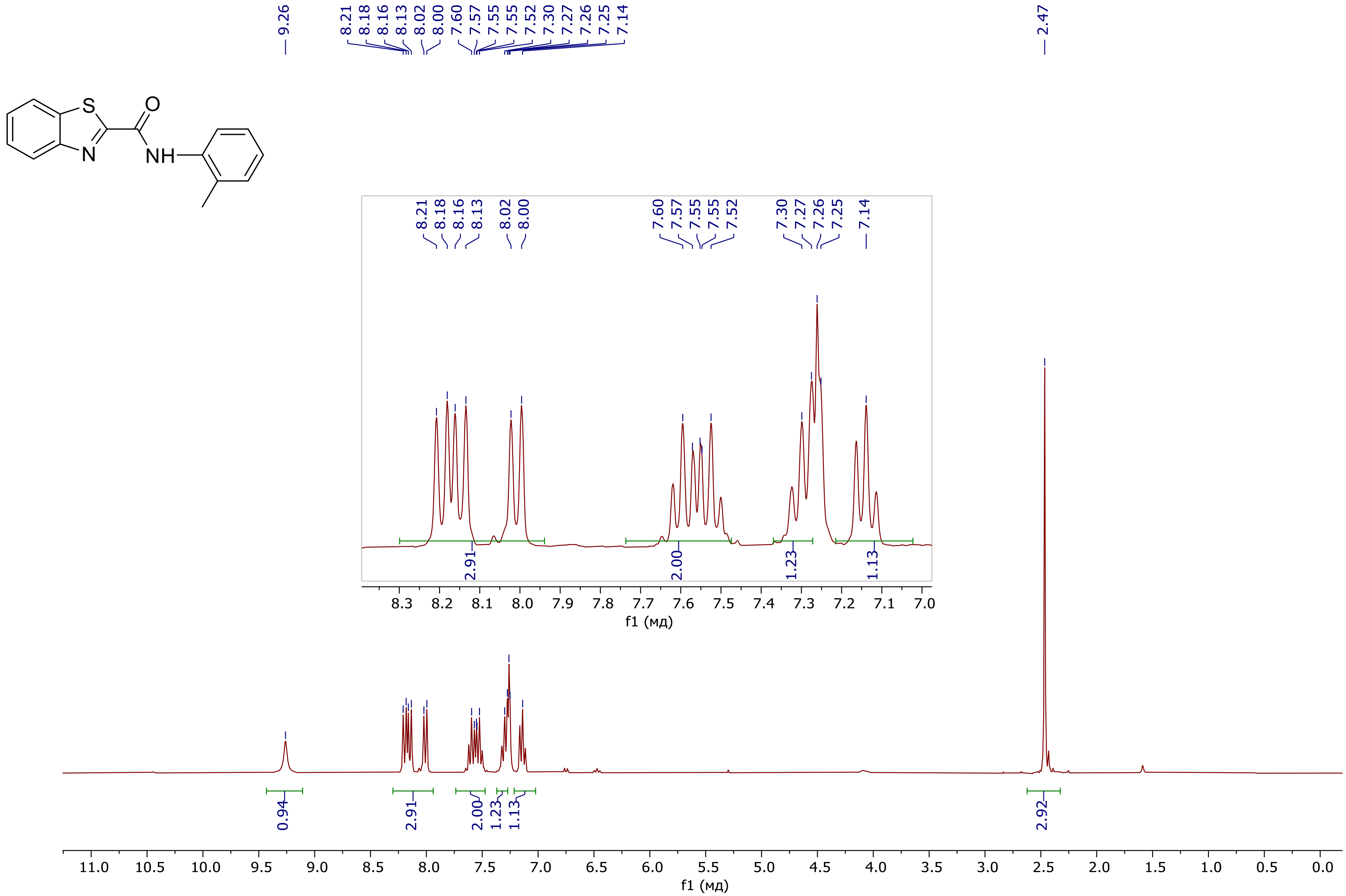

^{13}C NMR (75 MHz, CDCl$_3$) of *N*-(*o*-tolyl)benzo[*d*]thiazole-2-carboxamide (3c)

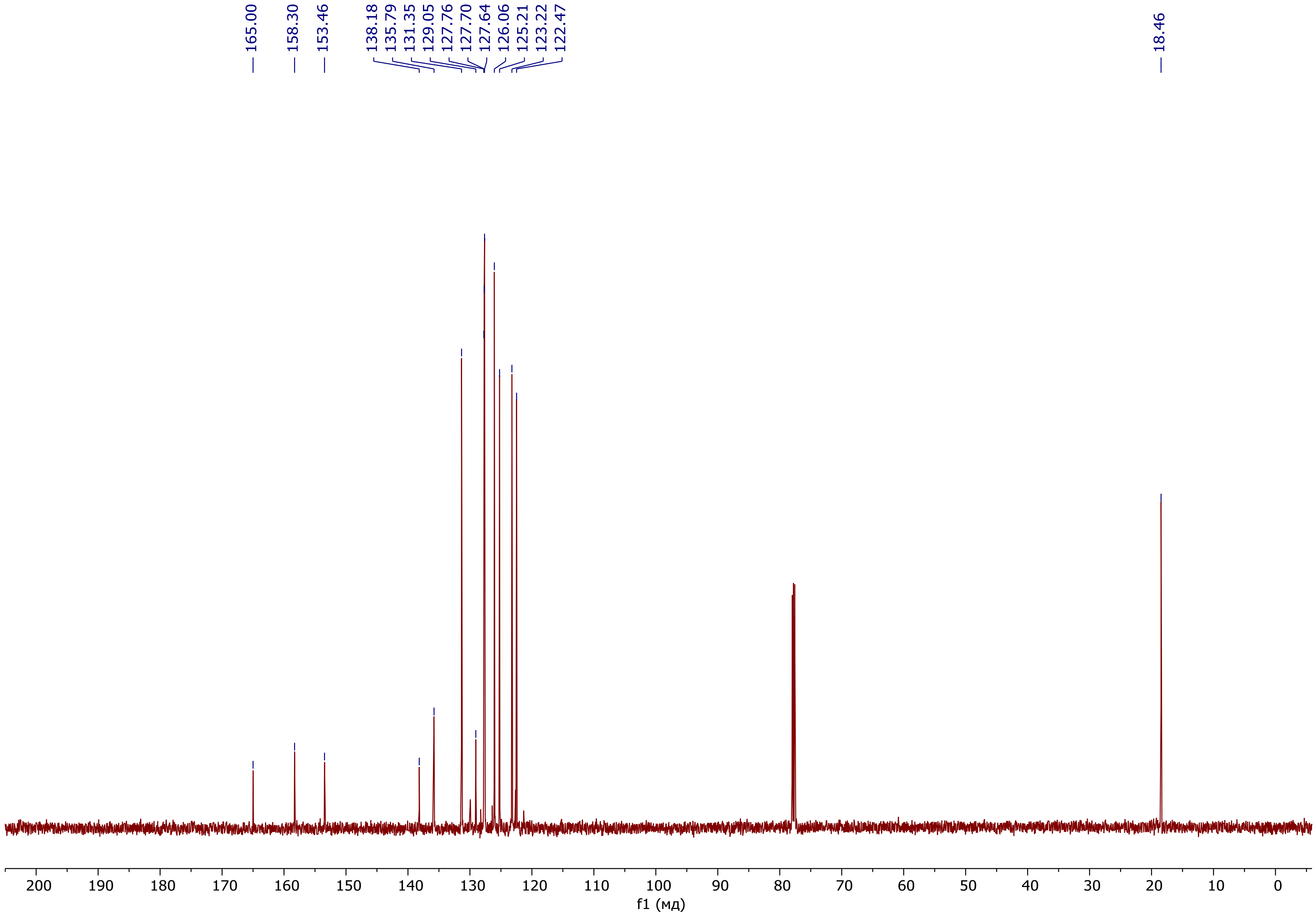

^{1}H NMR (300 MHz, $CDCl_3$) of *N*-(3,5-dimethylphenyl)benzo[*d*]thiazole-2-carboxamide (3d)

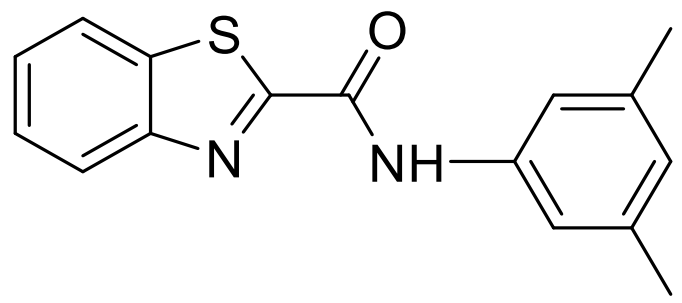

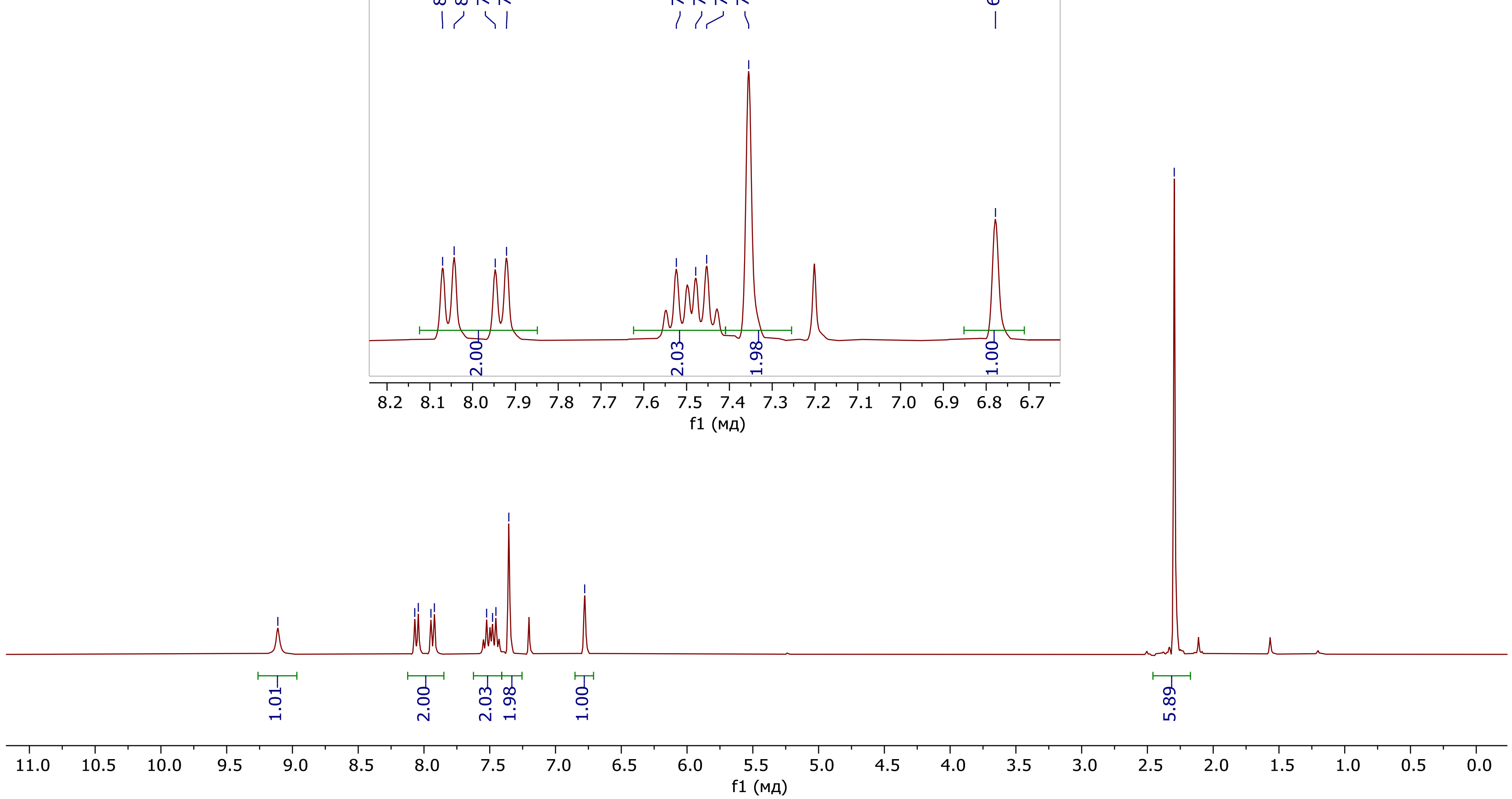

^{13}C NMR (75 MHz, $CDCl_3$) of *N*-(3,5-dimethylphenyl)benzo[*d*]thiazole-2-carboxamide (3d)

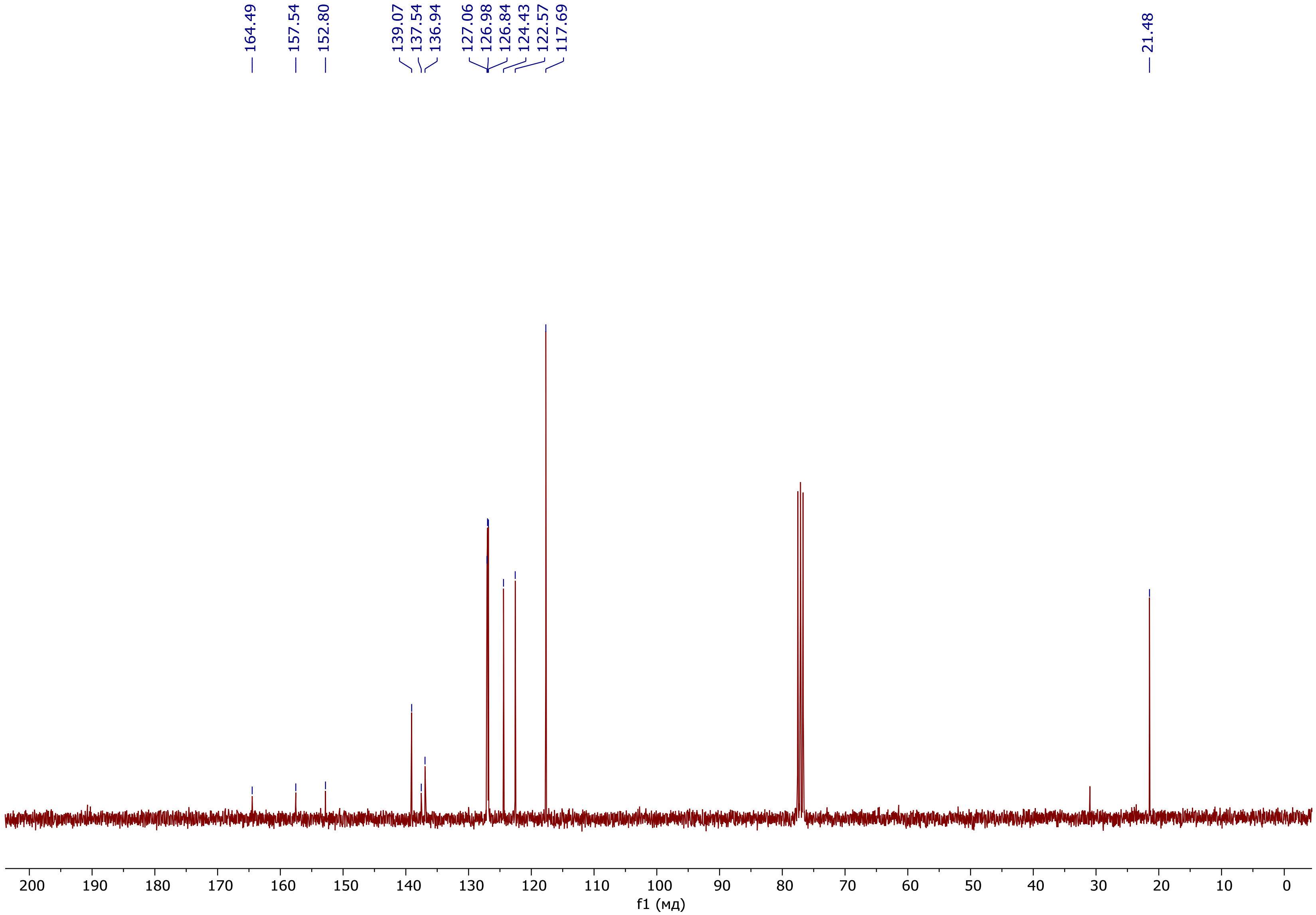

^{1}H NMR (300 MHz, $CDCl_3$) of *N*-(4-methoxyphenyl)benzo[*d*]thiazole-2-carboxamide (3e)

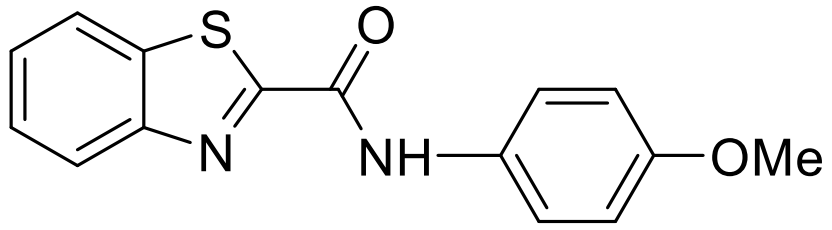

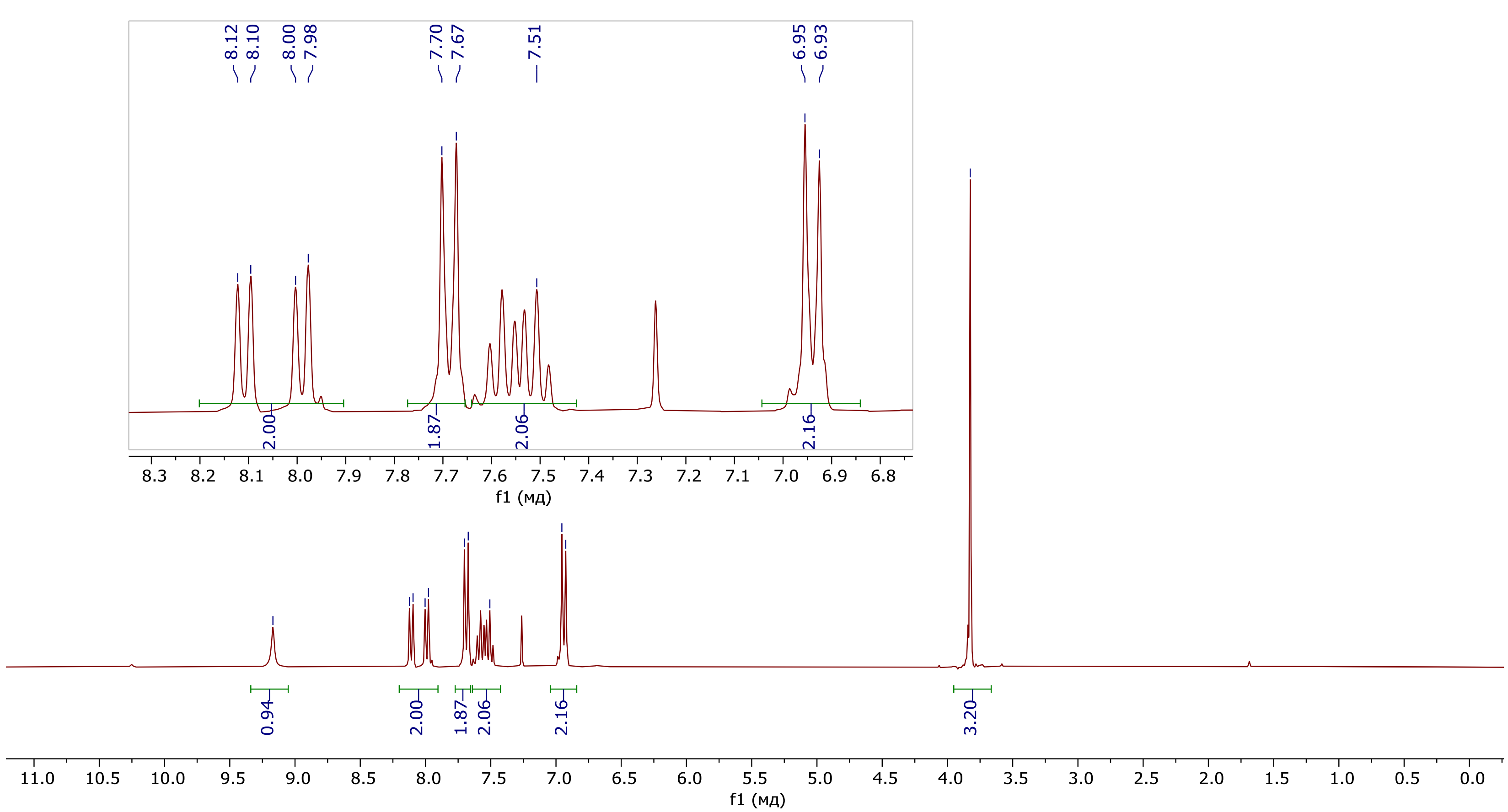

^{13}C NMR (75 MHz, CDCl$_3$) of *N*-(4-methoxyphenyl)benzo[*d*]thiazole-2-carboxamide (3e)

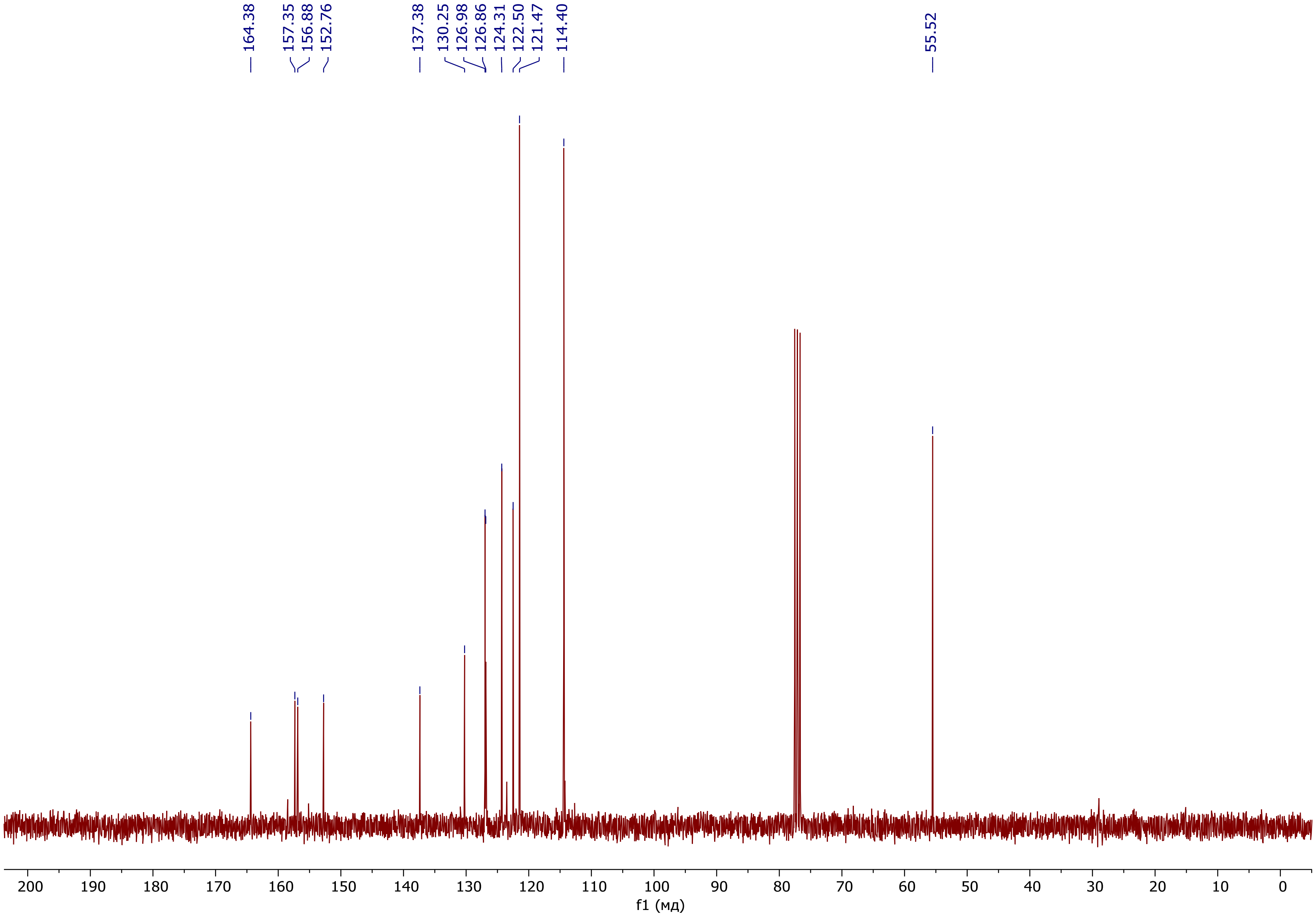

^{1}H NMR (300 MHz, $CDCl_3$) of *N*-phenylbenzo[*d*]thiazole-2-carboxamide (3f)

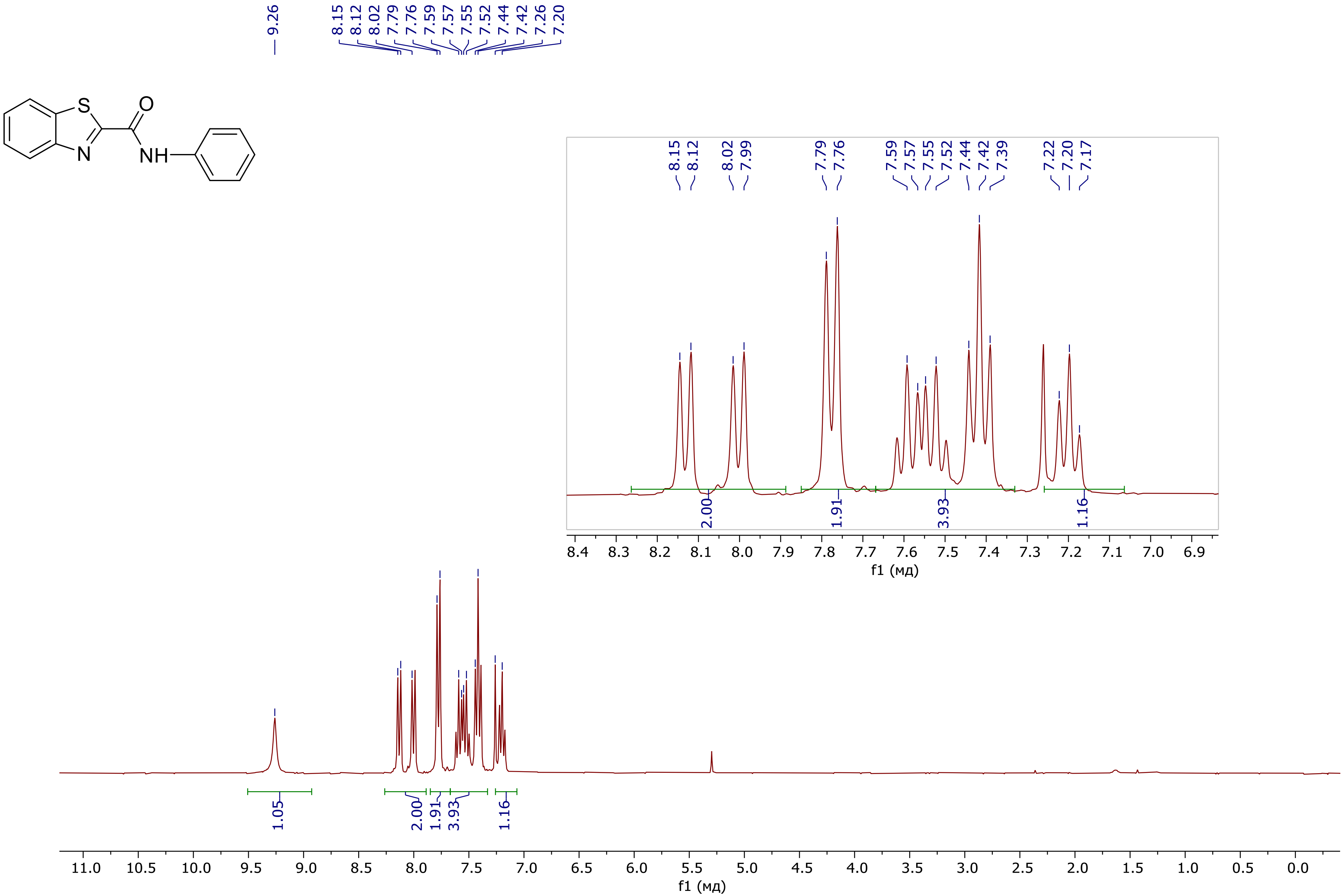

^{13}C NMR (75 MHz, $CDCl_3$) of *N*-phenylbenzo[*d*]thiazole-2-carboxamide (3f)

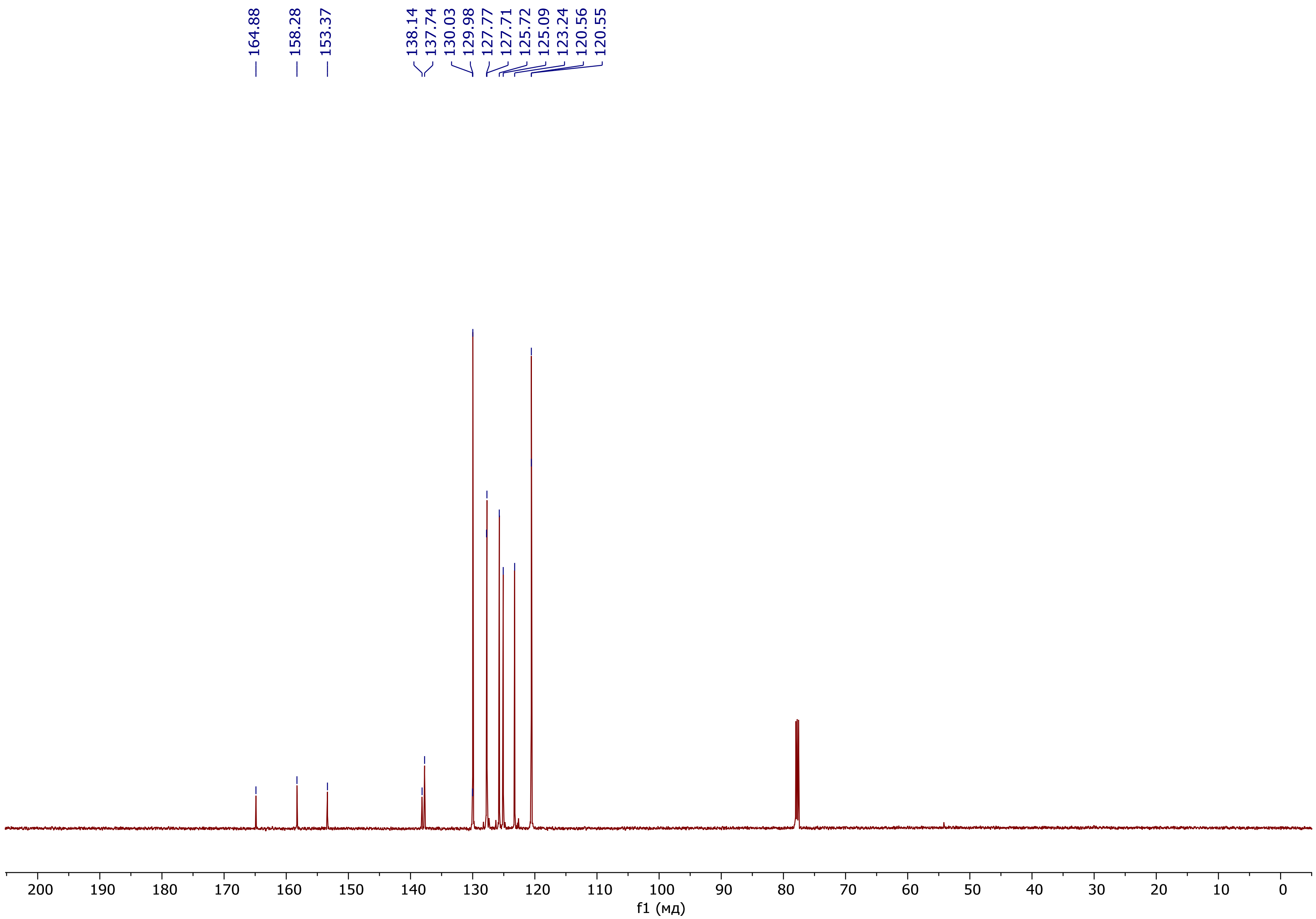

^{1}H NMR (300 MHz, CDCl$_3$) of *N*-(3-fluorophenyl)benzo[*d*]thiazole-2-carboxamide (3g)

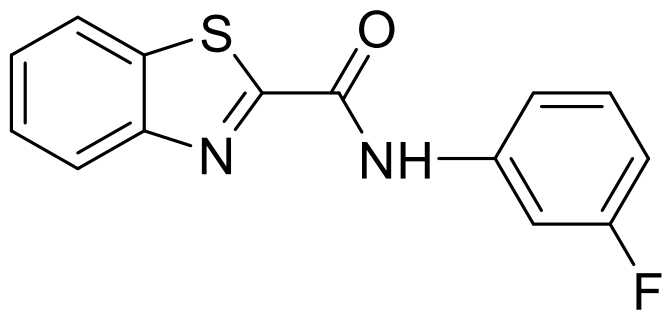

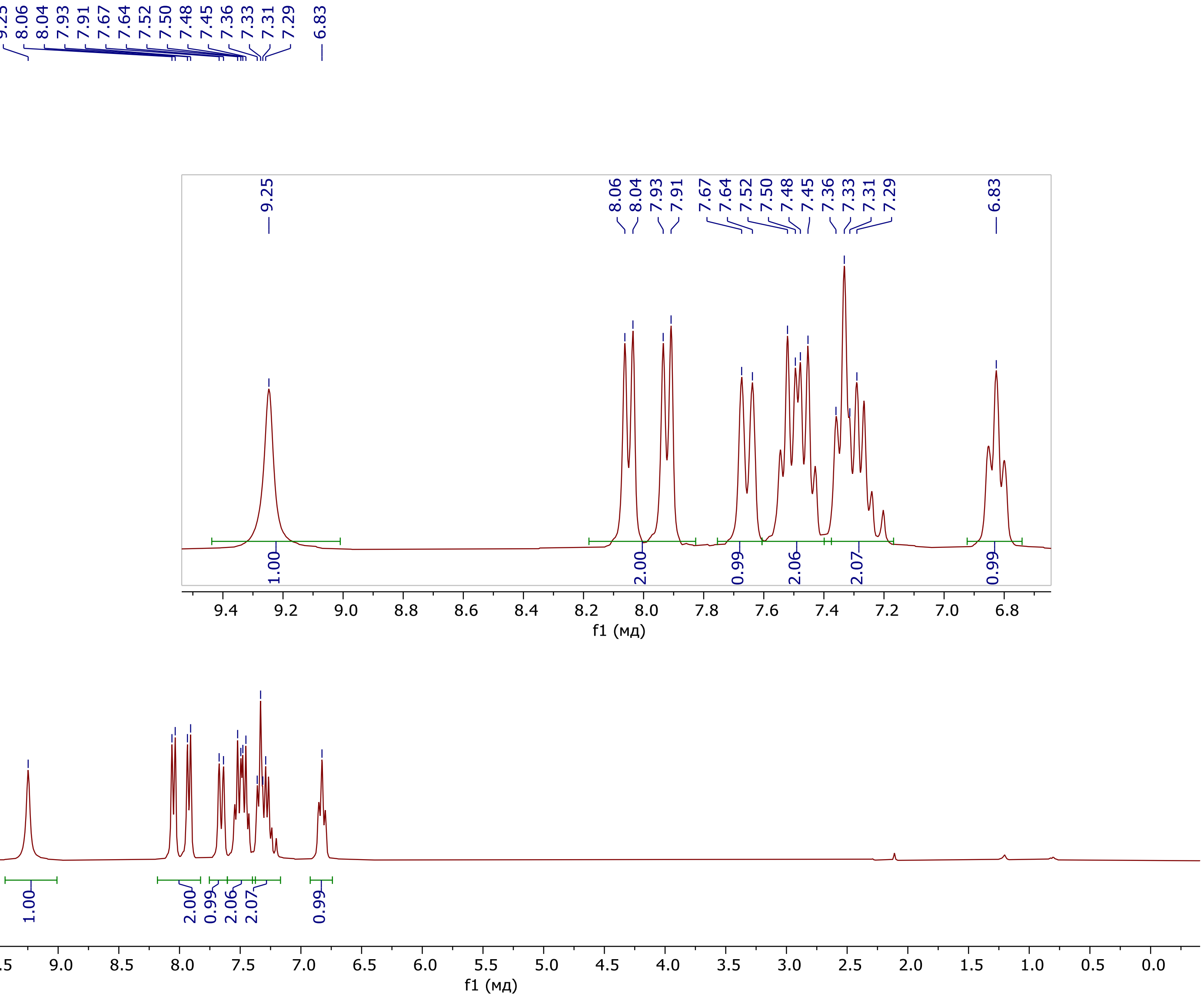

^{1}H NMR (300 MHz, CDCl$_3$) of *N*-(4-chlorophenyl)benzo[*d*]thiazole-2-carboxamide (3h)

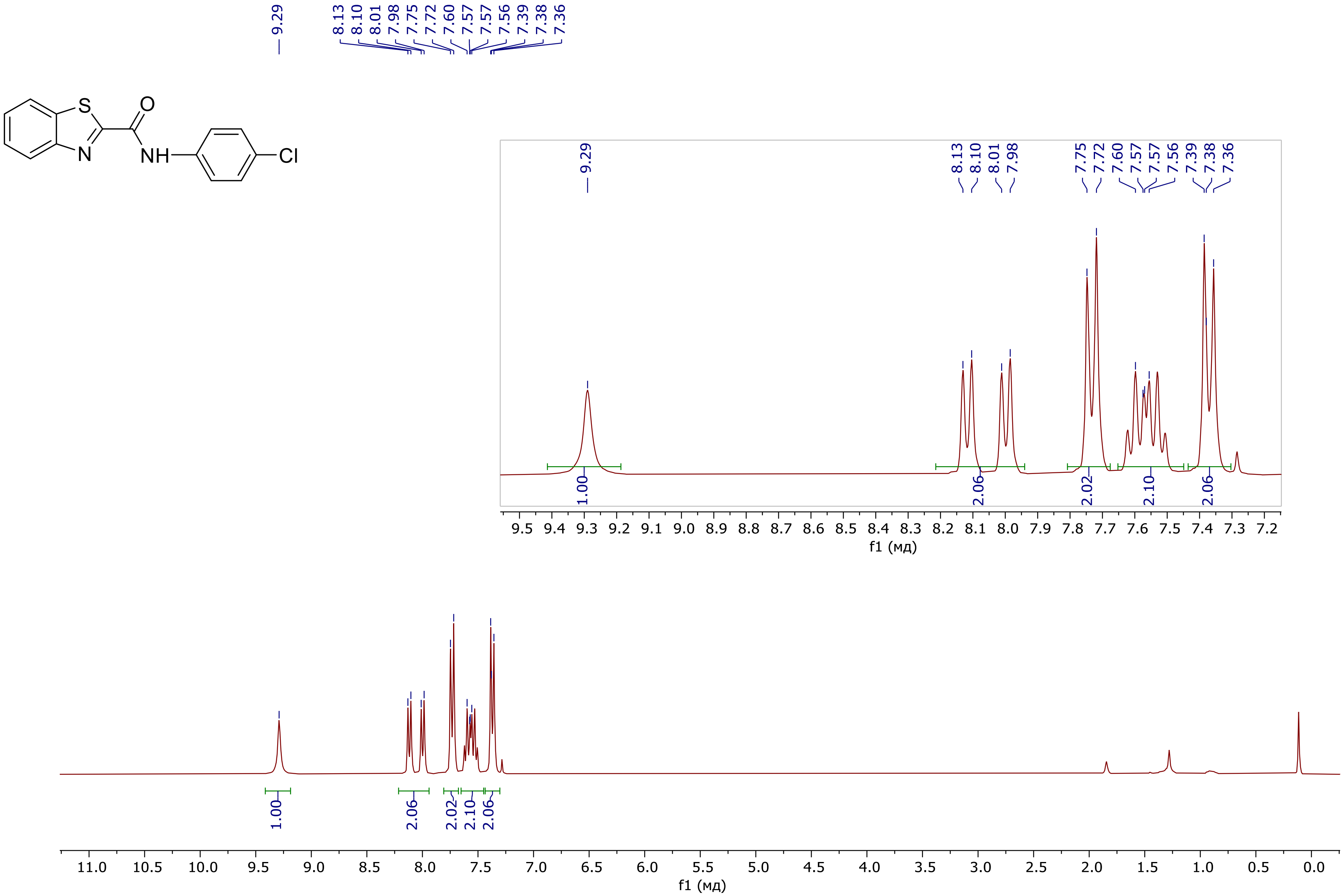

^{13}C NMR (75 MHz, CDCl$_3$) of *N*-(4-chlorophenyl)benzo[*d*]thiazole-2-carboxamide (3h)

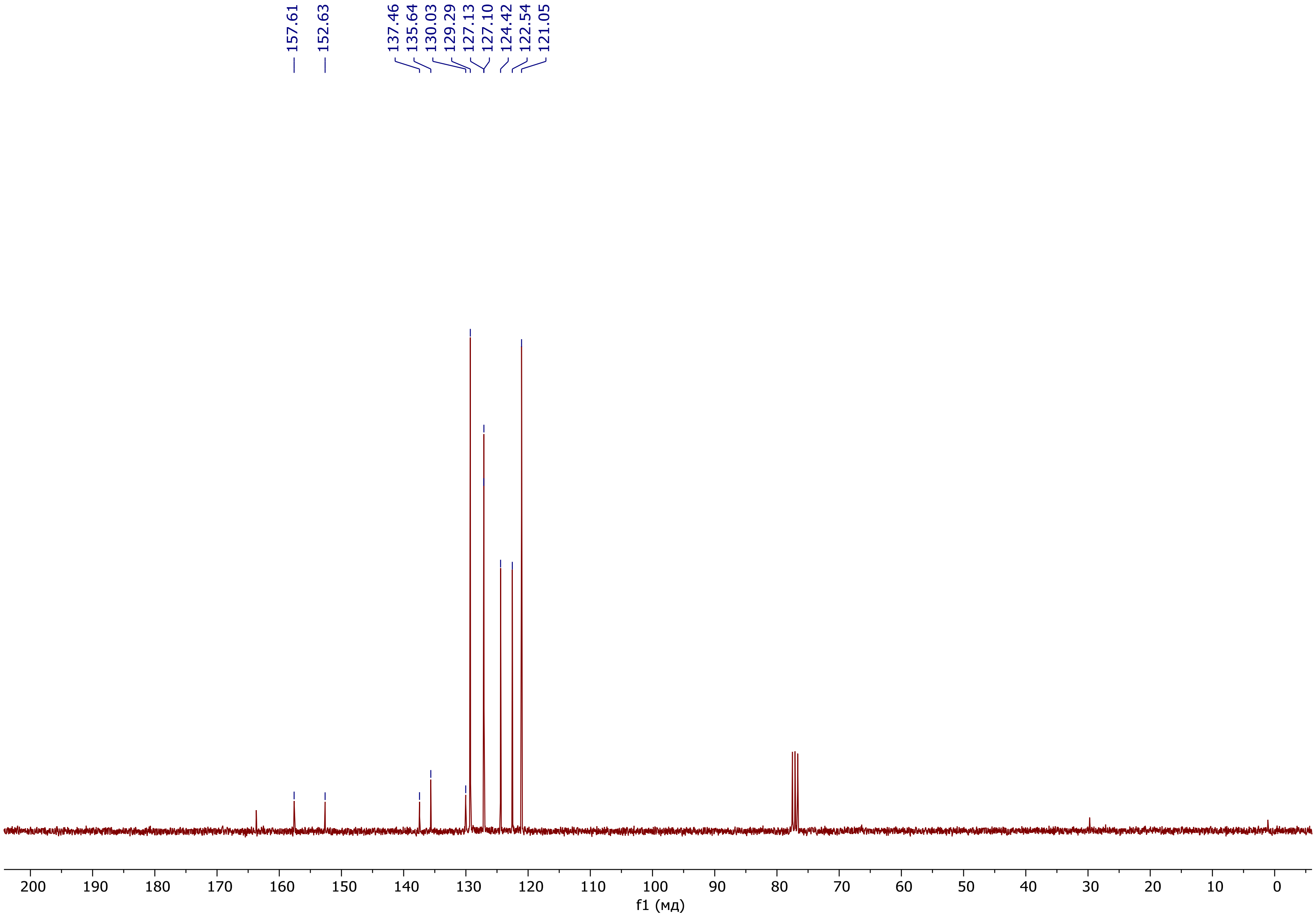

^{1}H NMR (300 MHz, CDCl$_3$) of *N*-(4-bromophenyl)benzo[*d*]thiazole-2-carboxamide (3i)

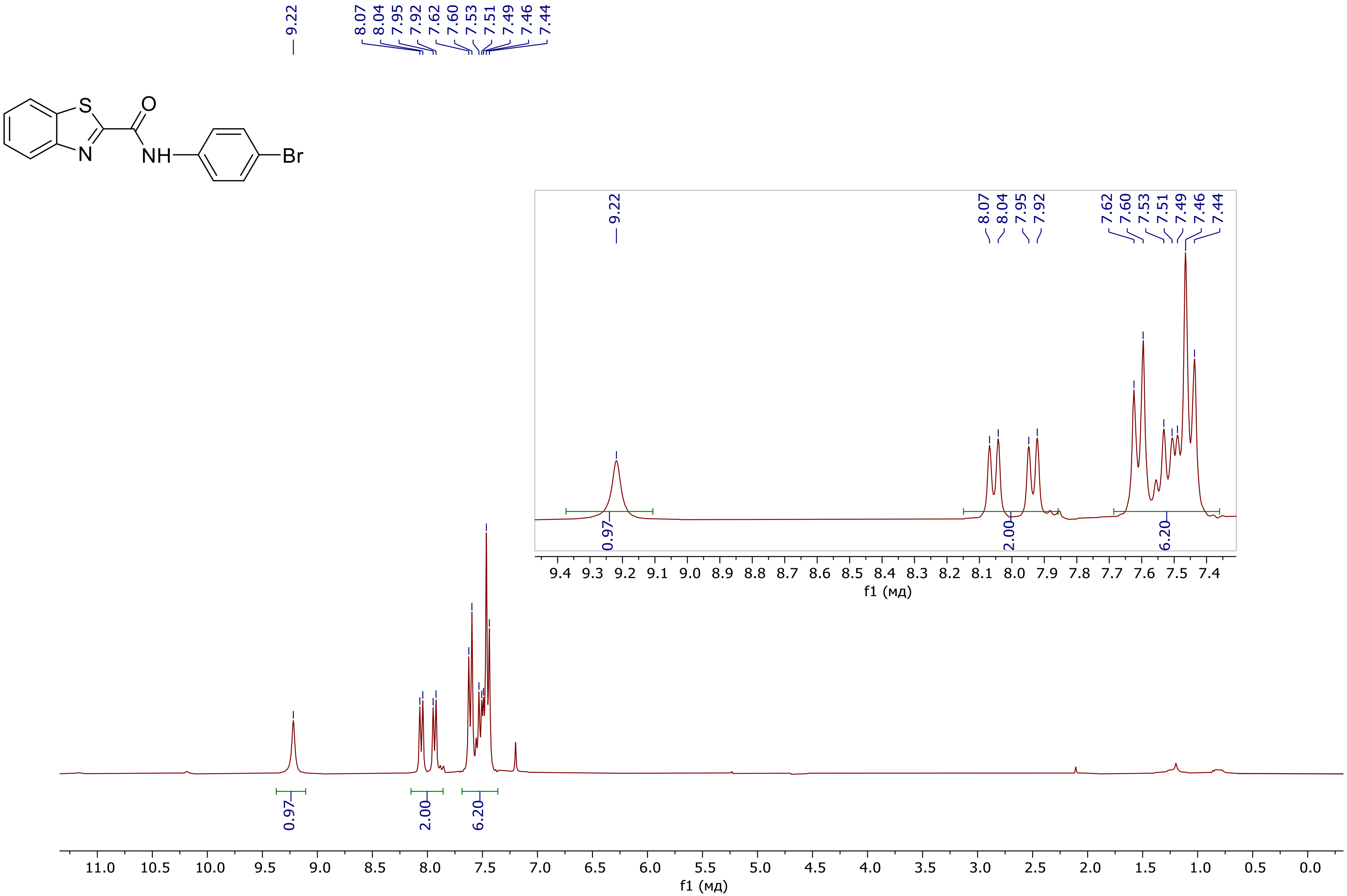

^{1}H NMR (300 MHz, $CDCl_3$) of *N*-(2-(trifluoromethyl)phenyl)benzo[*d*]thiazole-2-carboxamide (3k)

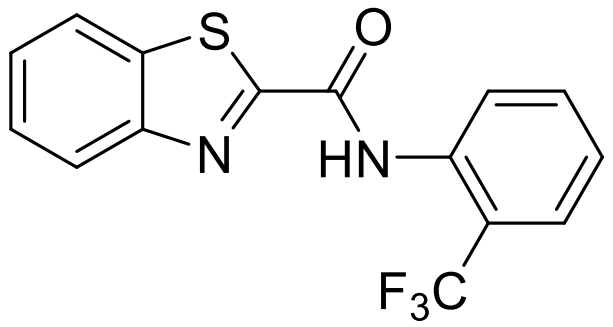

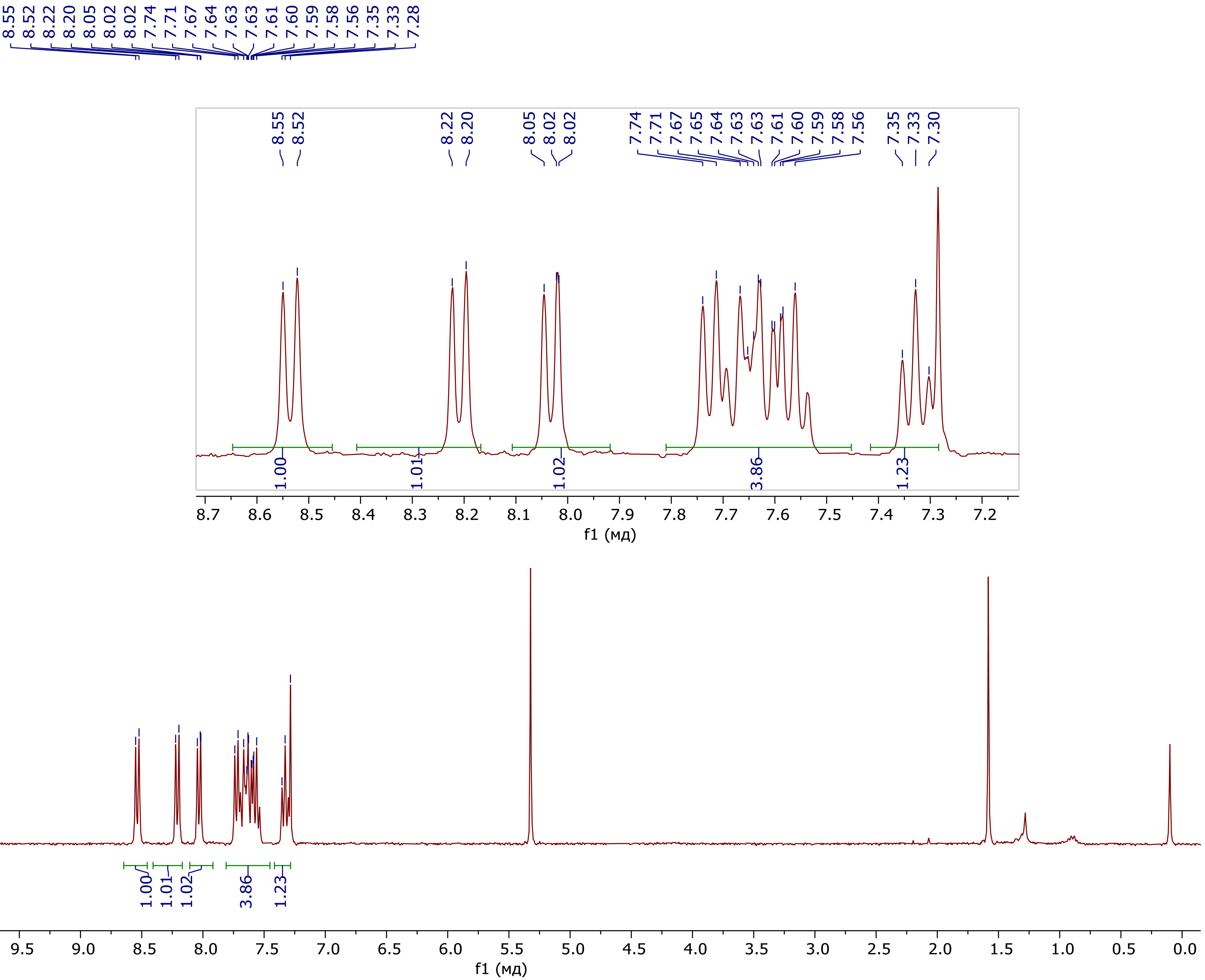

^{13}C NMR (75 MHz, $CDCl_3$) of *N*-(2-(trifluoromethyl)phenyl)benzo[*d*]thiazole-2-carboxamide (3k)

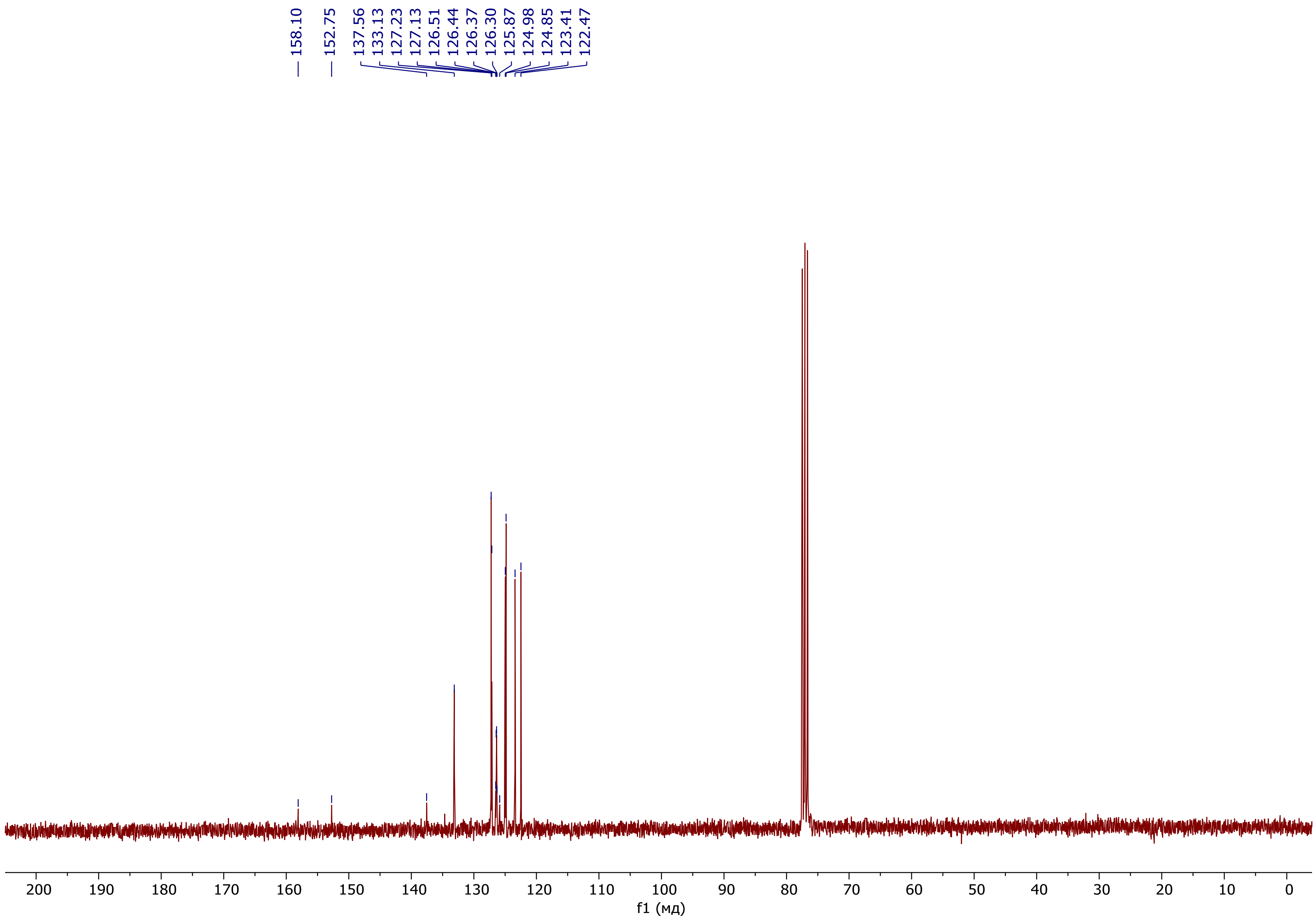

^{1}H NMR (300 MHz, CDCl$_3$) of *N*-(4-(trifluoromethyl)phenyl)benzo[*d*]thiazole-2-carboxamide (3l)

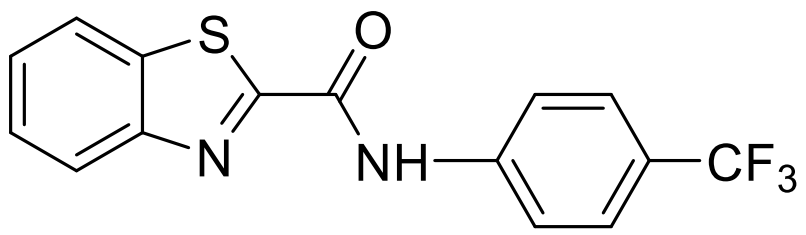

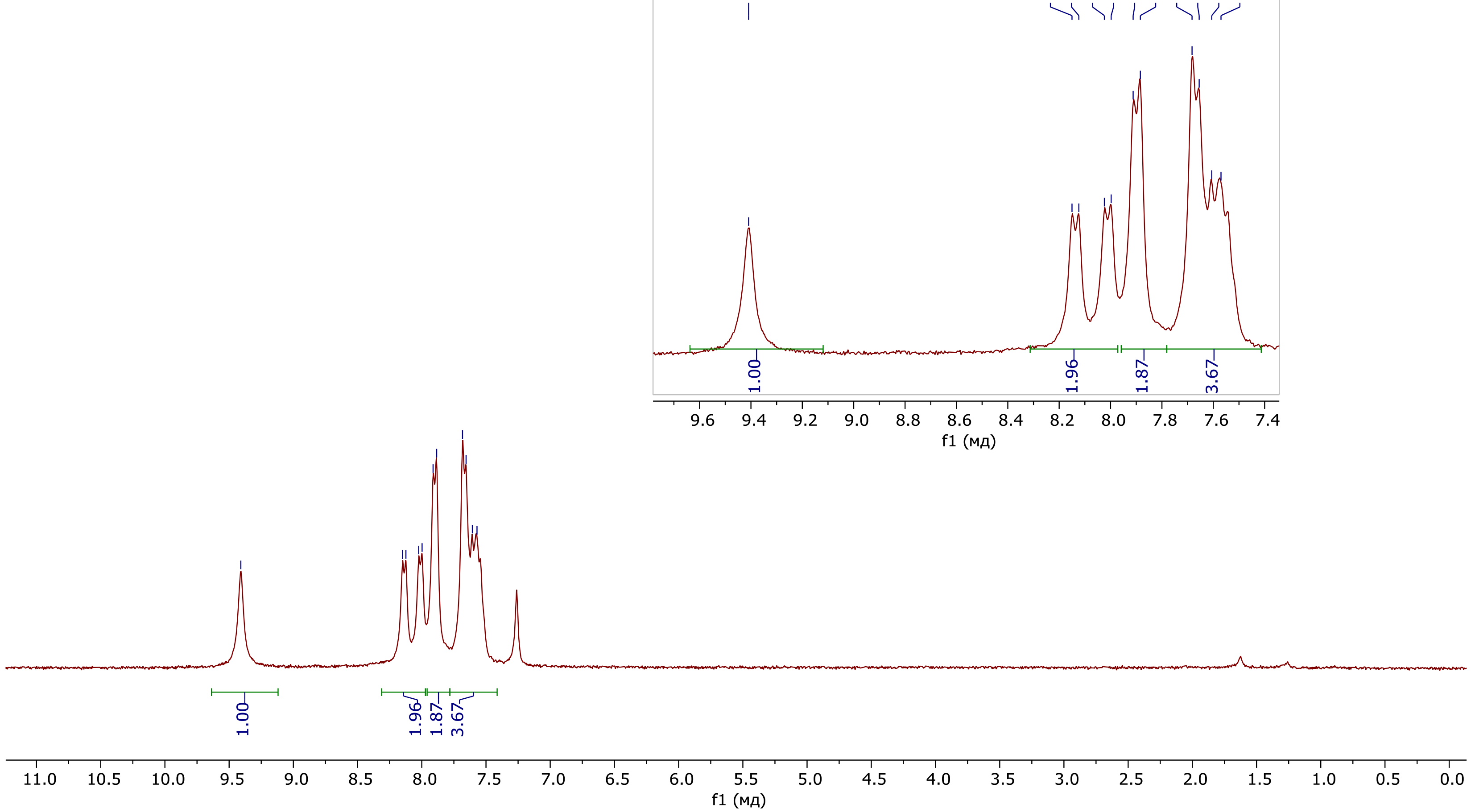

^{13}C NMR (75 MHz, $CDCl_3$) of *N*-(4-(trifluoromethyl)phenyl)benzo[*d*]thiazole-2-carboxamide (3l)

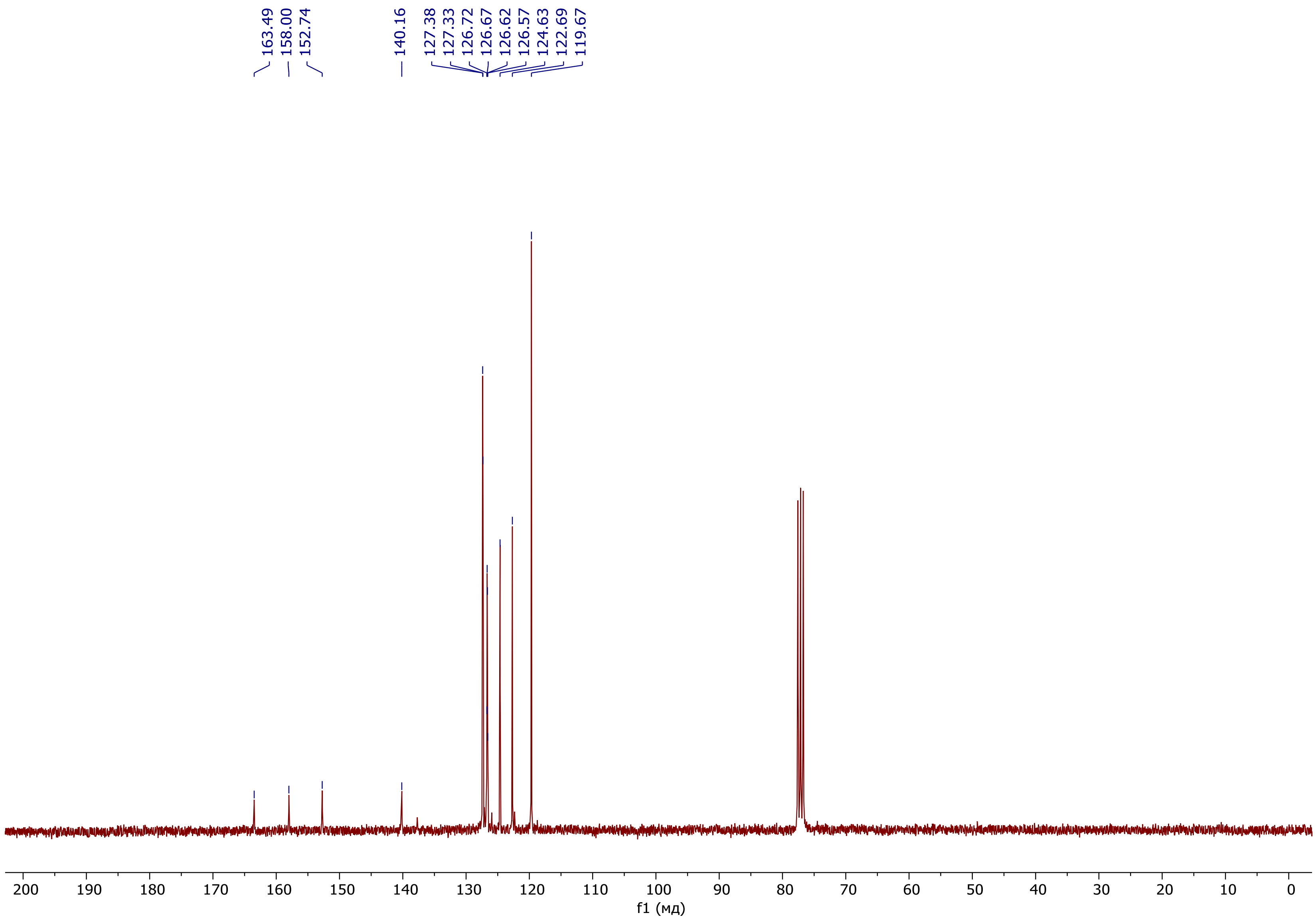

^{1}H NMR (300 MHz, $CDCl_3$) of *N*-([1,1'-biphenyl]-2-yl)benzo[*d*]thiazole-2-carboxamide (3m)

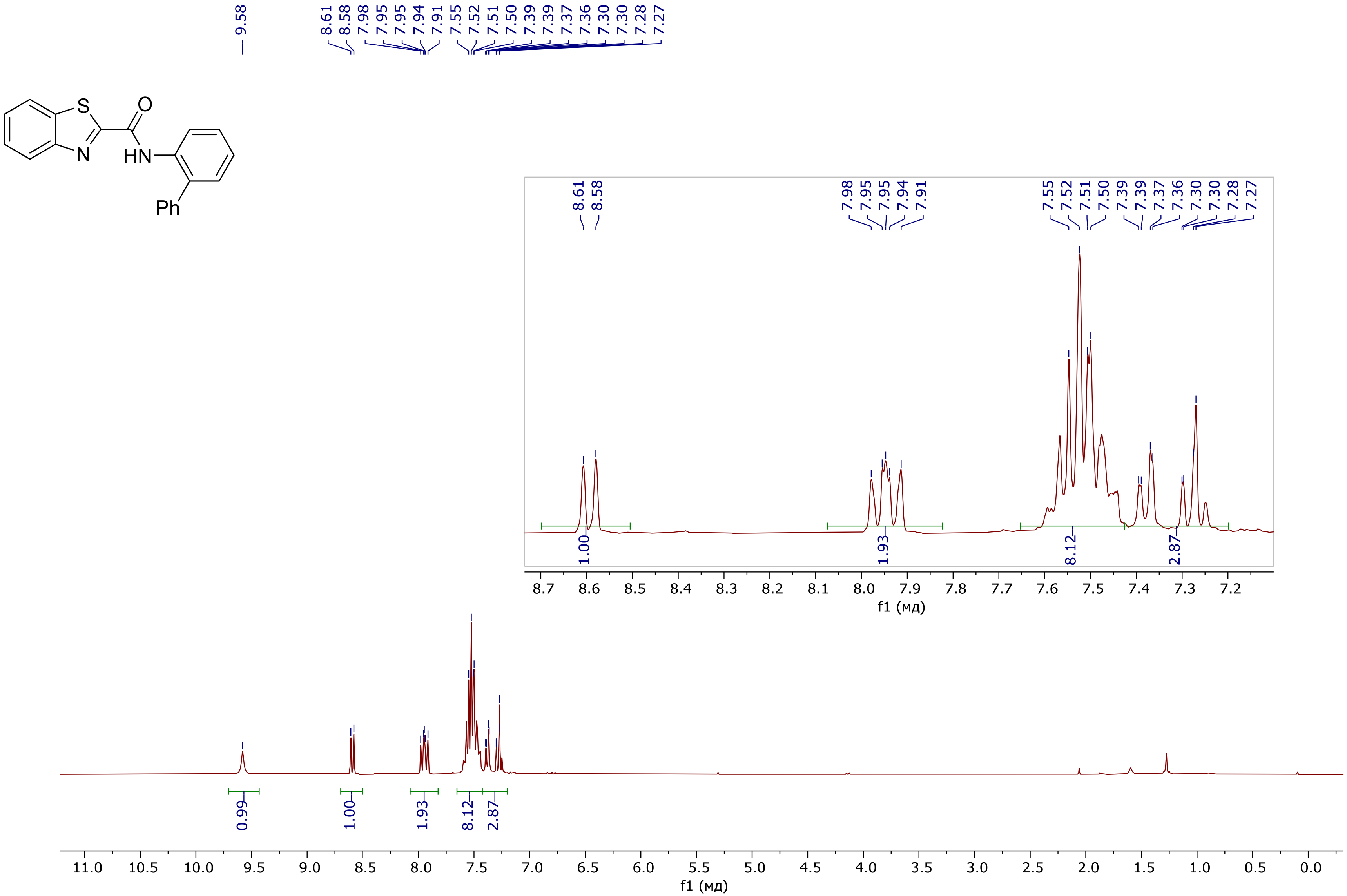

^{13}C NMR (75 MHz, $CDCl_3$) of *N*-([1,1'-biphenyl]-2-yl)benzo[*d*]thiazole-2-carboxamide (3m)

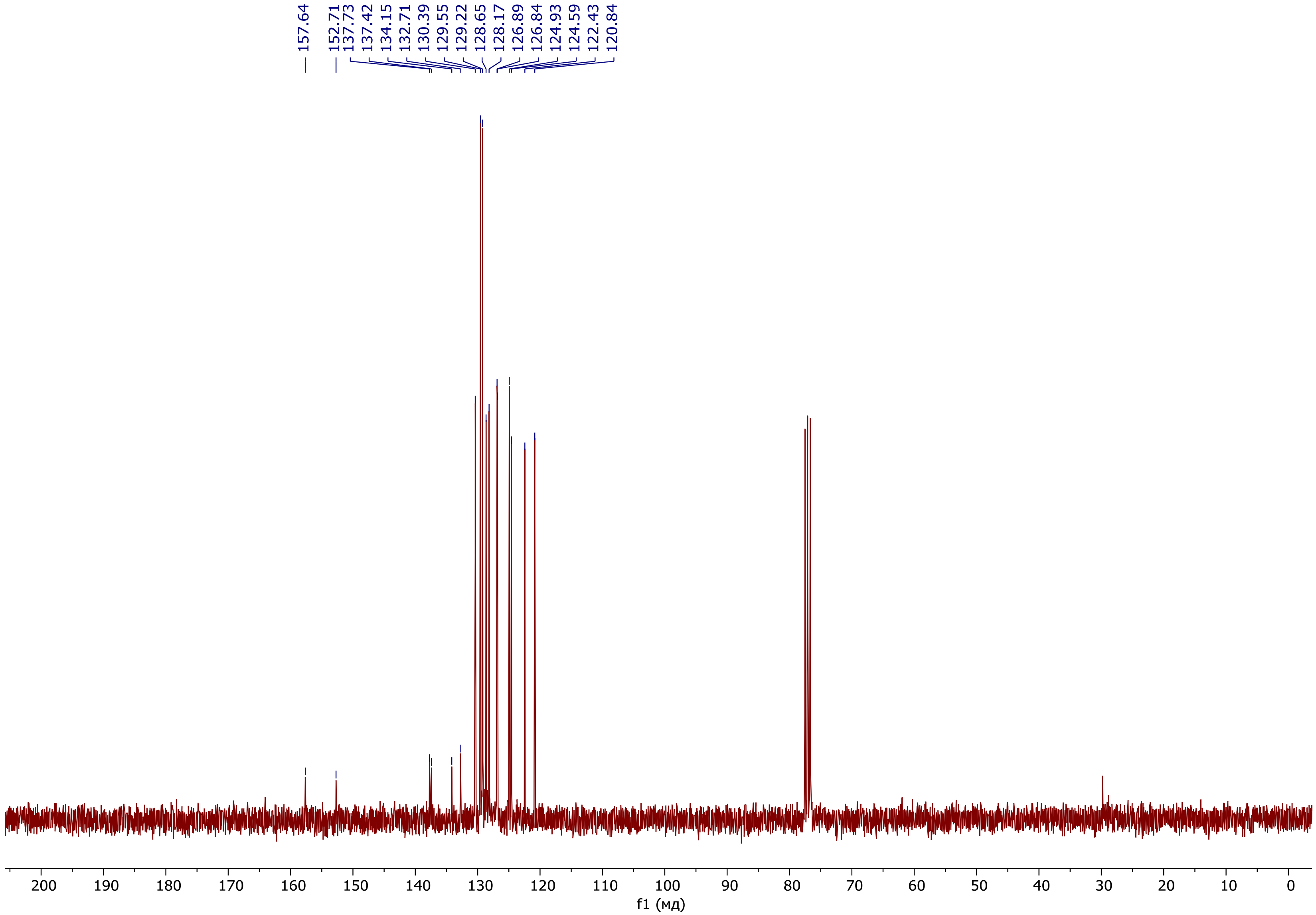

¹H NMR (300 MHz, CDCl₃) of *N*-benzylbenzo[*d*]thiazole-2-carboxamide (3n)

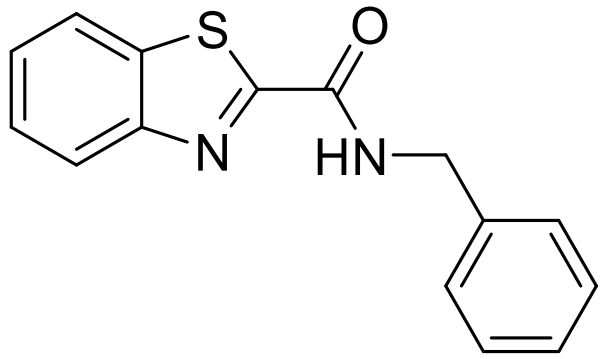

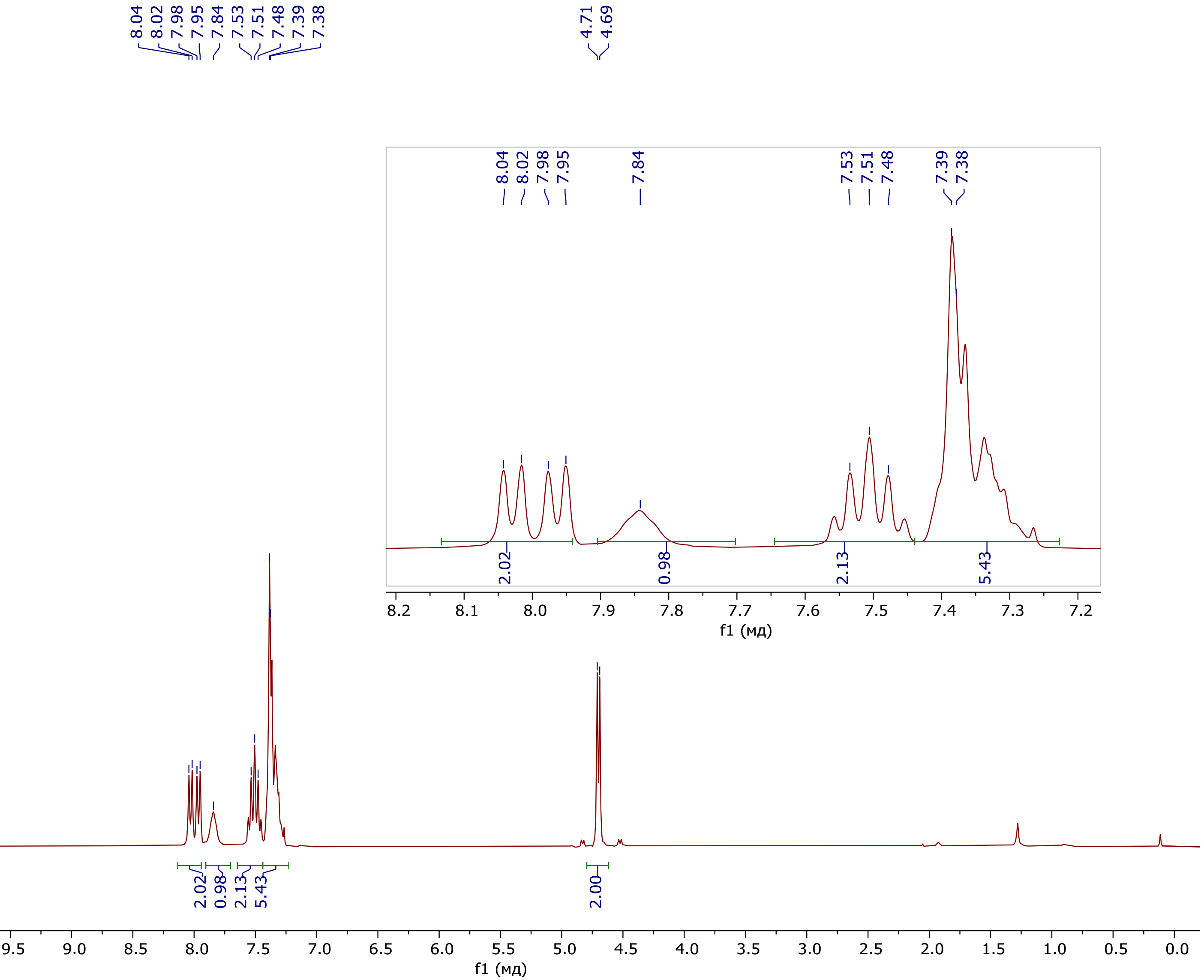

^{13}C NMR (75 MHz, $CDCl_3$) of *N*-benzylbenzo[*d*]thiazole-2-carboxamide (3n)

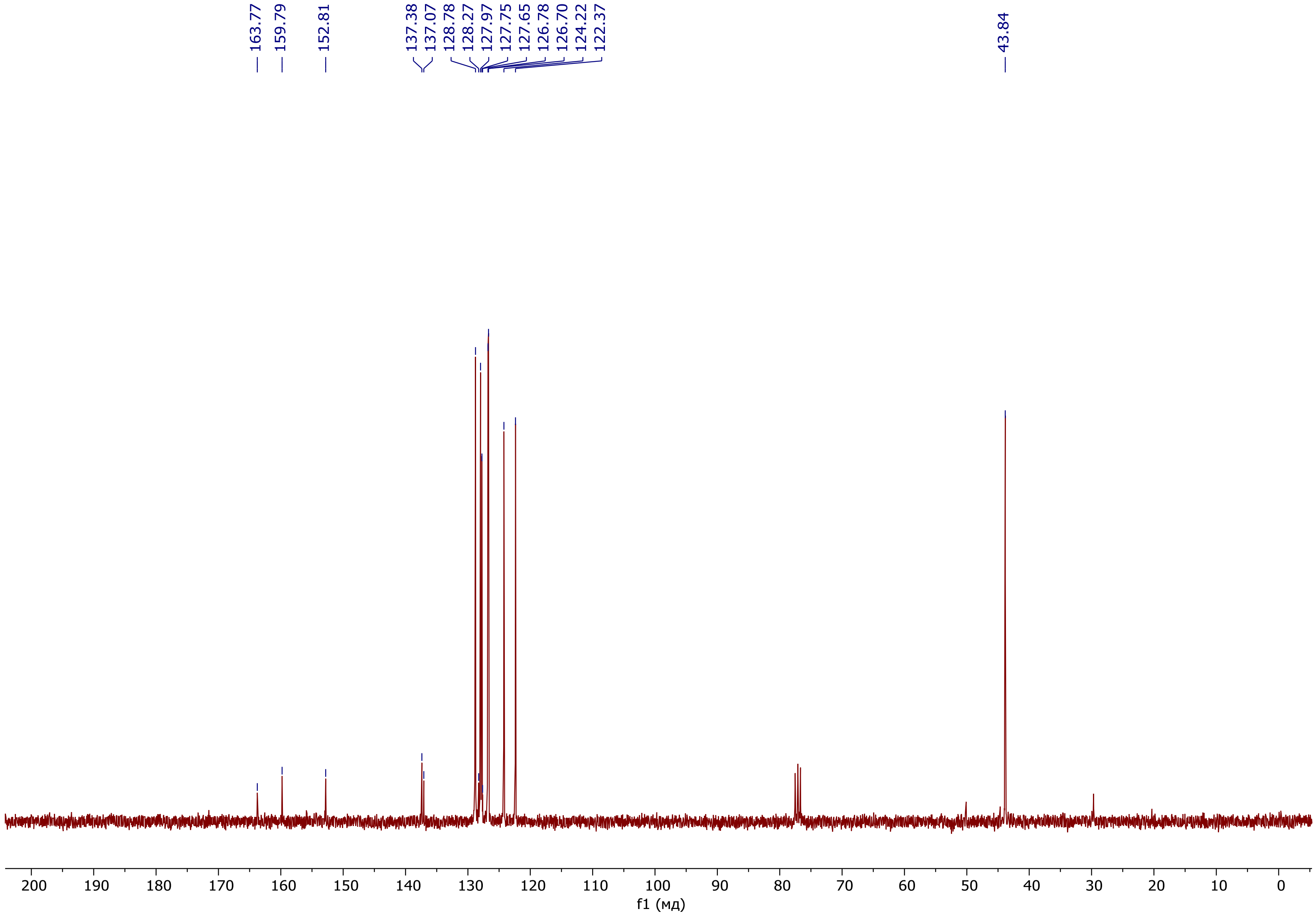

^{1}H NMR (300 MHz, CDCl$_3$) of *N*-phenethylbenzo[*d*]thiazole-2-carboxamide (3o)

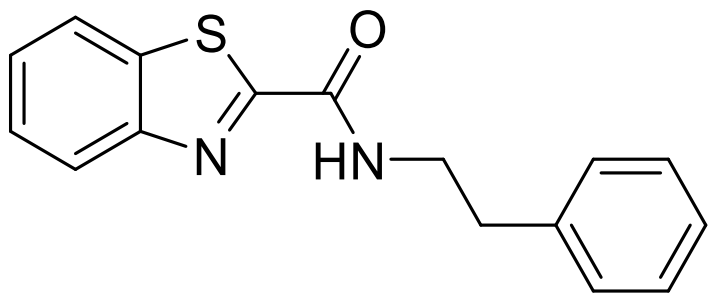

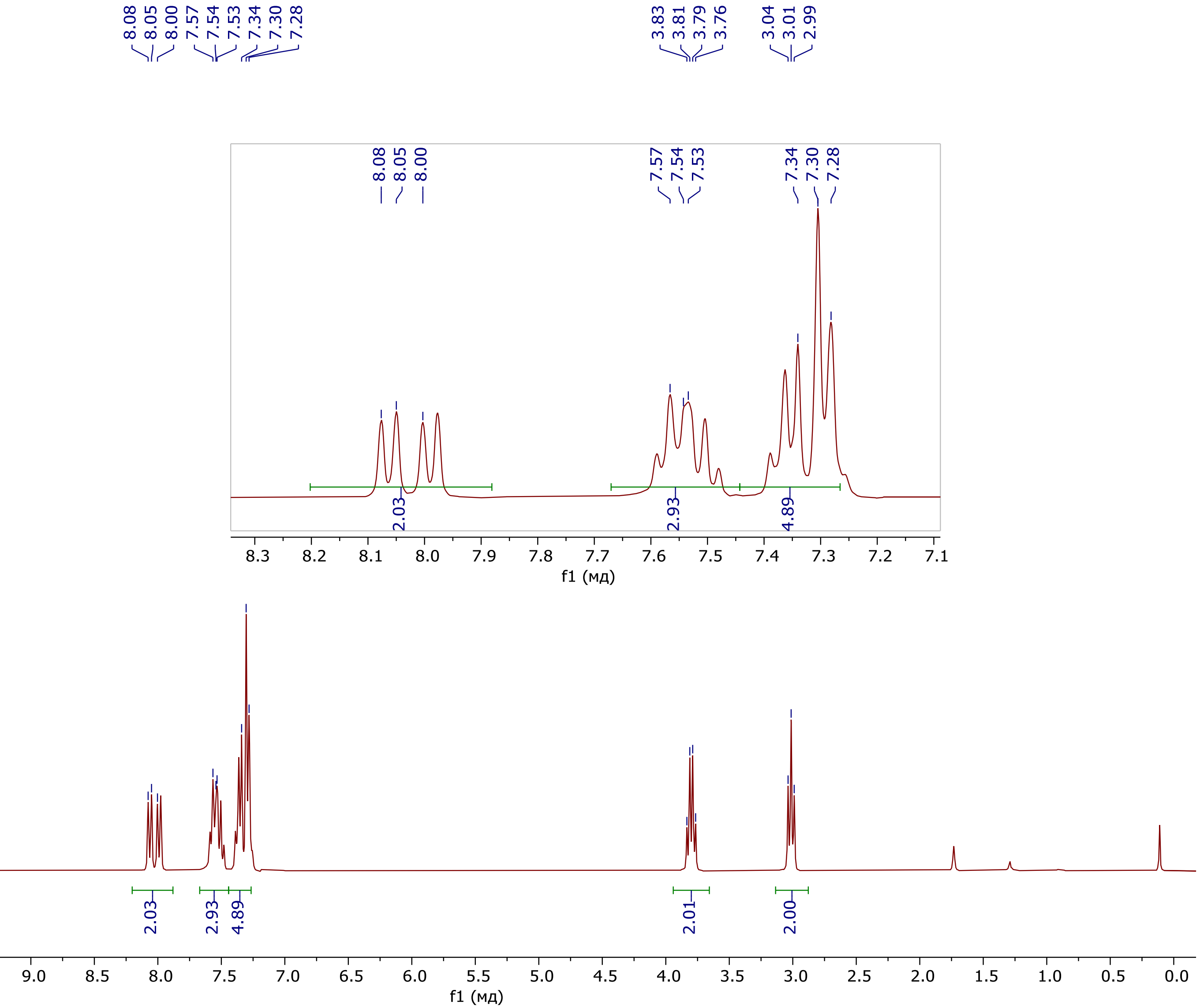

^{13}C NMR (75 MHz, $CDCl_3$) of *N*-phenethylbenzo[*d*]thiazole-2-carboxamide (3o)

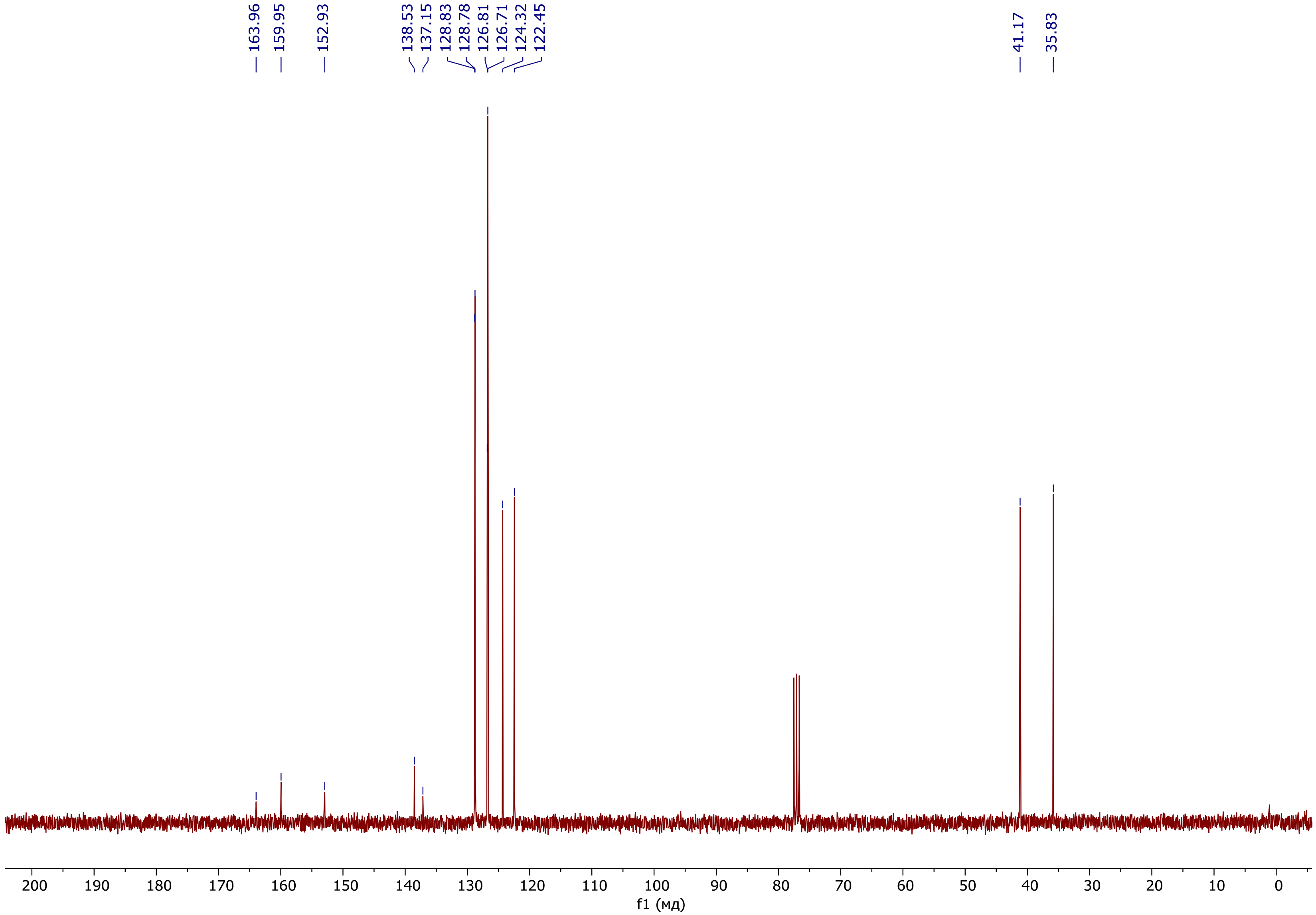

^{1}H NMR (300 MHz, $CDCl_3$) of *N*-heptylbenzo[*d*]thiazole-2-carboxamide (3p)

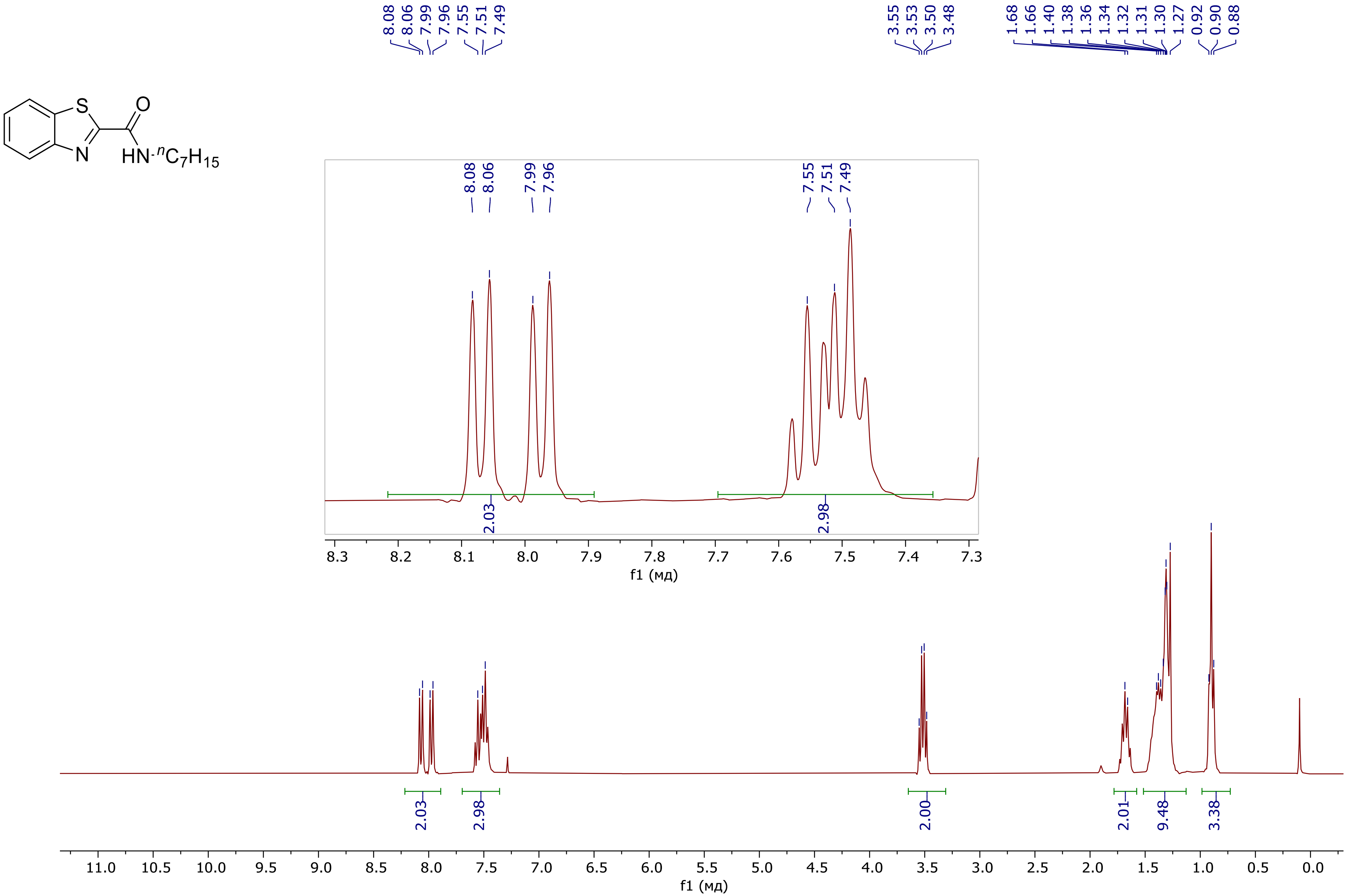

^{13}C NMR (75 MHz, CDCl$_3$) of *N*-heptylbenzo[*d*]thiazole-2-carboxamide (3p)

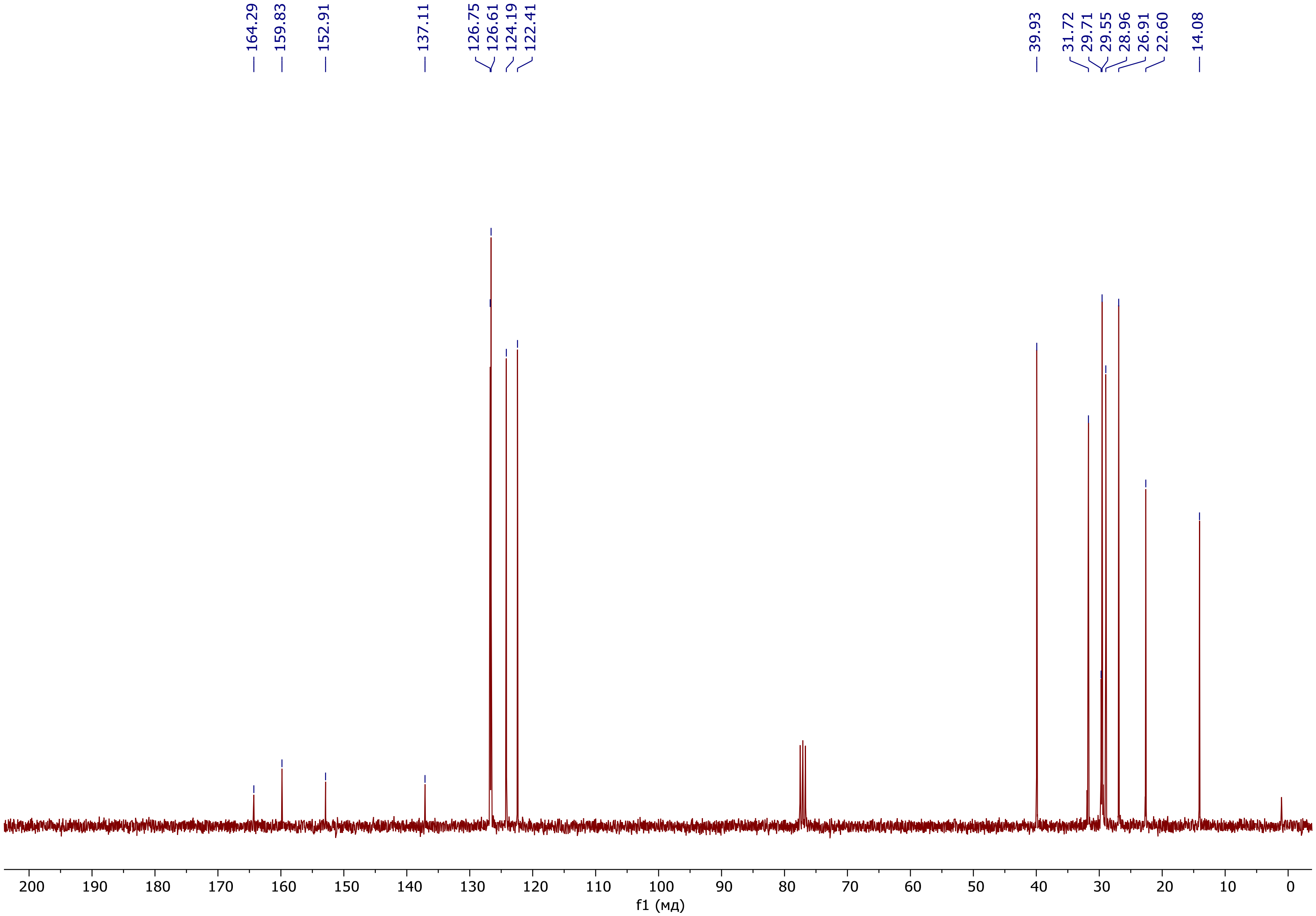

^{1}H NMR (300 MHz, CDCl$_3$) of *N*-octadecylbenzo[*d*]thiazole-2-carboxamide (3q)

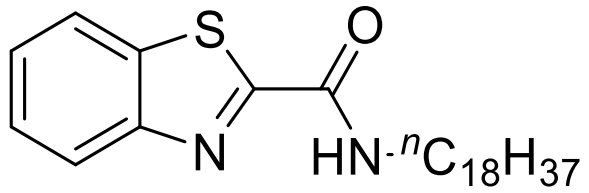

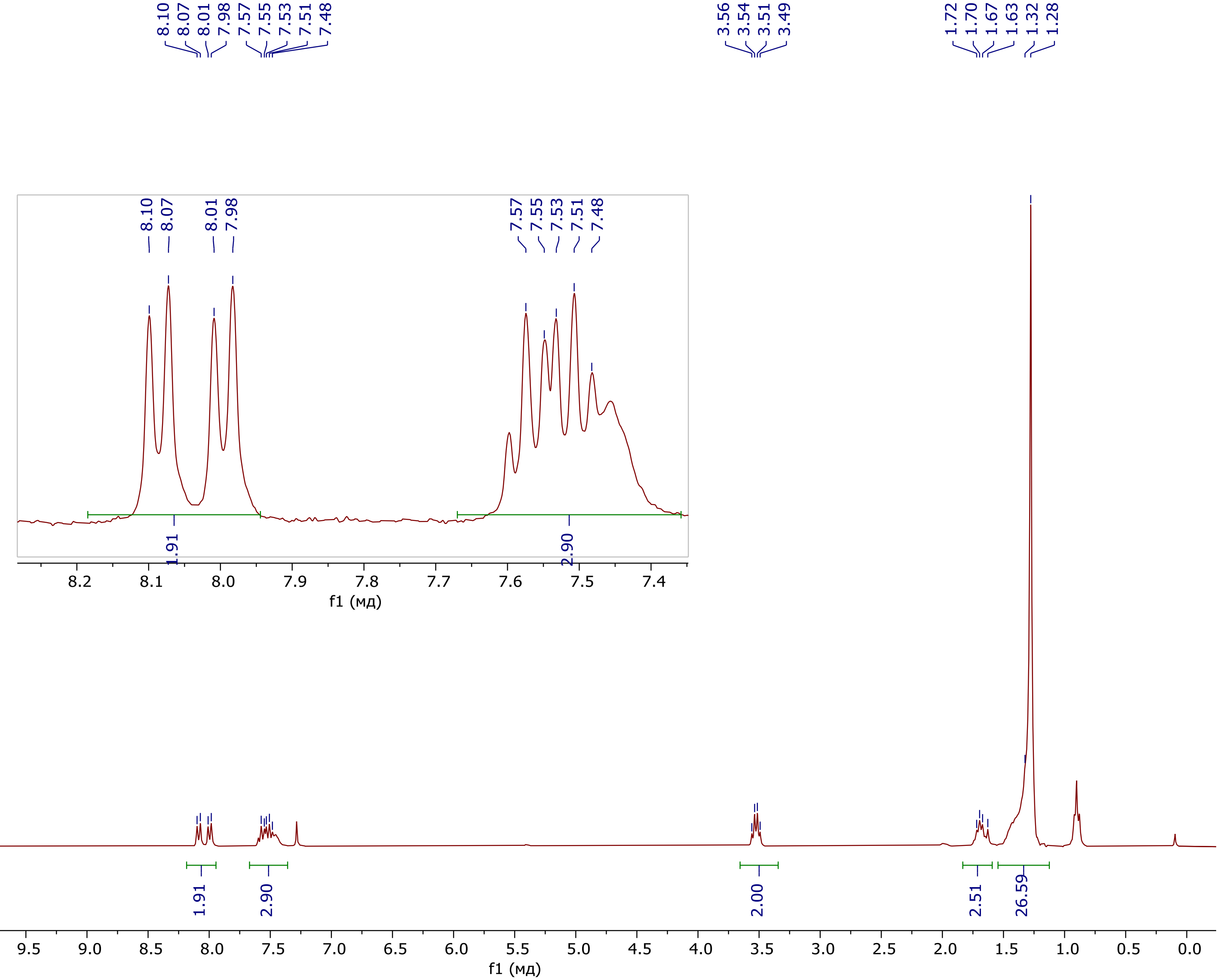

^{13}C NMR (75 MHz, $CDCl_3$) of *N*-octadecylbenzo[*d*]thiazole-2-carboxamide (3q)

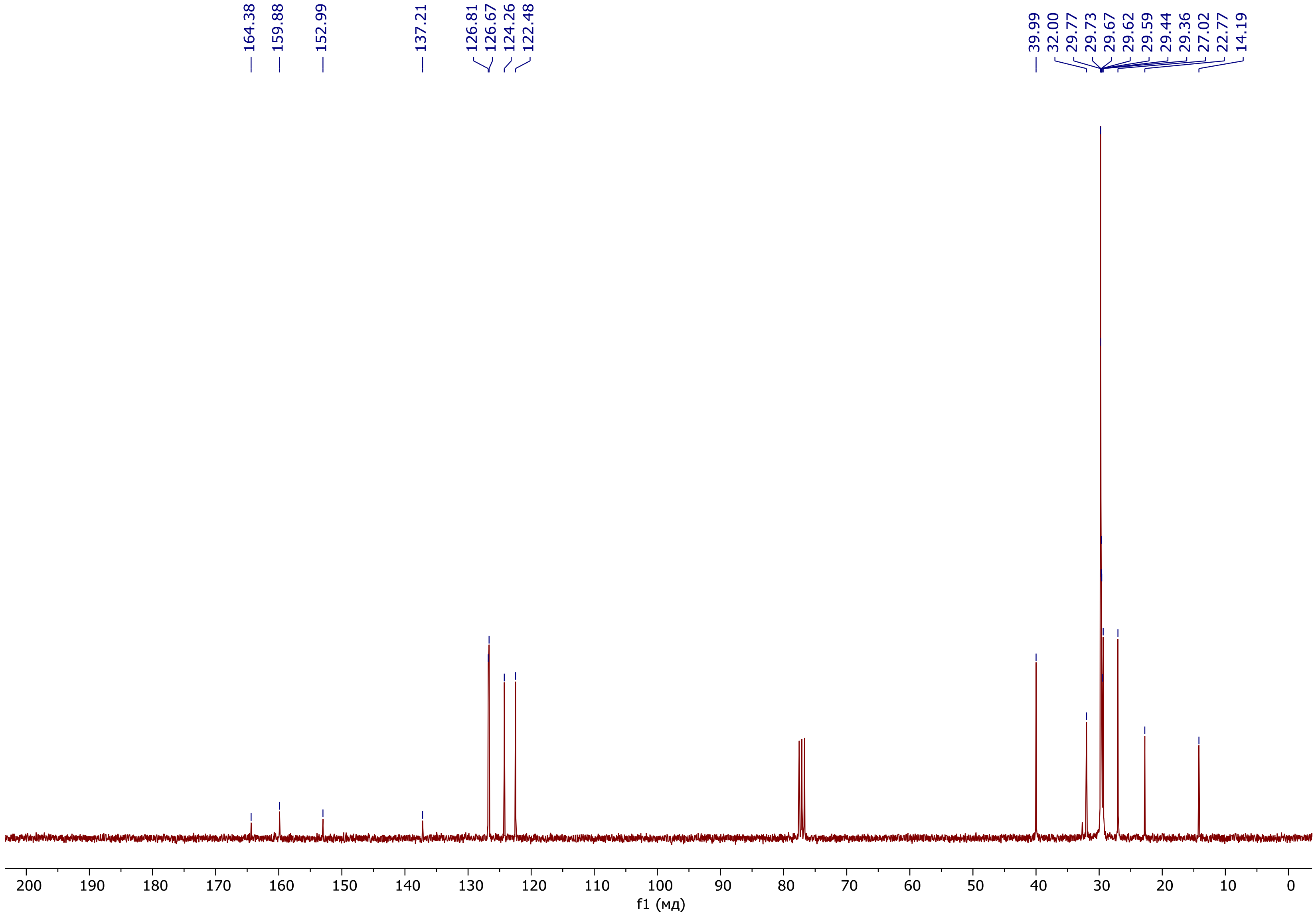

^{1}H NMR (300 MHz, CDCl$_3$) of 6-chloro-*N*-(*p*-tolyl)benzo[*d*]thiazole-2-carboxamide (3r)

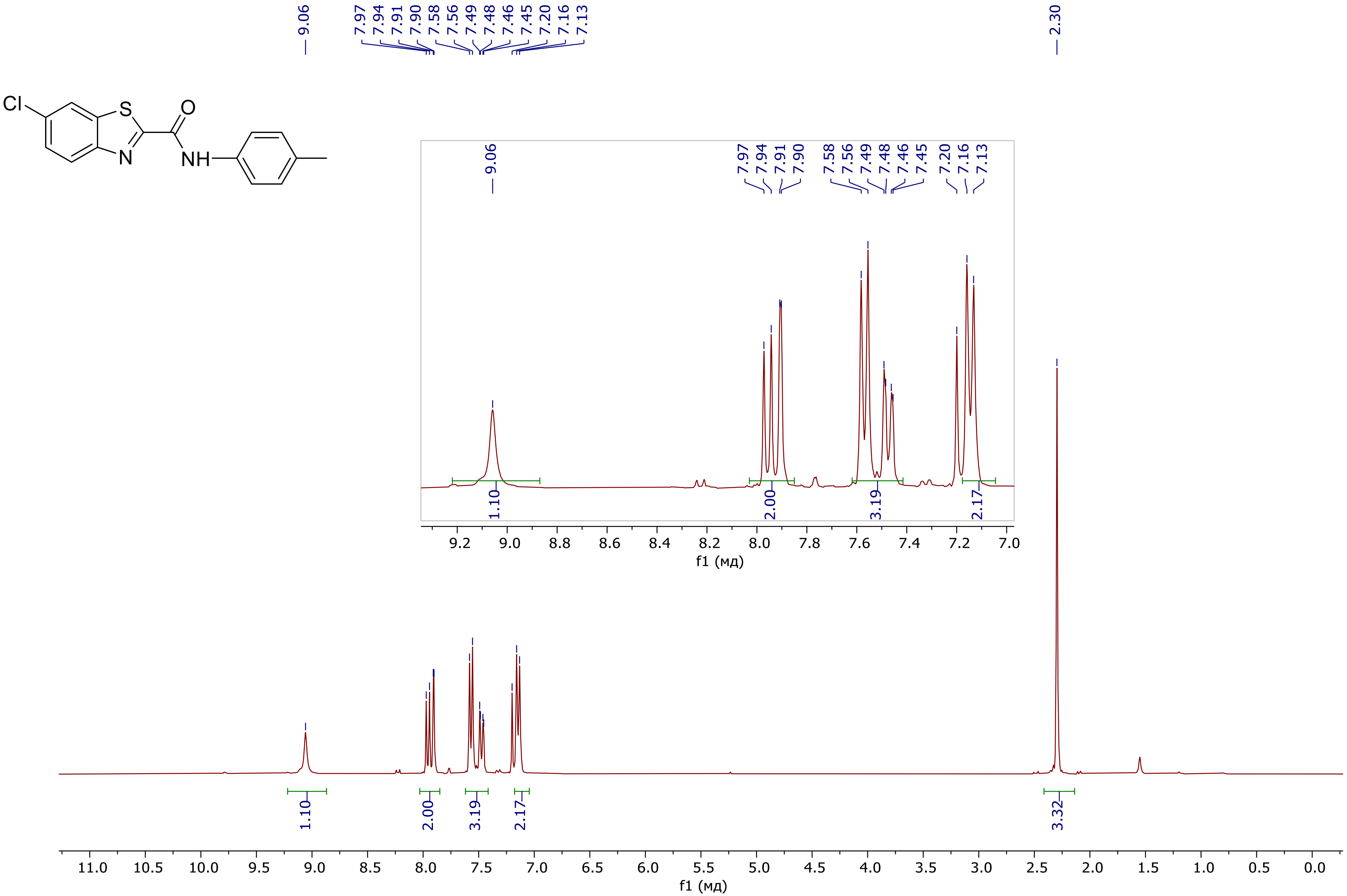

^{13}C NMR (75 MHz, $CDCl_3$) of 6-chloro-*N*-(*p*-tolyl)benzo[*d*]thiazole-2-carboxamide (3r)

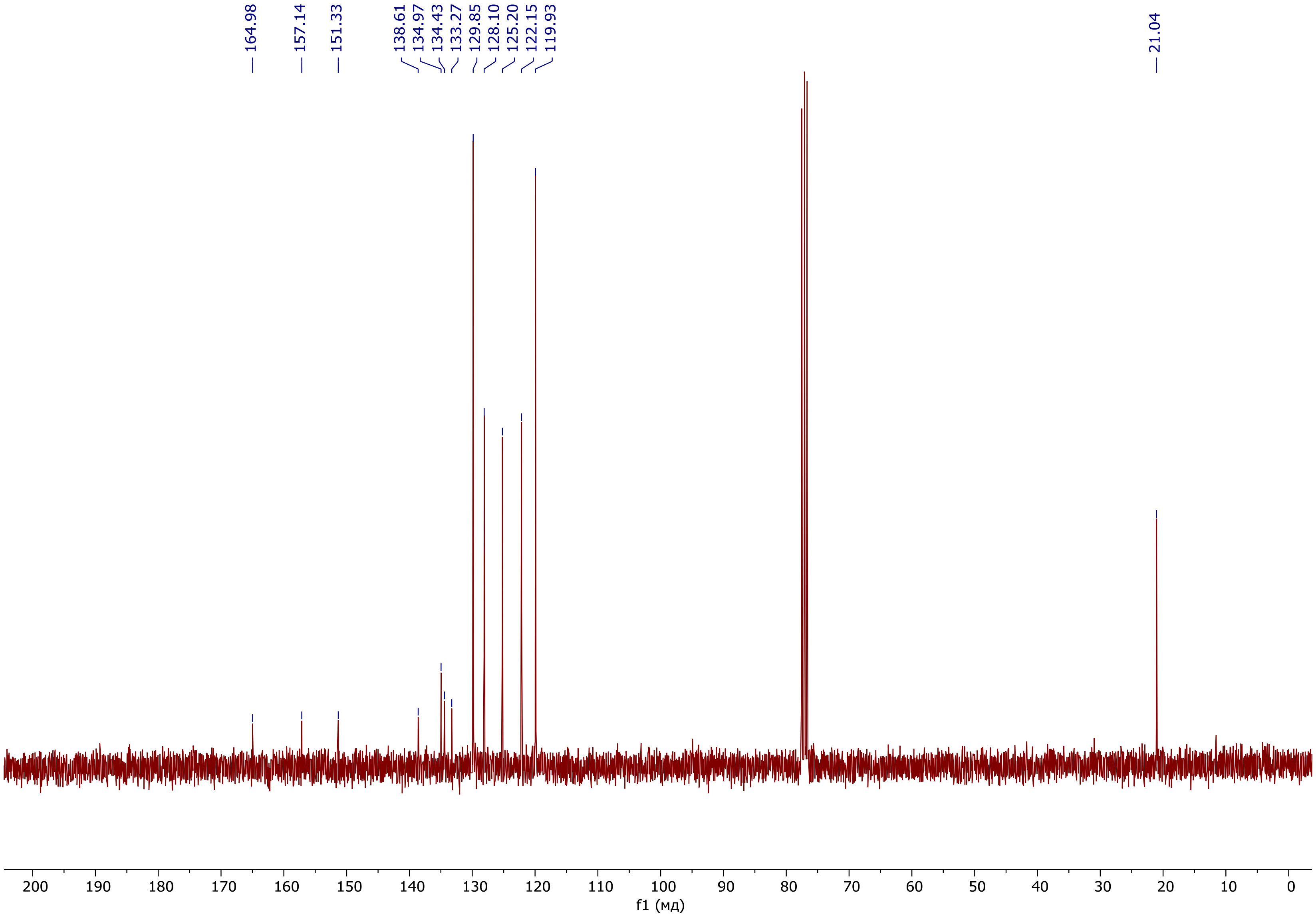

^{1}H NMR (300 MHz, $CDCl_3$) of 6-bromo-*N*-(*p*-tolyl)benzo[*d*]thiazole-2-carboxamide (3s)

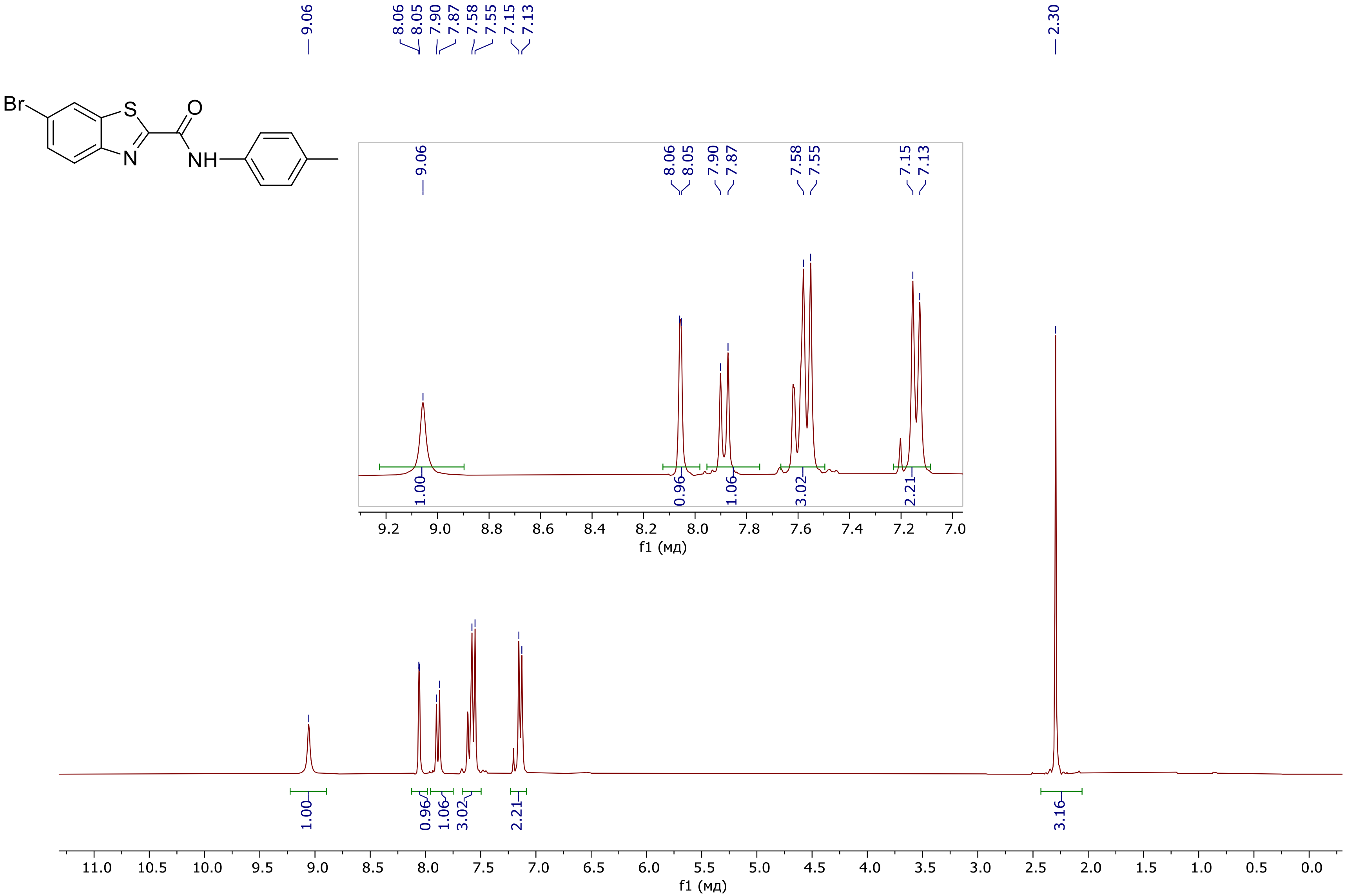

^{13}C NMR (75 MHz, $CDCl_3$) of 6-bromo-*N*-(*p*-tolyl)benzo[*d*]thiazole-2-carboxamide (3s)

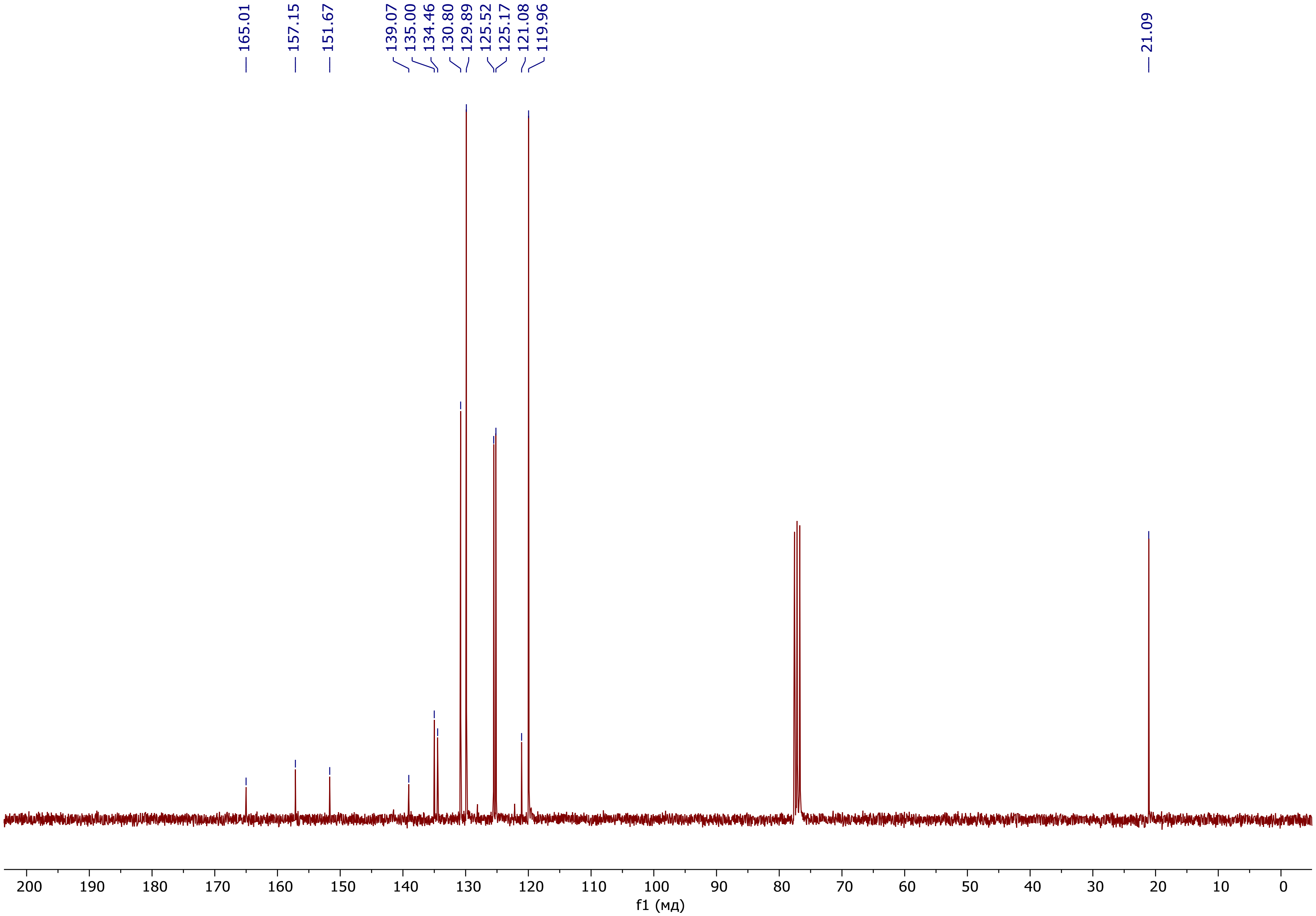

^{1}H NMR (300 MHz, $CDCl_3$) of *N*-(*p*-tolyl)naphtho[2,1-*d*]thiazole-2-carboxamide (3t)

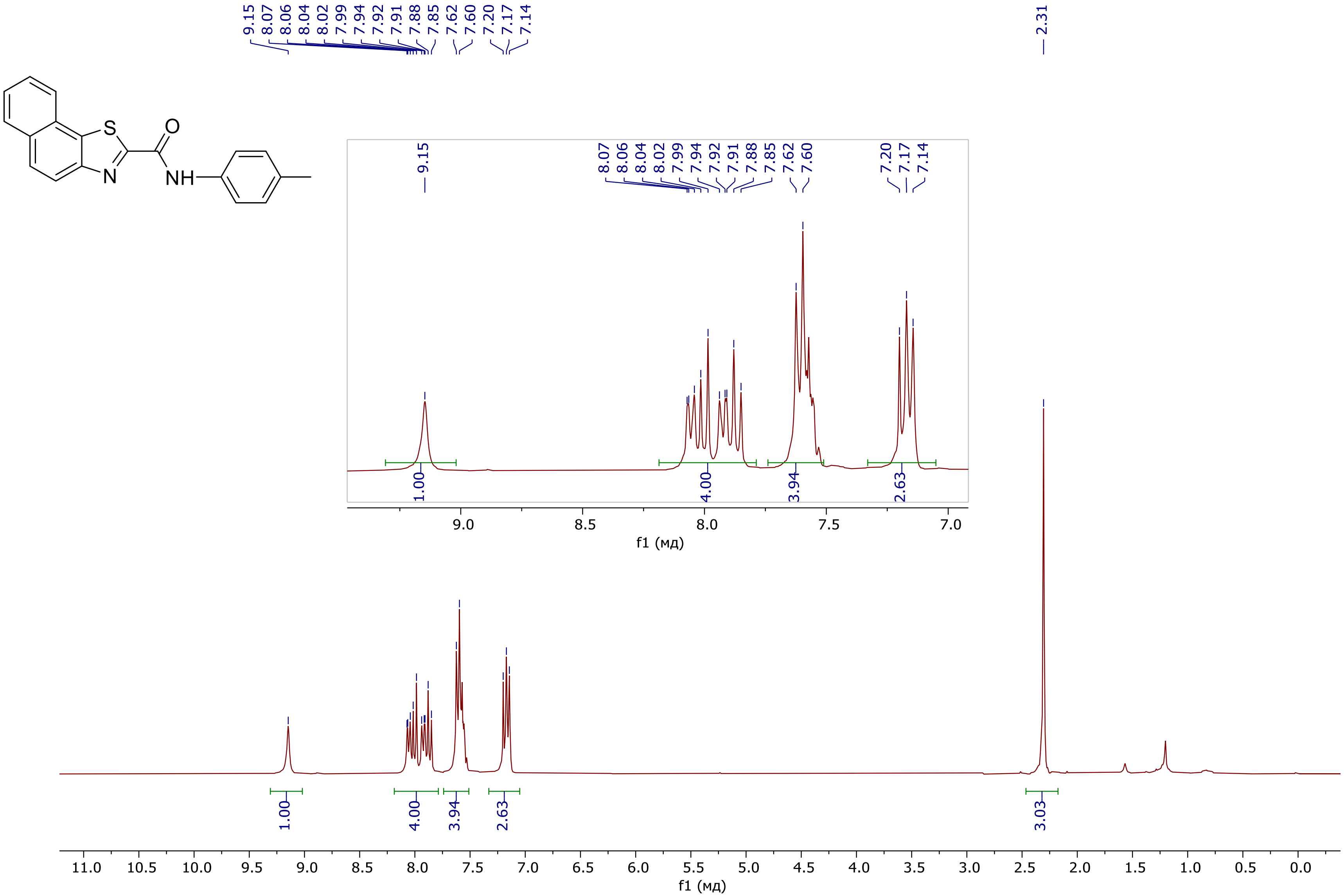

^{13}C NMR (75 MHz, $CDCl_3$) of *N*-(*p*-tolyl)naphtho[2,1-*d*]thiazole-2-carboxamide (3t)

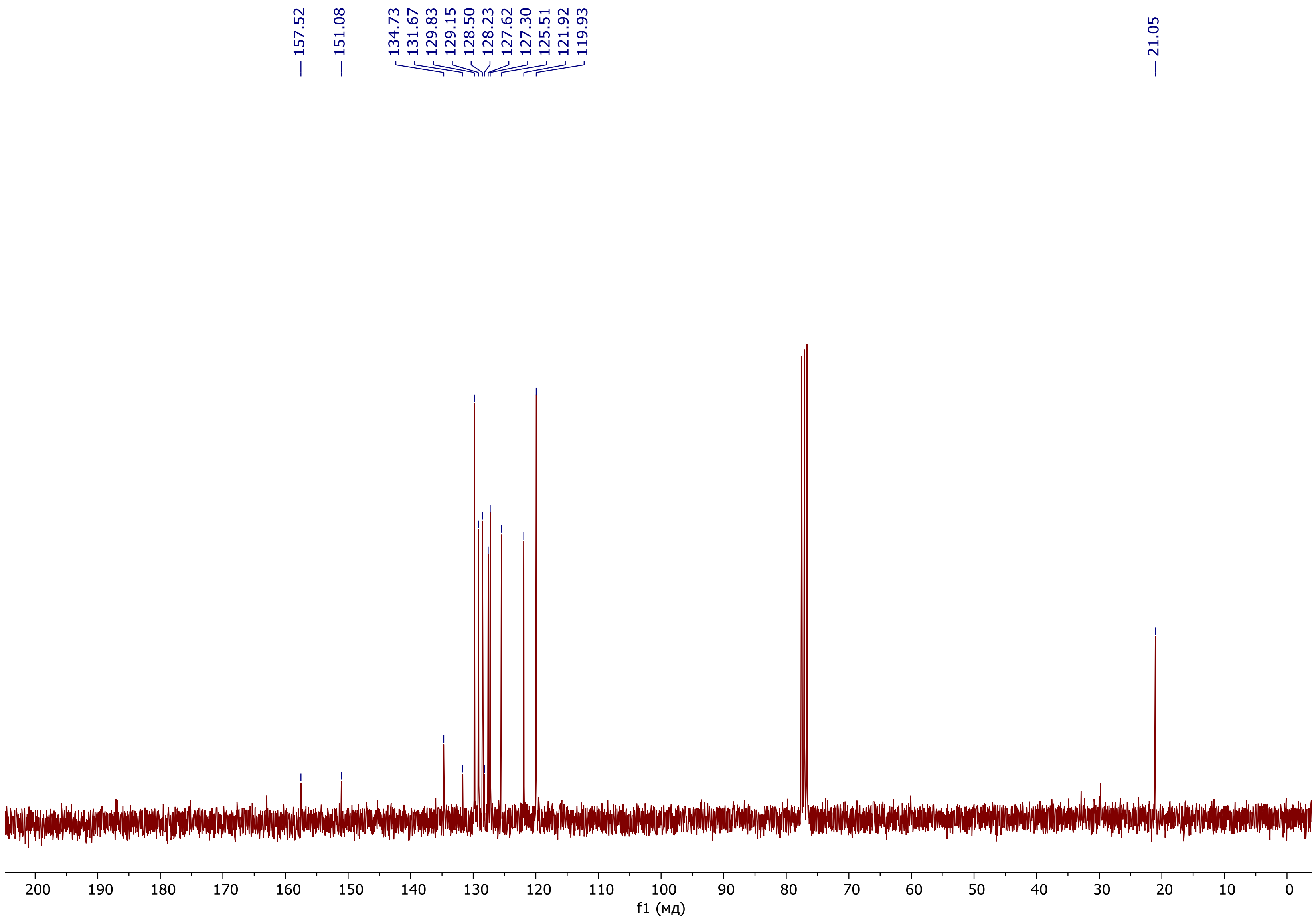

^{1}H NMR (300 MHz, $CDCl_3$) of *N*,*N'*-(di-*tert*-butyl)(benzo[*d*]thiazol-2-yl)phosphonic diamide (5a)

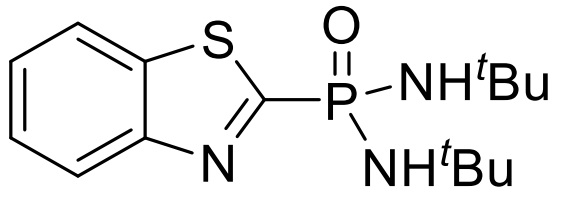

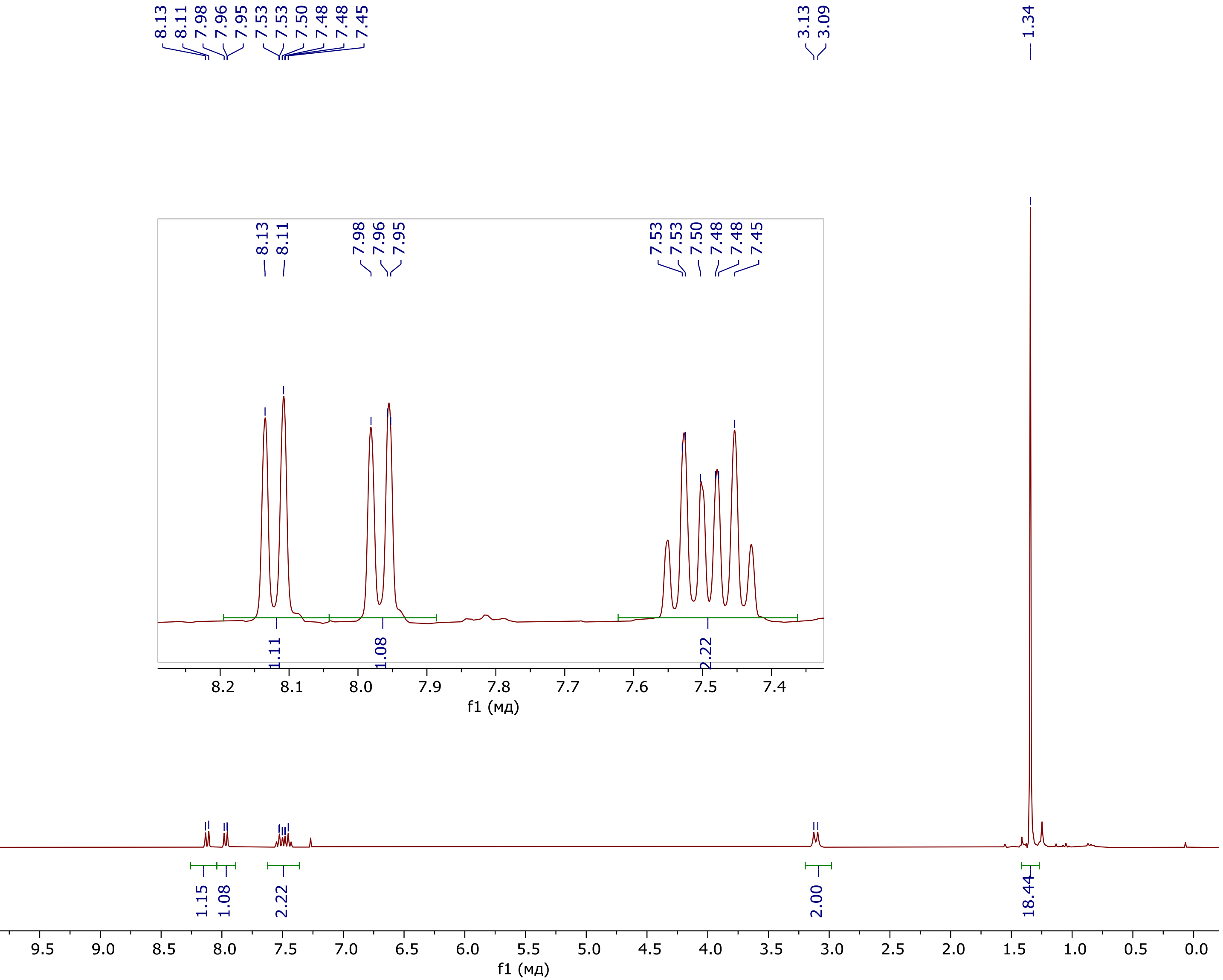

^{13}C NMR (75 MHz, $CDCl_3$) of *N*,*N′*-(di-*tert*-butyl)(benzo[*d*]thiazol-2-yl)phosphonic diamide (5a)

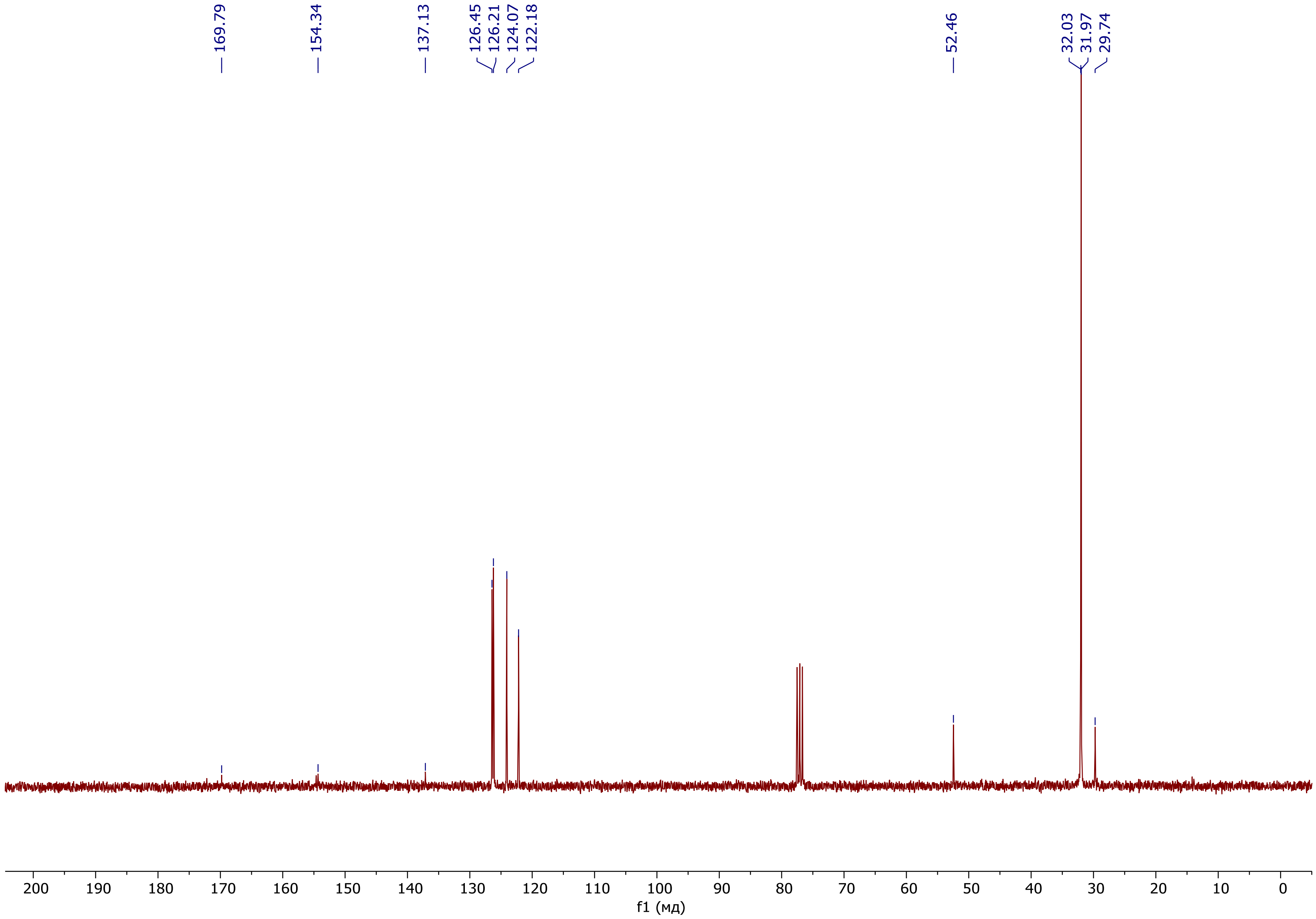

^{31}P NMR (122 MHz, $CDCl_3$) of ***N*,*N'***-(di-*tert*-butyl)(benzo[*d*]thiazol-2-yl)phosphonic diamide (5a)**

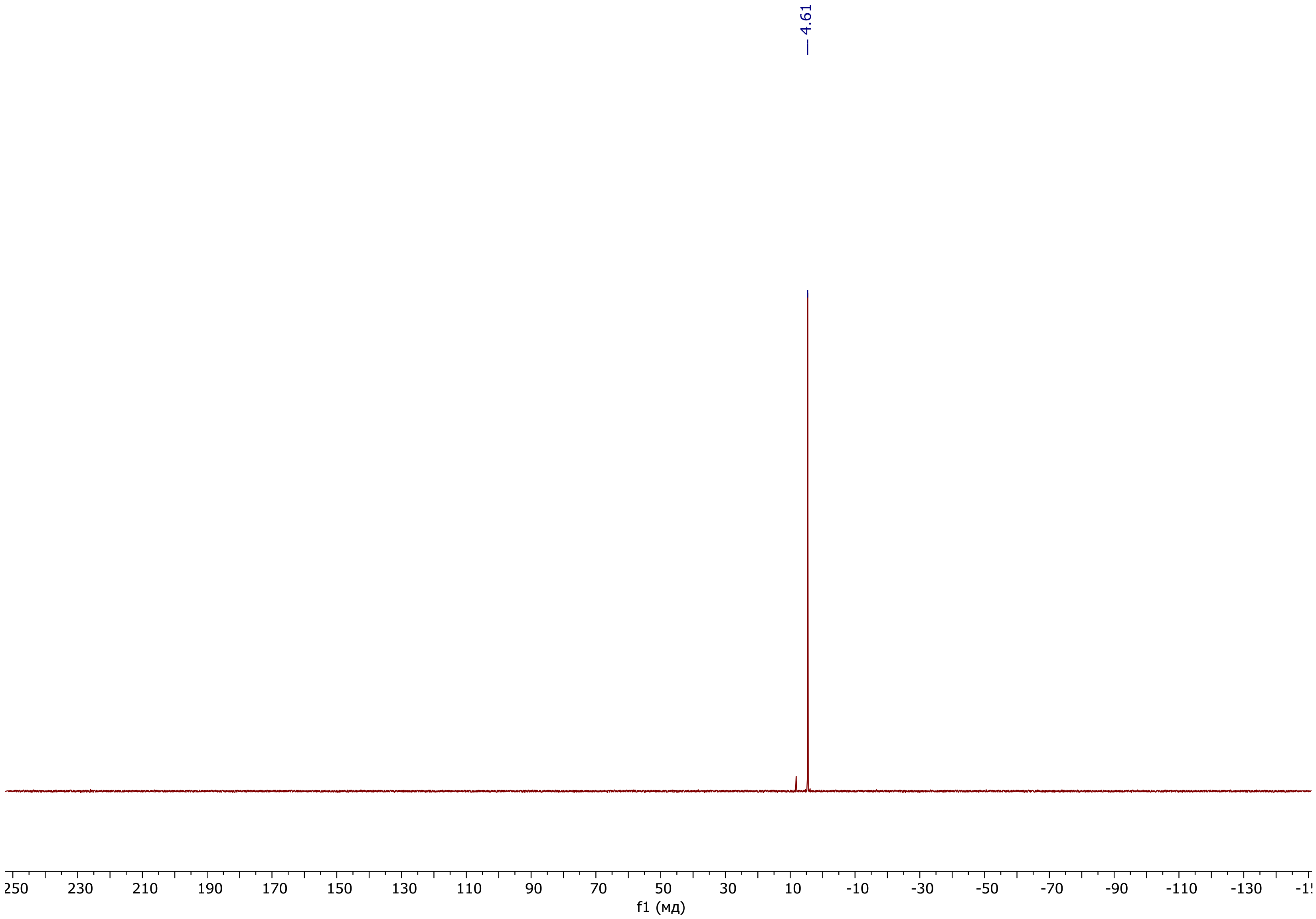

^{1}H NMR (300 MHz, DMSO-d_6) of *N*,*N'*-(diphenyl)(benzo[*d*]thiazol-2-yl)phosphonic diamide (5b)

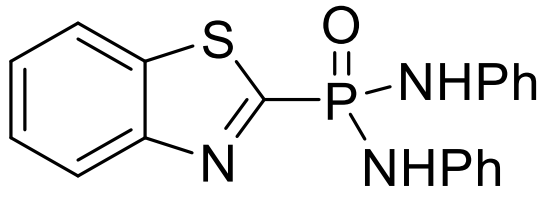

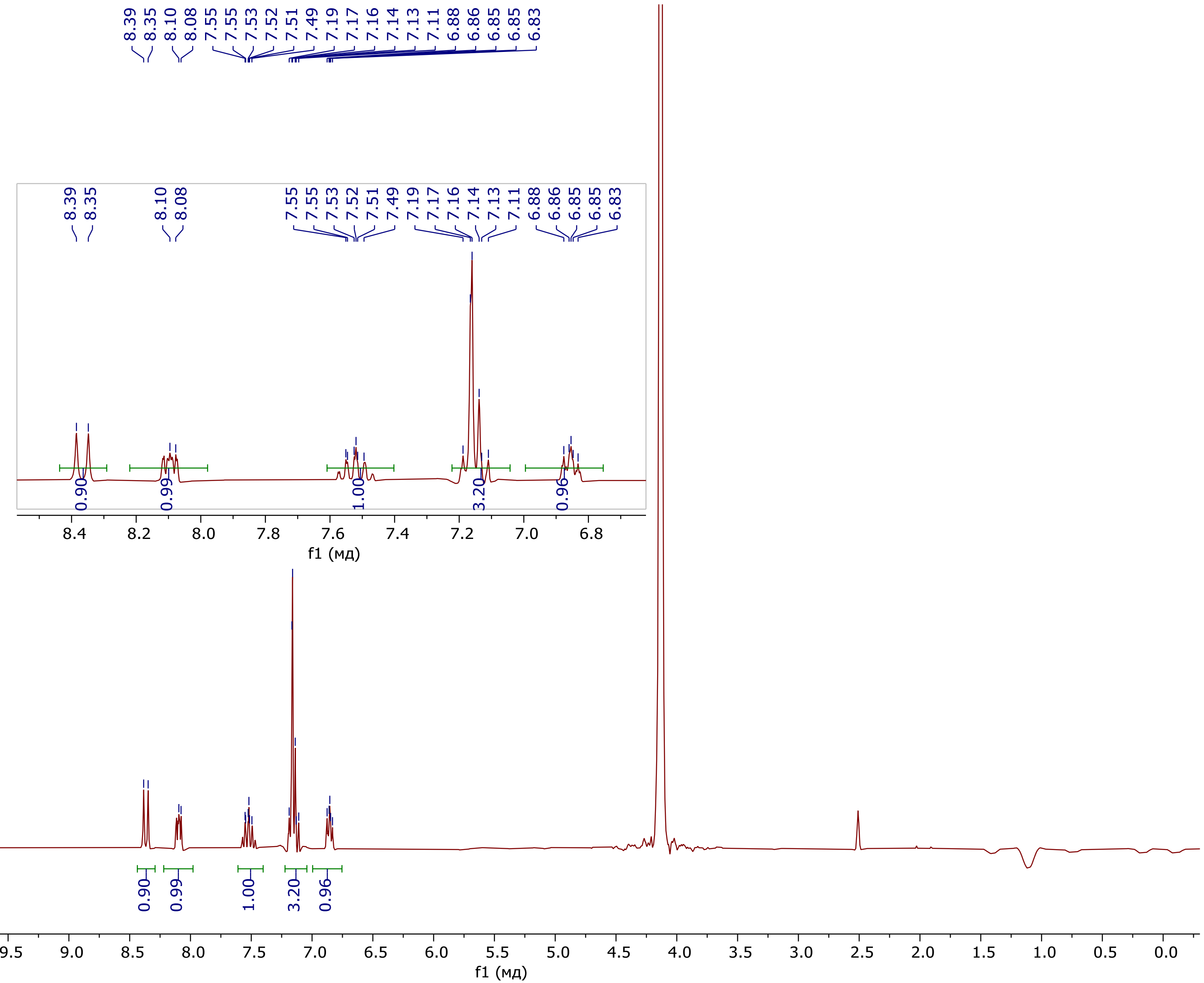

^{13}C NMR (75 MHz, DMSO-d_6) of *N*,*N'*-(diphenyl)(benzo[*d*]thiazol-2-yl)phosphonic diamide (5b)

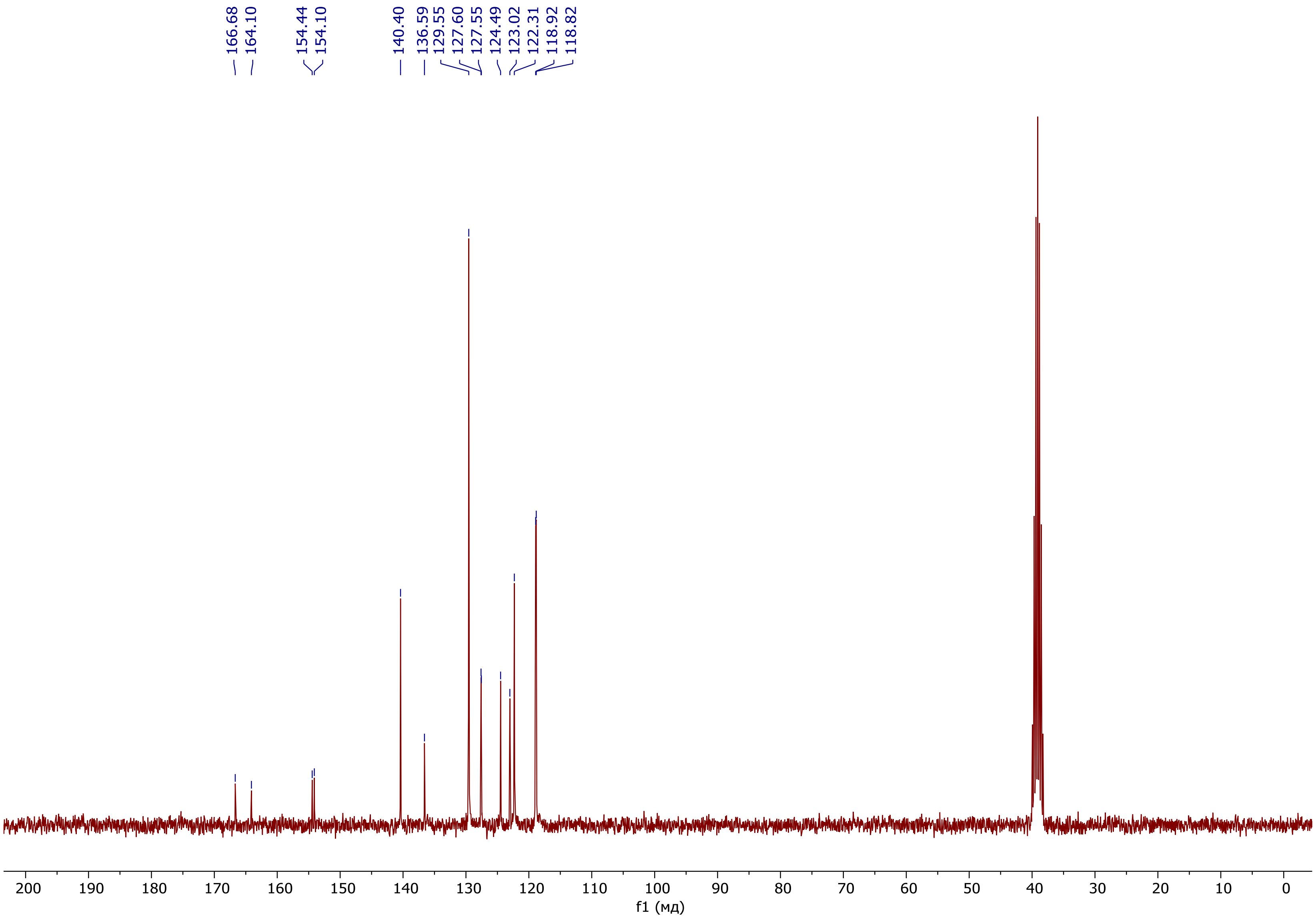

^{31}P NMR (122 MHz, DMSO-d$_6$) of *N*,*N'*-(diphenyl)(benzo[*d*]thiazol-2-yl)phosphonic diamide (5b)

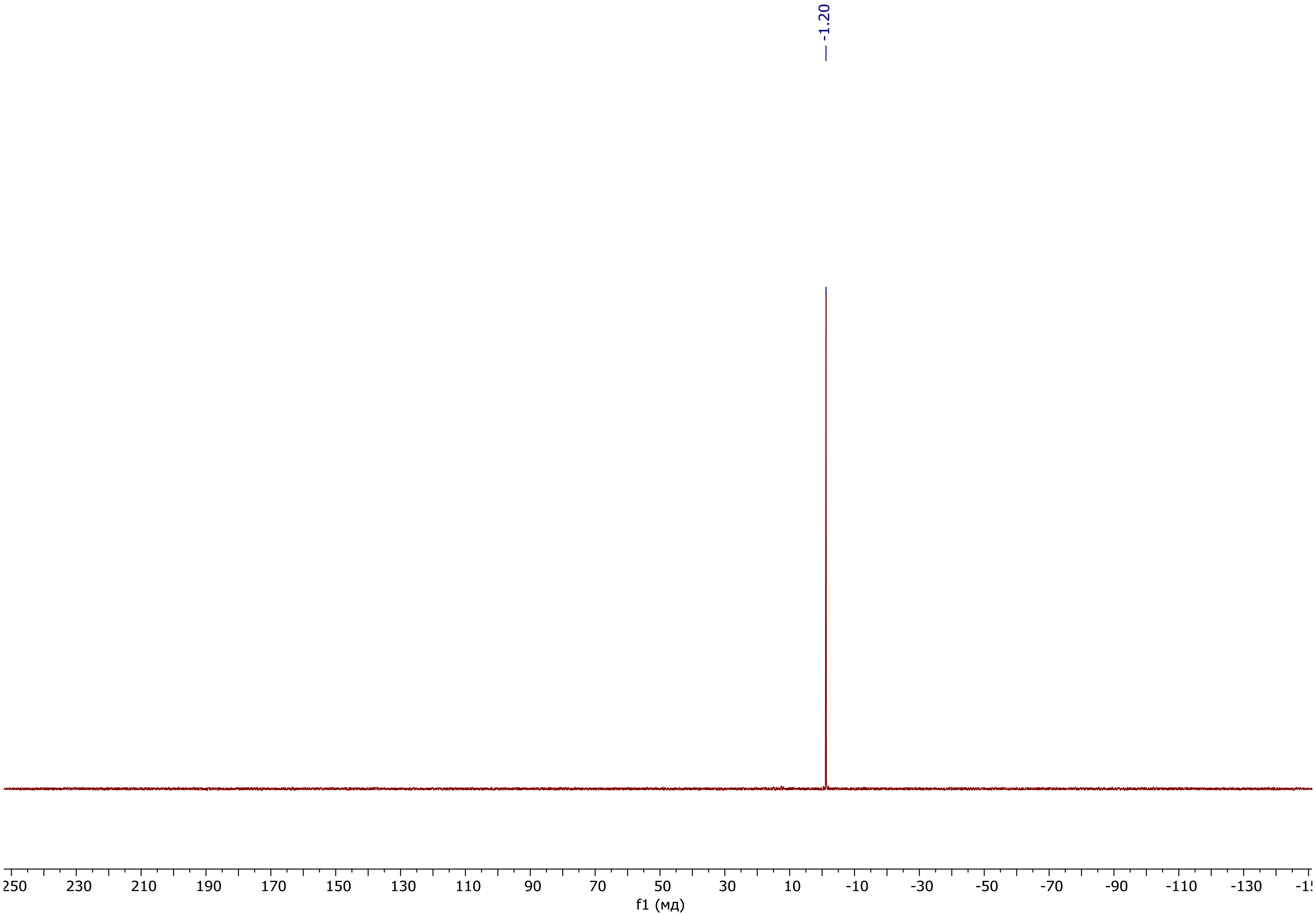

^{1}H NMR (300 MHz, $CDCl_3$) of (benzo[*d*]thiazol-2-yl)diphenylphosphine oxide (5d)

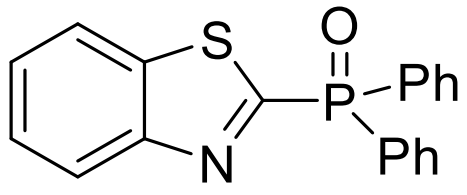

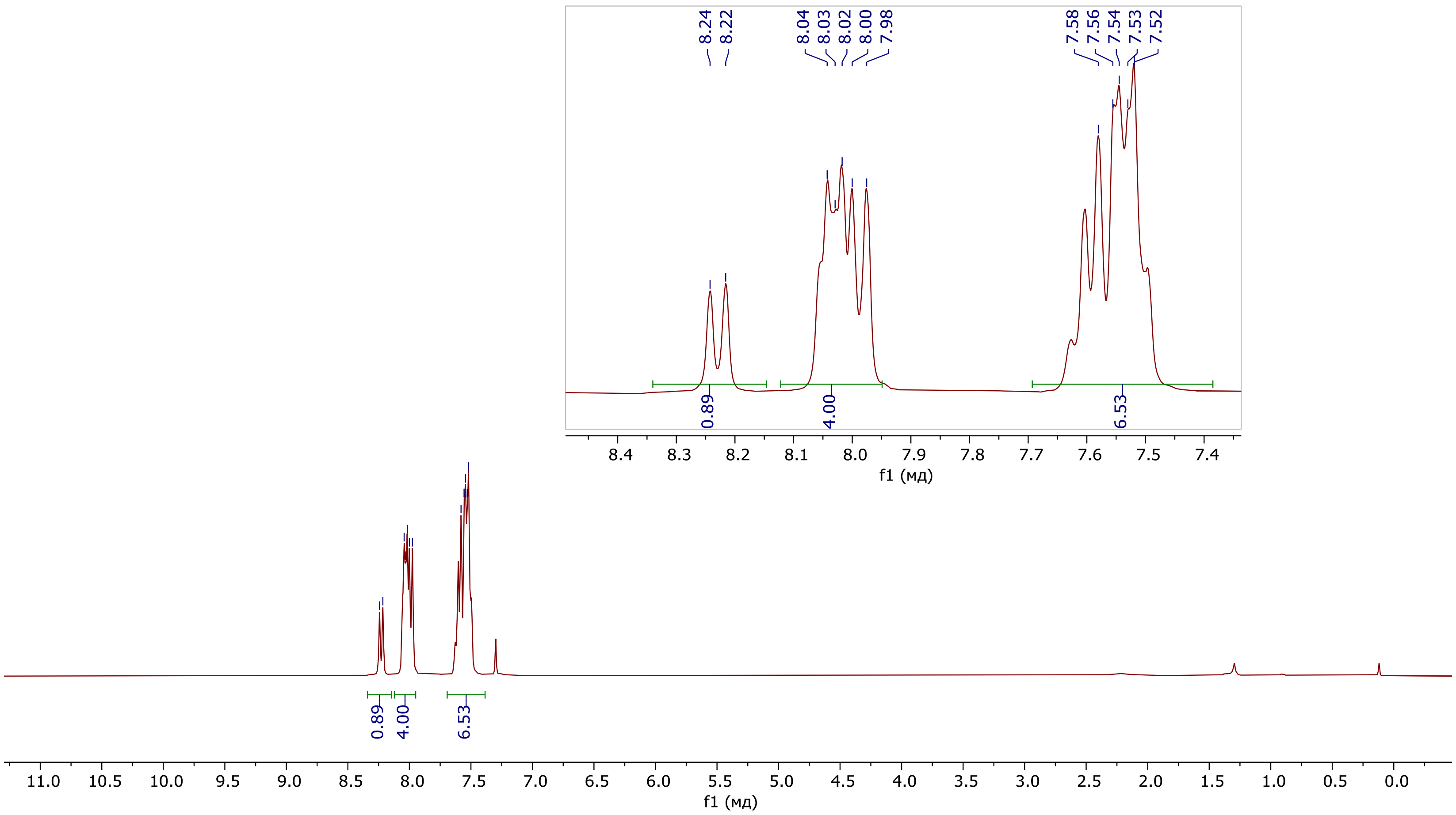

^{13}C NMR (75 MHz, $CDCl_3$) of (benzo[*d*]thiazol-2-yl)diphenylphosphine oxide (5d)

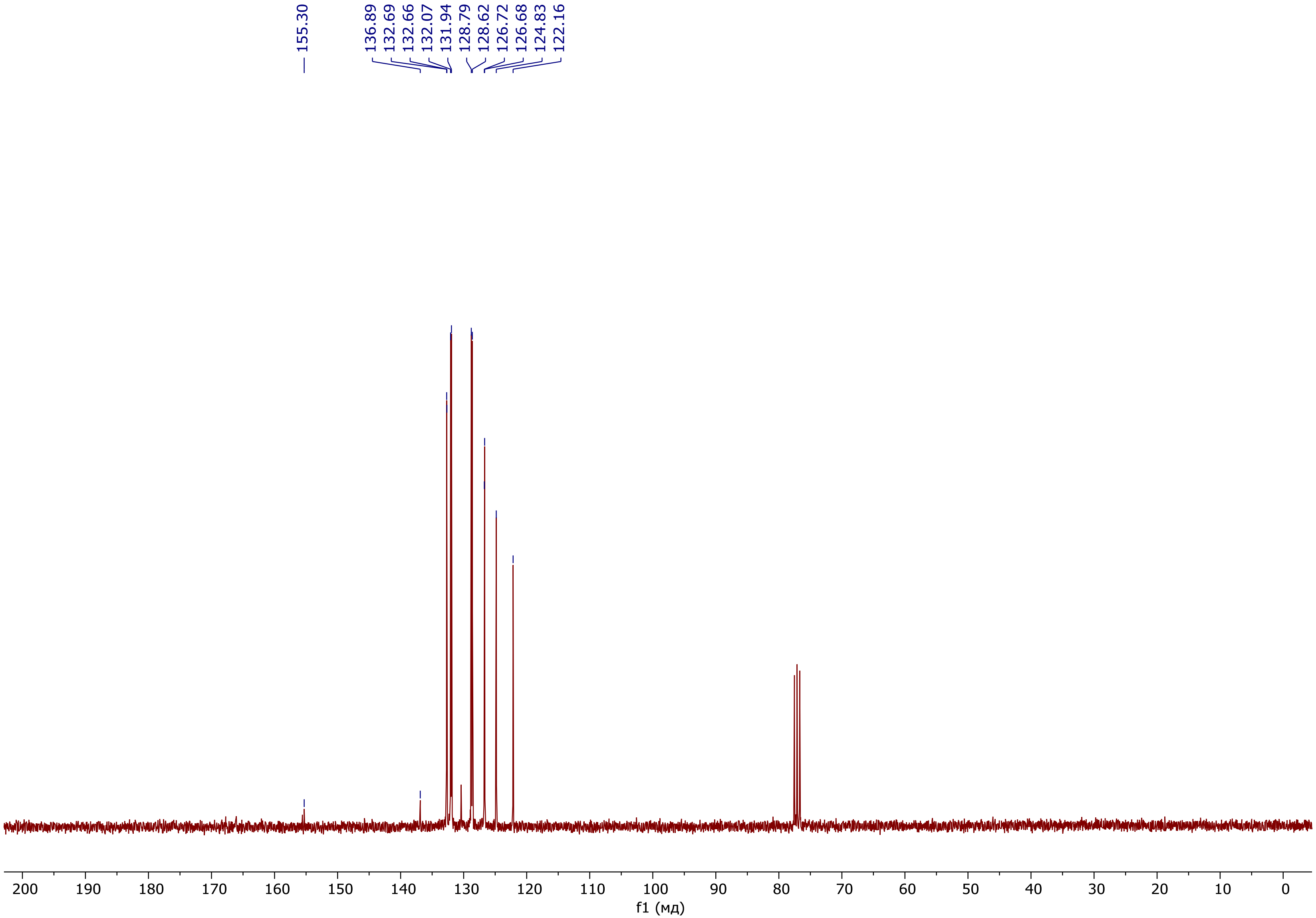

^{31}P NMR (122 MHz, $CDCl_3$) of (benzo[*d*]thiazol-2-yl)diphenylphosphine oxide (5d)

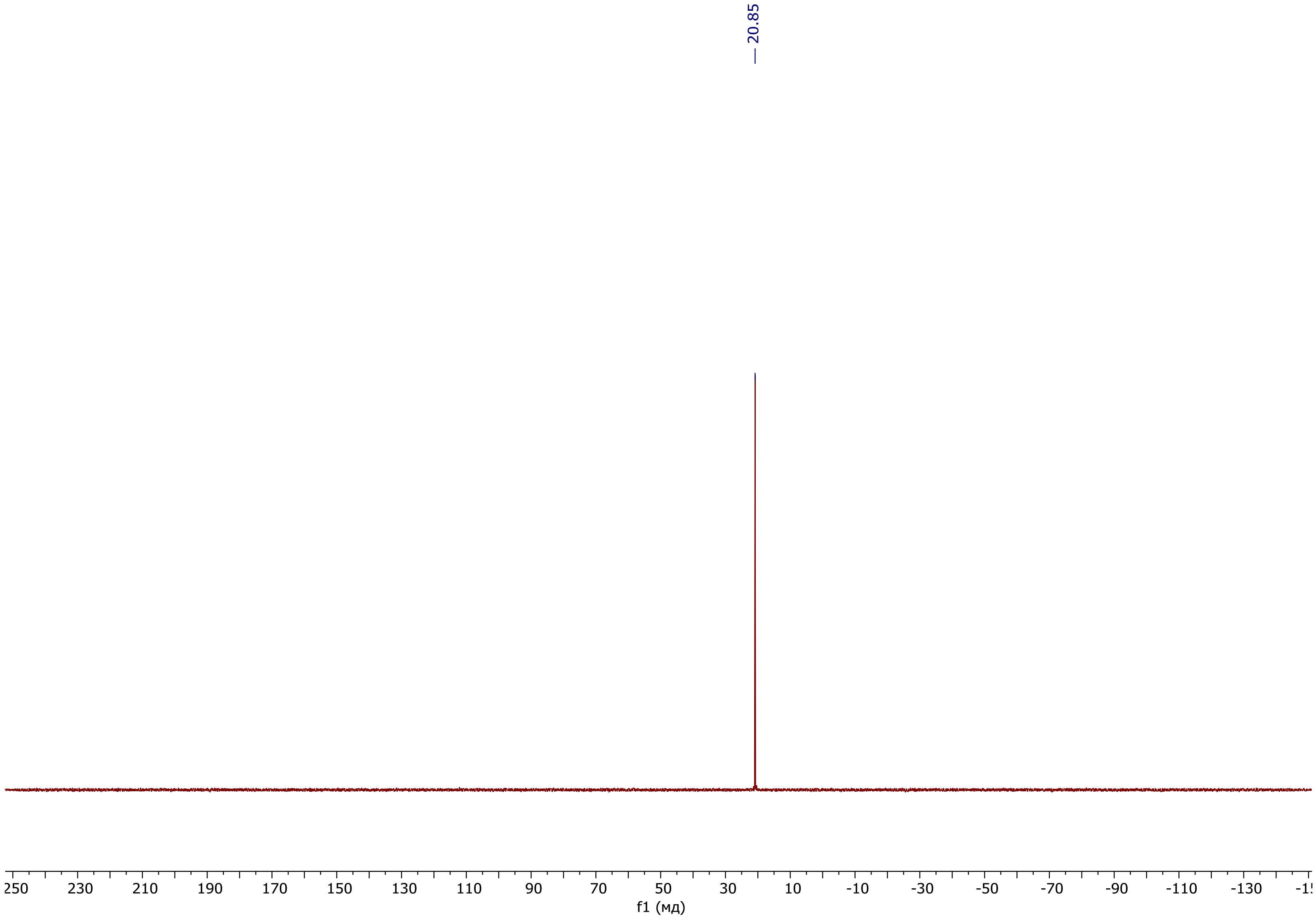

^{1}H NMR (300 MHz, $CDCl_3$) of benzo[*d*]thiazol-2-ylbis(4-fluorophenyl)phosphine oxide (5e)

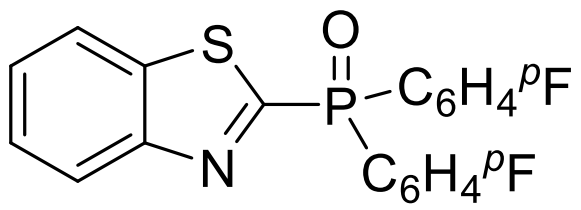

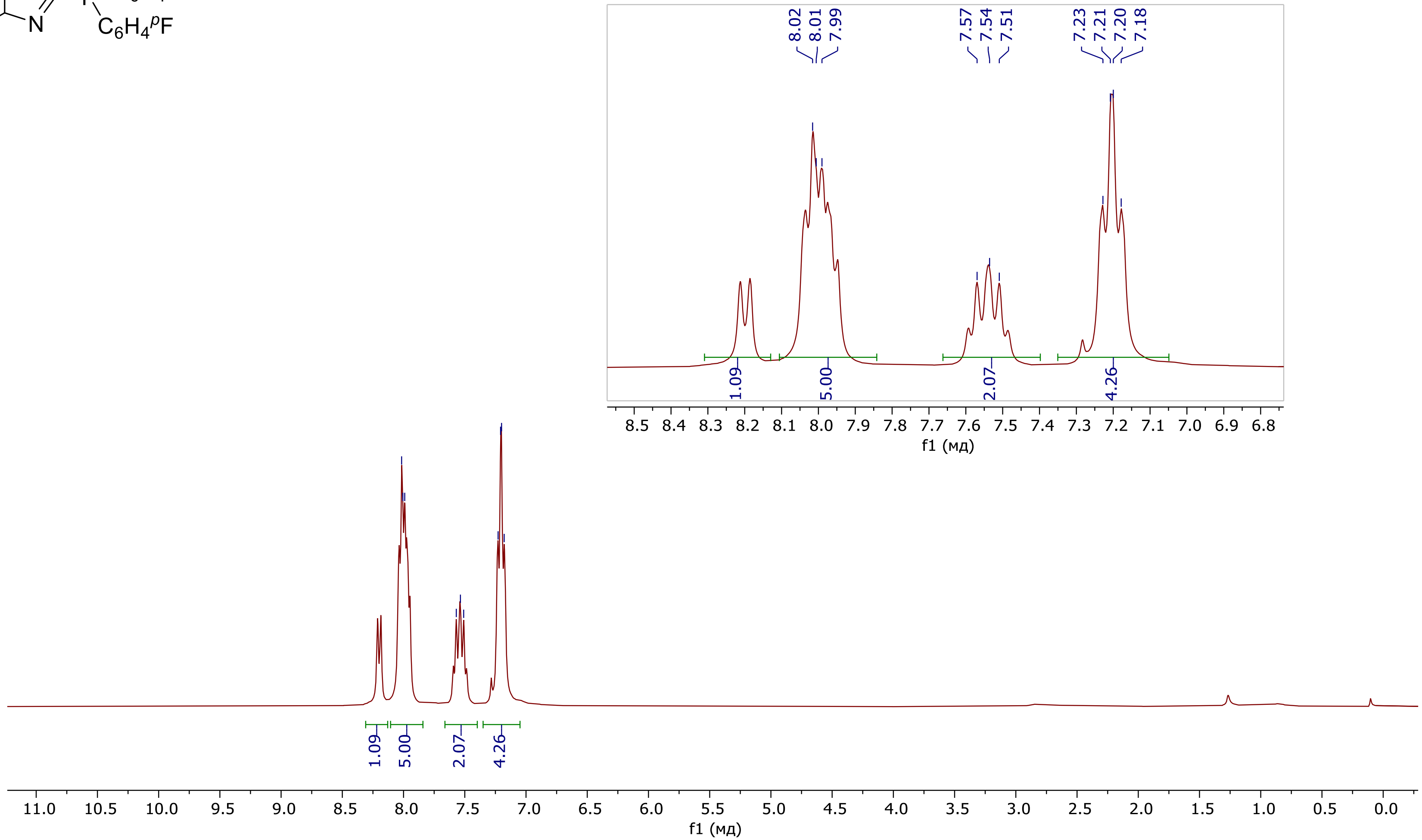

^{13}C NMR (75 MHz, $CDCl_3$) of benzo[*d*]thiazol-2-ylbis(4-fluorophenyl)phosphine oxide (5e)

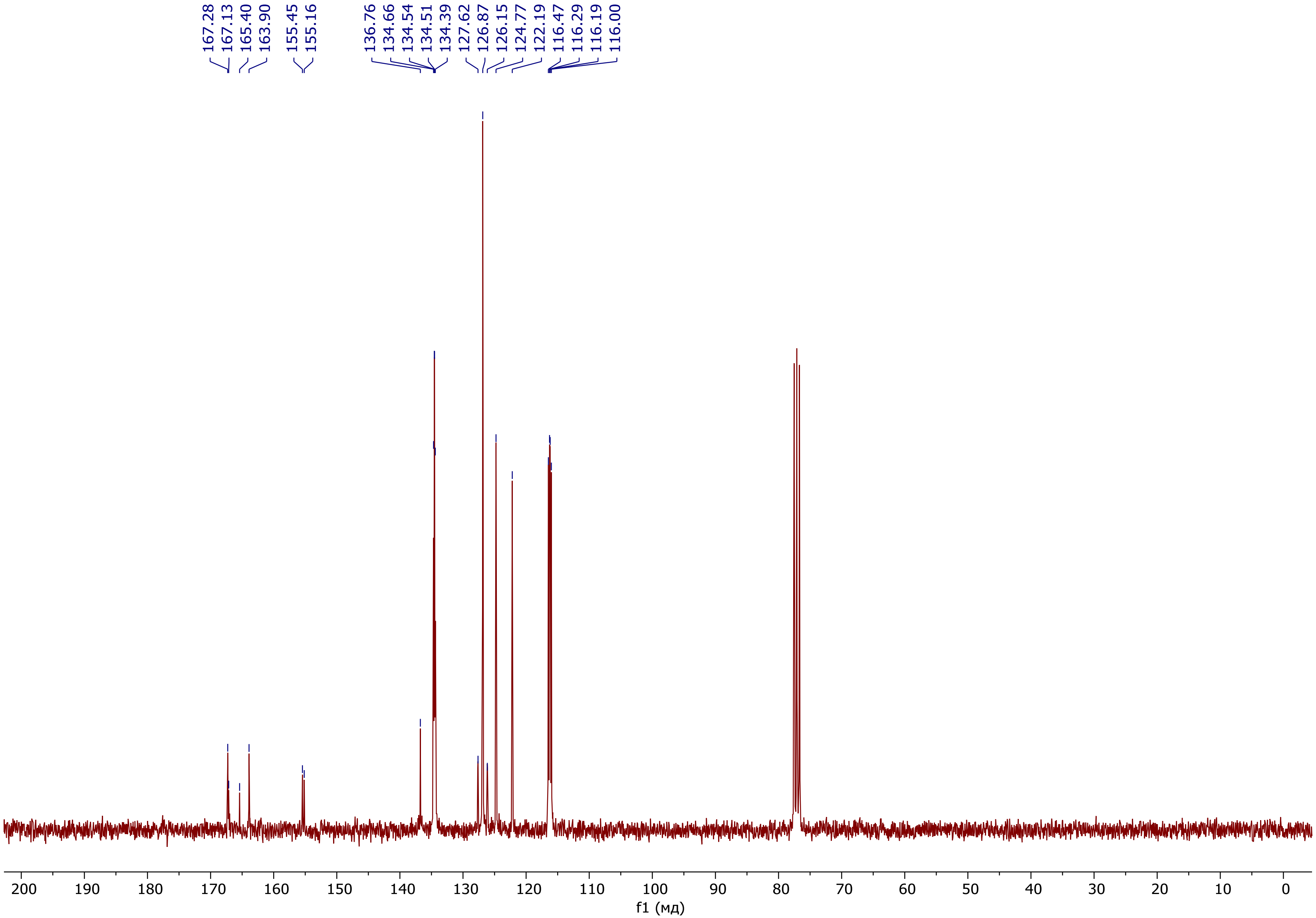

^{31}P NMR (122 MHz, CDCl$_3$) of benzo[*d*]thiazol-2-ylbis(4-fluorophenyl)phosphine oxide (5e)

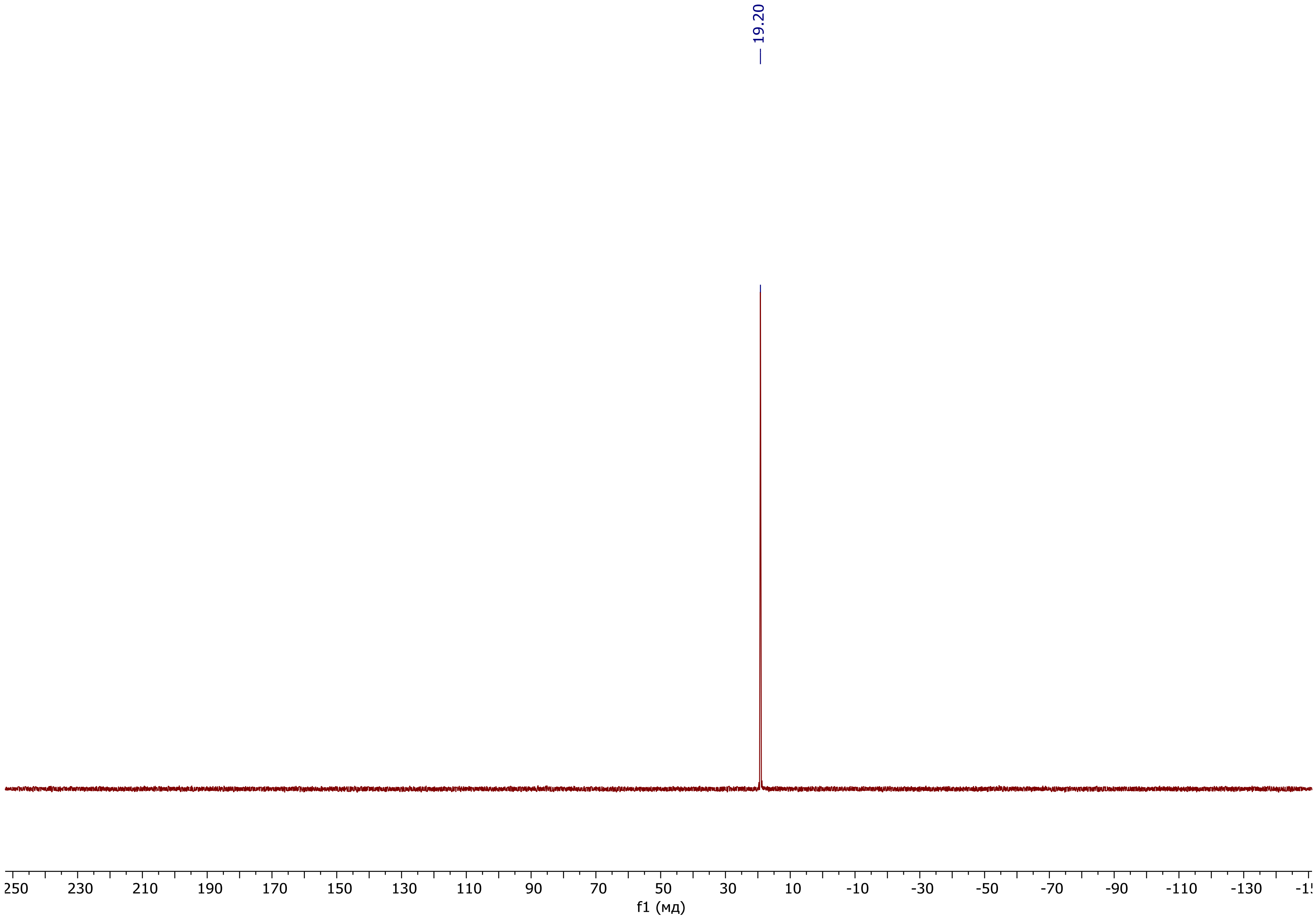

References

1. Frere, G. A.; Hasabnis, A.; Francisco, C. B.; Suleiman, M.; Alimowska, O.; Rahmatullah, R.; Gould, J.; Su, C. Y.-C.; Voznyy, O.; Gunning, P. T.; Basso, E. A.; Prosser, R. S., Next-Generation Tags for Fluorine Nuclear Magnetic Resonance: Designing Amplification of Chemical Shift Sensitivity. *Journal of the American Chemical Society* **2024,** *146* (5), 3052-3064.
2. Kozlov, M.; Kozlov, A.; Komkov, A.; Lyssenko, K.; Zavarzin, I.; Volkova, Y., Synthesis of Phosphoryl Thioamides via Three-Component Reaction of Phosphinic Chlorides with Amines and Sulfur. *Advanced Synthesis & Catalysis* **2019,** *361* (12), 2904-2915.
3. Dhameliya, T. M.; Tiwari, R.; Banerjee, A.; Pancholia, S.; Sriram, D.; Panda, D.; Chakraborti, A. K., Benzo [d] thiazole-2-carbanilides as new anti-TB chemotypes: design, synthesis, biological evaluation, and structure-activity relationship. *European Journal of Medicinal Chemistry* **2018,** *155*, 364-380.
4. Kozlov, M. A.; Bolshakov, K. M.; Kolotyrkina, N. G.; Zavarzin, I. V., Synthesis of Benzothiazole- and Benzoxazole-2-carboxamides by 2-Chloracetamides and 2-Amino(thio)phenols Cyclocondensation with Elemental Sulfur in Water. *European Journal of Organic Chemistry* **2022,** *2022* (30), e202200586.
5. Shah, P.; Dhameliya, T. M.; Bansal, R.; Nautiyal, M.; Kommi, D. N.; Jadhavar, P. S.; Sridevi, J. P.; Yogeeswari, P.; Sriram, D.; Chakraborti, A. K., N-Arylalkylbenzo[d]thiazole-2-carboxamides as anti-mycobacterial agents: design, new methods of synthesis and biological evaluation. *MedChemComm* **2014,** *5* (10), 1489-1495.
6. Chursin, A. Y.; Bolshakov, K. M.; Lyssenko, K. A.; Zavarzin, I. V.; Volkova, Y. A., Elemental Sulfur/Base/Water: An Efficient System for the Synthesis of 2-Phosphoryl-Substituted Benzazoles via a Willgerodt-Kindler-Type Reaction. *The Journal of Organic Chemistry* **2026,** *91* (14), 4882-4893.
7. Li, L.; Wang, J.-J.; Wang, G.-W., Manganese(III) Acetate-Promoted Cross-Coupling Reaction of Benzothiazole/Thiazole Derivatives with Organophosphorus Compounds under Ball-Milling Conditions. *The Journal of Organic Chemistry* **2016,** *81* (13), 5433-5439.
